# Composition of immune cells in peripheral blood of patients with leukemia in remission after stem cell transplantation (HSCT) with or without graft-versus-host disease (GvHD): a comparative analysis with healthy donors

**DOI:** 10.3389/fimmu.2026.1934726

**Published:** 2026-10-23

**Authors:** Vivien M. Simon, Cansu Karatas, Kristina Schreiner, Anying Huang, Annalena Frischhut, Joudi Abdulmajid, Christoph Schmid, Jochen J. Frietsch, Helene Schmeller, Tobias A. W. Holderried, Elisa Sala, Helga Schmetzer, Giuliano Filippini Velazquez, Jan Weller

**Affiliations:** 1 Department of Medicine III, University Hospital Ludwig-Maximilians-Universität (LMU) Munich, Munich, Germany; 2 Bavarian Cancer Research Center (BZKF), Augsburg, Germany; 3 Faculty of Biology, University of Bielefeld, Bielefeld, Germany; 4 Section for Stem Cell Transplantation and Cellular Therapy Research, Faculty of Medicine, University of Augsburg, Augsburg, Germany; 5 Comprehensive Cancer Center Alliance WERA (CCC WERA), Augsburg, Germany; 6 Department of Internal Medicine II, University Hospital of Würzburg, Würzburg, Germany; 7 University of Bonn, University Hospital Bonn, Medical Clinic and Polyclinic III Internal Medicine with a focus on Oncology, Hematology, and Rheumatology, Bonn, Germany; 8 Clinical Division of Hematology, Department of Internal Medicine, Medical University of Graz, Graz, Austria; 9 Department of Hematology and Oncology, University Hospital Ulm, Ulm, Germany; 10 Department of Oncology, Hematology and Bone Marrow Transplantation with Division of Pneumology, University Medical Center Hamburg-Eppendorf, Hamburg, Germany

**Keywords:** leukemia, HSCT, GvHD, immune-monitoring, immune-cell-subset, immune-phenotyping, immune-dysregulation, flow-cytometry

## Abstract

**Background:**

Allogeneic hematopoietic stem cell transplantation (HSCT) is potentially curative for many hematologic malignancies. Clinical outcomes are critically determined by the balance between graft-versus-malignancy effects and graft-versus-host disease (GvM and GvHD, respectively). We hypothesized that integrated immune phenotyping can distinguish immune tolerance from clinically relevant alloreactivity in patients with hematological neoplasia who are in remission after HSCT. These findings may have implications for GvHD screening and monitoring and could support treatment decision-making.

**Methods:**

Multi-color flow cytometry of peripheral blood mononuclear cells profiled myeloid, adaptive, and innate compartments in healthy donors (*n* = 10) and post-HSCT patients in remission: without GvHD [NoGvHD (*n* = 19)], with chronic (c) GvHD (*n* = 18, including *n* = 4 overlap-a/cGvHD), and with acute (a) GvHD (*n* = 6). Frequencies of immune cell subsets were compared between cohorts and stratified by clinical features [e.g., severity, organ involvement, donor lymphocyte infusion (DLI), GvHD development, and cGvHD progression].

**Results:**

NoGvHD patients (clinically asymptomatic and whose disease were in remission) showed immune-cell composition differing substantially from healthy donors: CD14^+^ monocytes (Mo), M-MDSCs, and especially checkpoint molecule (cpm)-expressing Mo (TIM3, 2B4, and PDL1) were reduced, while CTLA-4^+^ polymorphonuclear myeloid-derived suppressor cells (CTLA-4^+^-PMN-MDSCs) were elevated, as well as T-effector memory (Tem) cells and cpm-expressing T cells (PD1, LAG3, 2B4, TIM3, and TIGIT). cGvHD showed eroded regulatory myeloid subsets and skewed T cells toward effector-memory dominance with increased frequencies of inhibitory cpm-expressing T cells. aGvHD showed marked late-proliferating T-cell expansion (T_prol-late_/T^3+^ fold change 9.92 vs. Healthy), T_reg_ depletion, and loss of regulatory NK^56bri^ populations. Overlap a/cGvHD displayed chronic T-cell stimulation with altered tissue homing (Intβ7^+^ enriched), and DLI-induced GvHD showed effector differentiation with insufficient regulatory compensation.

**Conclusion:**

NoGvHD is not “Healthy minus disease” but a distinct post-HSCT immune state. Multi-compartment immune signature profiling by flow cytometry distinguishes preserved tolerance (Healthy) from post-HSCT remodeling (NoGvHD), progressive dysregulation (cGvHD), and acute hyperactivation (aGvHD), and refines GvHD subgroups by clinical context.

## Introduction

### Hematological malignances and hematopoietic stem cell transplantation

Hematological malignancies, including acute leukemia and myelodysplastic syndromes, are characterized by uncontrolled proliferation of immature precursors and disrupted hematopoiesis (1–3). For eligible patients with high relapse risk, allogeneic hematopoietic stem cell transplantation (HSCT) remains the only potentially curative option (4, 5). Beyond disease biology and stage, stem cell donor type and source selection [human leucocyte antigen (HLA) matching], conditioning intensity, early hematological reconstitution, and graft-versus-host disease (GvHD) are factors influencing clinical outcomes (6, 7). In particular, GvHD and infections are severe complications after HSCT, representing a significant clinical challenge due to their association with transplant-related mortality (TRM) with 15%–30% incidence during the first years after HSCT (8).

### Graft-versus-host disease

GvHD is a post-HSCT clinical syndrome mediated by alloreactive donor-derived immune cells. Historically, GvHD was classified according to its time of onset after HSCT, with acute GvHD (aGvHD) and chronic GvHD (cGvHD) distinguished by a 100-day cutoff. More recently, aGvHD and cGvHD have been recognized as biologically distinct entities with characteristic clinical manifestations independent of the time of onset. aGvHD primarily targets skin, the gastrointestinal (GI) tract, and liver and develops via tissue damage, immune activation, and effector cytotoxicity (9, 10). cGvHD can follow aGvHD or arise *de novo*, affects multiple organs, and shows autoimmune-like features driven by T- and B-cell dysregulation, fibrosis, and impaired tissue repair (11–13). Overlap GvHD, in which clinical acute and chronic features co-occur, accounts for approximately 30%–40% of cGvHD cases and is associated with poorer survival (14). Treatment of GvHD (acute and chronic) requires systemic corticosteroids as the cornerstone of first-line therapy, typically administered while the calcineurin-inhibitor-based immunoprophylaxis is continued or as a single treatment. Approximately one-third of patients develop steroid-refractory or steroid-dependent disease; in these cases, second-line and subsequent options include novel drugs such as ruxolitinib (JAK1/2 inhibition), belumosudil (ROCK2 inhibition), mTOR inhibitors, extracorporeal photopheresis (ECP), and B-cell-directed strategies, selected to control alloreactivity while preserving the graft-versus-malignancy/-leukemia (GvM/GvL) effect (15–17). Currently, treatment selection is not routinely biomarker-guided but is largely determined by drug availability, clinical presentation, prior therapies, and physician expertise. Despite these therapeutic advances, GvHD remains a major clinical challenge.

### Immune cells and their role on GvHD

Despite extensive characterization of individual immune compartments after HSCT, comprehensive integrated phenotyping, which captures the simultaneous behavior of myeloid, adaptive, and innate cells across clinically defined GvHD subgroups, remains limited. Existing studies have largely focused on single lineages or single time points, leaving open how regulatory and effector networks interact when GvHD develops, persists, or is absent (18). Delayed immune reconstitution after HSCT has been linked to an increased risk of cGvHD, infections, and higher TRM, highlighting the clinical need for cell-resolved monitoring strategies that can be integrated into routine post-HSCT care (19). An integrated view across compartments is helpful to distinguish post-HSCT tolerance from subclinical pathological states and to identify cell-level signatures that may anticipate clinical trajectory; in particular, it is not completely described whether patients in sustained remission after HSCT and without GvHD after tapering of immunosuppression (NoGvHD) already differ quantitatively from healthy donors at the level of regulatory cell networks, and whether GvHD development can be predicted from baseline immune composition.

### Aims

With a special focus on activated, inflammatory, damaging, or tolerogenic/suppressive myeloid, adaptive, and innate immune cells, we investigated quantitative and qualitative differences in myeloid, adaptive, and innate cell blood compartments between post-HSCT patients whose disease was in sustained remission compared to patients with aGvHD or cGvHD, and compared them with healthy donors. By correlating immunological findings with clinical parameters, we aimed to identify signatures that distinguish tolerance from overshooting alloreactivity and inform stratification, monitoring, and potentially therapy.

## Material and methods

### Sample acquisition

Peripheral blood was collected at the University Hospitals of Augsburg, Bonn, Ulm, Würzburg, and the LMU Hospital Munich into lithium-heparin tubes (S-Monovette, SARSTEDT, Nümbrecht, Germany). Informed consent followed the Declaration of Helsinki and the LMU Munich ethics committee (vote 21-0544). Samples were filtered (70 µm pluriStrainer) and diluted in PBS, and peripheral blood mononuclear cells (PBMCs) were enriched by BioColl^®^ density-gradient centrifugation (465 *g*, 20 min). PBMCs were resuspended in RPMI 1640 + GlutaMAX™-I and washed (423 *g*, 7 min) before staining.

### Cohorts

For analysis, we included healthy donors [H: *n* = 10; mean age: 51.7, range (r): 22–85] and post-HSCT patients whose disease was in complete remission and had no signs of infection (*n*=43). Patients without GvHD [NoGvHD, *n* = 19, mean age: 62.8 years (r: 20–77)]. The median time from HSCT to the day of analysis was 11.4 months (r: 3.2–24). Patients with aGvHD [*n* = 6; mean age: 62.2 years (r: 33–74)] presented with skin (*n* = 3) or GI (*n* = 3) GvHD; median time since HSCT: 3.2 months (r: 1.4–5.7); all but two patients were receiving standard immunosuppression, including corticosteroids, at the time of analysis. Patients with cGvHD [*n* = 18; mean age: 53.7 years (r: 18–79)], including overlap a/cGvHD (*n* = 4), presented with severe (*n* = 10) or mild/moderate (*n* = 8) grade of cGvHD, and 16 patients were receiving immunosuppressive treatment, including corticosteroids (*n* = 12), ruxolitinib (*n* = 10), or a calcineurin inhibitor (*n* = 8); time since HSCT: 18.7 months (r: 7.9–98.1). Patient and HSCT characteristics, as well as organs affected, are shown in **Table 1**. For clinical correlation, sub-cohort analyses compared severe vs. mild/moderate cGvHD; *de novo*/quiescent vs. progressive cGvHD; single- vs. multi-organ cGvHD; donor lymphocyte infusion (DLI)-induced vs. non-induced cGvHD; and overlap vs. classic cGvHD. Exploratory analyses compared baseline NoGvHD samples of patients who did or did not subsequently develop GvHD or disease relapse. Comparisons between cohorts compare the second with the first reference cohort (e.g., H/cGvHD, frequencies of cells in cGvHD patients compared to the H cohort were increased/decreased).

**Table 1:** Patient and transplant characteristics.

Characteristic		Healthy (*n* = 10)	NoGvHD (*n* = 19)	cGvHD (*n* = 18)^a^	aGvHD (*n* = 6)
Sex, male/female	Male	4	15	11	5
	Female	6	4	7	1
Age, mean (range), years		51.7 (22–85)	62.8 (20–77)	53.7 (18–79)	62.2 (33–74)
Initial diagnosis	AML	–	13	11	3
	ALL		1	3	0
	MDS		4	4	2
	MPN		1	0	1
Donor type (MRD/MUD/MMUD/haploidentical)	MRD	–	3	1	0
	MUD		12	10	2
	MMUD		0	3	2
	Haploidentical		4	4	2
Conditioning (MAC/RIC)	MAC	–	0	4 ^B^	0
	RIC		19	13	6
Time from SCT to sampling, median (range), months		–	11.4 (3.2–24)	18.7 (7.9–98.1)	3.2 (1.4–5.7)
GvHD grade (severe/mild–moderate)	Severe	–	–	10	– ^C^
	Mild/Moderate			8	
cGvHD course (*de novo*–quiescent/progressive)	*De novo*/Quiescent	–	–	13	–
	Progressive			5	
Organ involvement (skin/GI/liver/lung)	Skin	–	–	11	3
	GI			7	4
	Liver			3	0
	Lung			6	0
Immunosuppression at sampling (corticosteroids/CNI/ruxolitinib)^d^	Corticosteroids	–	0	12	4
	CNI		0	8	3
	Ruxolitinib		1	10	2

Counts unless stated. MAC/RIC, myeloablative/reduced-intensity conditioning; MRD/MUD/MMUD, matched related/matched unrelated/mismatched unrelated donor; CNI, calcineurin inhibitor; –, not applicable.

^a^ Includes four overlap acute/chronic GvHD patients. ^b^Conditioning not documented for one cGvHD patient. ^c^Acute-GvHD grades per patient in **Supplementary Table S10c**. ^d^Categories not mutually exclusive (combination therapy); no immunosuppression in 18 NoGvHD and 2 aGvHD patients.

GvHD, graft versus host disease; a, acute; c, chronic; AML, acute myeloid leukemia; ALL, acute lymphatic leukemia; MDS, myelodysplastic syndrome; MPN, myeloproliferative neoplasia; MRD, matched-related donor; MUD, matched-unrelated donor; MMUD, mismatched-unrelated donor; MAC, myeloablative conditioning; RIC, reduced intensity conditioning; SCT, stem cell transplantation; GI, gastrointestinal tract; CNI, calcineurin inhibitor.

### Flow cytometry

Cells were stained with FITC-, PE-, PC7-, or APC-labeled monoclonal antibodies (Beckman Coulter, Krefeld; Becton Dickinson, Heidelberg; BioLegend, Koblenz, Germany; isotype controls included). Acquisition used a FACSCalibur with CellQuestPro v5.1 and a uniform gating strategy as previously described (20). The cell-by-cell background and the cells used in the study are provided in **Supplementary Background S1**. Throughout, marker-positive subsets are written as numerator/denominator, with the cell population preceding the slash and its reference (parent) population following it: for example, PD-L1^+^Mo/Non Lym denotes PD-L1^+^ monocytes as a fraction of the Non-Lymphocyte gate (Gate 1), whereas PD-L1^+^Mo/Mo denotes the same cells as a fraction of all monocytes (Gate 2). Cell subtypes, their abbreviations, and marker definitions are provided in **Supplementary Table 1**. Antibody clones and fluorochromes are included in the same table.

### Statistics

Data were analyzed with FlowJo v10.10.0; samples with <30 events in the relevant gate were excluded. Plots were generated in GraphPad Prism 10 and R v4.3.2 with the tidyplots package (21). Mean ± SD are shown. Group comparisons used Mann–Whitney *U*, Kruskal–Wallis, and Levene’s *t*-test. *p* ≤ 0.005 was highly significant (***), *p* ≤ 0.05 was significant (**), and *p* ≤ 0.1 was borderline (*). In fold change analyses (control/second group), the cohort listed in front of the slash served as reference. *p*-values are uncorrected for multiple testing. Manuscript-wide cell-name consistency was verified using Claude (Anthropic, PBC, 548 Market Street, PMB 90375, San Francisco, CA 94104, USA; https://www.anthropic.com).

Comprehensive biomedical literature retrieval and reference verification (citation lookup, DOI/PMID conversion, and full-text search across PubMed Central, bioRxiv, and medRxiv) were performed via paperclip (GXL.ai, version 0.3.0; https://paperclip.gxl.ai/), a command-line tool providing search and analysis access to over 8 million biomedical papers.

## Results

### Frequencies of immune cell subtypes in healthy, NoGvHD, cGvHD, and aGvHD

As described in Material and Methods, using a panel of 120 cell subsets (**Supplementary Background**; **Supplementary Table 1**), we performed quantitative analyses of the subsets identified in the peripheral blood of patients across previously defined clinical cohorts, whereby healthy donors (H) were taken as baseline, and were compared to patients with NoGvHD, cGvHD, and aGvHD, respectively. The differences (relative to H and NoGvHD, respectively) are shown as fold changes (either decreased or increased) in **Figure 1** (significant values) and **Supplementary Figure 2** (all data). In the following paragraphs, we describe these observations in the myeloid, adaptive, and innate compartments.

**Figure 1:**
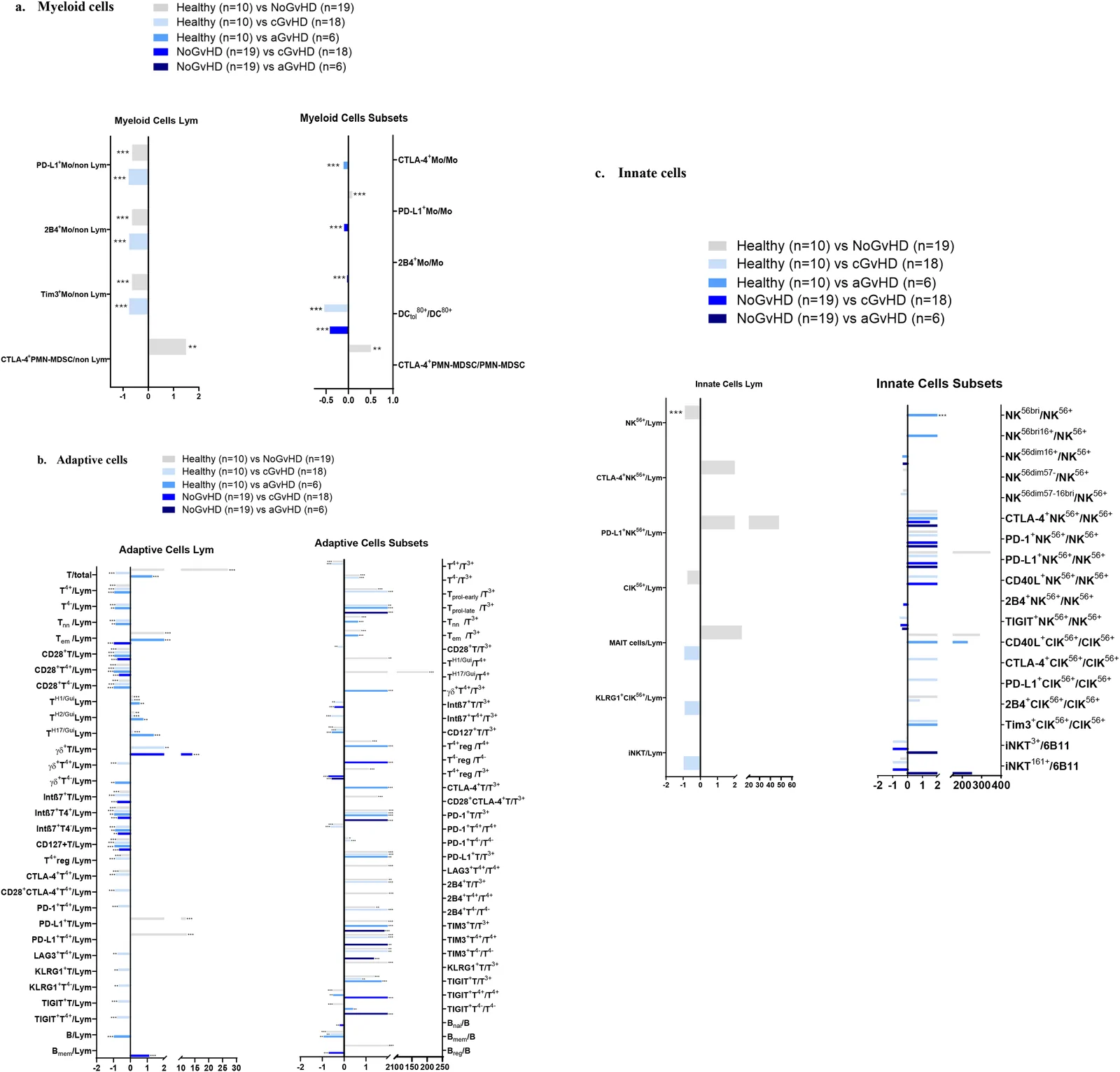
Fold changes of cell-subset frequencies with significant differences across cohorts (Healthy, NoGvHD, cGvHD, and aGvHD). Pairwise fold changes of frequencies of myeloid **(a)**, adaptive **(b)**, and innate **(c)** cells, referenced to non-Lym or Lym (left) or to specific subsets (right), in Healthy (*n* = 10), NoGvHD (*n* = 19), cGvHD (*n* = 18), and aGvHD (*n* = 6). For the extensive data analysis (including no significant changes), please see **Supplementary Figure 2**.

### Myeloid cells

Myeloid cells were quantified in two different gates, a Non-Lymphocyte gate, and one gate referred to myeloid subsets (**Supplementary Table 1**).

Healthy donors showed the strongest myeloid regulatory networks; in contrast, checkpoint molecule (cpm)-expressing monocytes (Mo) (TIM3^+^Mo, 2B4^+^Mo, and PDL1^+^Mo), tolerogenic DCs, and M-MDSCs were progressively reduced in NoGvHD and aGvHD/cGvHD (**Figure 1a**; **Supplementary Figure 2a**).

CD14^+^ monocyte frequencies were highest in Healthy (%CD14^+^/Non Lym: H 15.26 ± 9.44; NoGvHD 8.02 ± 3.74; cGvHD 6.71 ± 5.9; aGvHD 5.57 ± 3.27; **Figure 1a**; **Supplementary Figure 2a**). PD-L1^+^, 2B4^+^, and Tim3^+^ monocytes were also enriched in Healthy in the Non-Lymphocyte gate (e.g., %PD-L1^+^Mo/Non Lym H 20.63 ± 11.61 vs. NoGvHD 7.37 ± 4.35; *p* = 0.000152) but comparable when referred to monocytes. Fold-change reductions were significant in NoGvHD compared to H, and cGvHD compared to H (e.g., 2B4^+^Mo/Non Lym fold change −0.65 and −0.76, respectively).

Tolerogenic CTLA-4^+^DC^80+^/DC^80+^ were decreased in cGvHD compared to H (*p* = 0.000013; fold change −0.54) and cGvHD compared to NoGvHD (*p* = 0.000821; fold change −4.17) and increased for cGvHD/aGvHD (fold change 1.28). M-MDSCs were highest in Healthy (%M-MDSC/CD11b^+^CD33^+^: H 58.4 ± 27.73; NoGvHD 30.68; cGvHD 25.69; aGvHD 35.96), with significant reductions in NoGvHD compared to H and cGvHD compared to H (fold changes −0.47 and −0.56). PMN-MDSC frequencies were largely comparable across cohorts, but in particular, CTLA-4^+^PMN-MDSC was significantly increased in NoGvHD compared to H (**Figure 1a**; **Supplementary Figure 2a**).

### Adaptive cells

Adaptive cells were similarly quantified in two different gates, a Lymphocyte gate and another gate referred to adaptive cell subsets (as given in **Supplementary Table 1**).

%T4^+^/Lym were highest in Healthy (24.64 ± 7.91) and decreased to 3.89 in NoGvHD, 1.38 in cGvHD, and 1.04 in aGvHD; T^4−^/T^3+^ were increased in NoGvHD compared to Healthy (fold change 0.72). Activated and non-naïve T cells were reduced in all GvHD vs. Healthy comparisons within the Lymphocyte gate (e.g., T_nn_/Lym in aGvHD compared to H, fold change −0.86). However, these cells were increased in the gate referenced to T cells (T_nn_/T^3+^ in aGvHD compared to H, fold change 0.65), indicating compositional rather than uniform loss. Late-proliferating T cells were highly elevated in aGvHD (T_prol-late_/T^3+^ in aGvHD compared to H, fold change 9.92, *p* < 0.000001). Memory T cells favored effector over central memory; T_em_/T^3+^ rose in aGvHD versus Healthy and in NoGvHD vs. H (fold change 0.75; **Figure 1b**; **Supplementary Figure 2b**).

T^H1^, T^H2^, and T^H17^ frequencies were highest in Healthy and declined progressively across NoGvHD, cGvHD, and aGvHD (e.g., %T^H1/Gui^/Lym H 4.80 ± 3 vs. aGvHD 0.39 ± 0.45). γδ^+^ and Intβ7^+^ T cells were reduced in the Lymphocyte gate yet enriched within T-cell subsets in GvHD (Intβ7^+^T^4+^/Lym in NoGvHD vs. H fold change −0.85; γδ^+^T^4+^/T^3+^ aGvHD 2.87 ± 1.79).

T_regs_ and most cpm-expressing T cells followed the same pattern of Lymphocyte-gate depletion with subset-relative enrichment (T^4+^reg/T^4+^ in NoGvHD vs. H, fold change 1.26). PD-L1^+^T and 2B4^+^T were lowest in Healthy. PD-1^+^, LAG3^+^, 2B4^+^, TIM3^+^, KLRG1^+^, and TIGIT^+^ T-cell subsets were significantly increased in NoGvHD vs. H, as well as in aGvHD and cGvHD vs. H. In the B-cell compartment, B_nai_ dominated in Healthy and NoGvHD; frequencies of B_mem_ were higher in Healthy (B_mem_/Lym). Additionally, compared to NoGvHD, B_mem_ significantly increased in cGvHD (*p* < 0.005), and B_reg_ were reduced in GvHD versus NoGvHD (especially cGvHD vs. NoGvHD, *p* < 0.005) and GvHD versus Healthy (**Figure 1b**; **Supplementary Figure 2b**).

### Innate cells

Innate cells were similarly quantified in two different gates, a Lymphocyte gate and another gate referred to innate subsets (as given in **Supplementary Table 1**).

NK^56+^ subsets were generally expanded in NoGvHD and cGvHD cohorts and reduced in aGvHD. Activation cpm-expressing cells (Tim3^+^ and KLRG1^+^) were increased in NoGvHD, whereas inhibitory cpm-expressing NK^56+^ cells (PD-1^+^, PD-L1^+^, and CTLA-4^+^) were increased in cGvHD and aGvHD (**Figure 1c**; **Supplementary Figure 2c**).

Differentiated NK subsets (NK^56dim16+^ and NK^56dim57-16bri^) were enriched in cGvHD relative to other cohorts, while NK^56bri^ subsets were largely preserved in aGvHD when referenced to NK^56+^. Inhibitory cpm-expressing NK cells (PD-1^+^, PD-L1^+^, and CTLA-4^+^) were increased in cGvHD and aGvHD versus Healthy or NoGvHD (e.g., PD-L1^+^NK^56+^/NK^56+^ H/NoGvHD fold change 24.63, *p* = 0.000089). CIK^56+^ and MAIT cells were highest in Healthy and reduced in GvHD; cpm-expressing CIK^56+^ subsets were lowest in Healthy and increased in GvHD (e.g., PD-L1^+^CIK^56+^/CIK^56+^ H/cGvHD fold change 2.49). iNKT cells remained uniformly low across cohorts; the NK-like iNKT^161+^ subset was enriched in Healthy and aGvHD versus NoGvHD or cGvHD (e.g., iNKT^161+^/6B11 NoGvHD/aGvHD fold change 250.31).

NK regulation was preserved in NoGvHD and progressively dysregulated in GvHD cohorts, while CIK and iNKT compartments shifted toward an inhibitory cpm-expressing phenotype in cGvHD.

**Figure 2** shows examples of selected cell subtypes and the gates used for flow cytometry analyses. In NoGvHD vs. Healthy, we observed decreased Tim3^+^Mo/non Lym and increased B_reg_/B (**Figure 2a**). In aGvHD vs. NoGvHD, we found increased T_pro-late_/Lym and TIGIT^+^T^4−^/T^4−^/Lym, while NK^56dim57+^/Lym were decreased (**Figure 2b**). In cGvHD vs. NoGvHD, B_reg_/B and NK^56dim57+^/Lym (not significant) were decreased (**Figure 2c**). Additional figures are available upon reasonable request.

**Figure 2:**
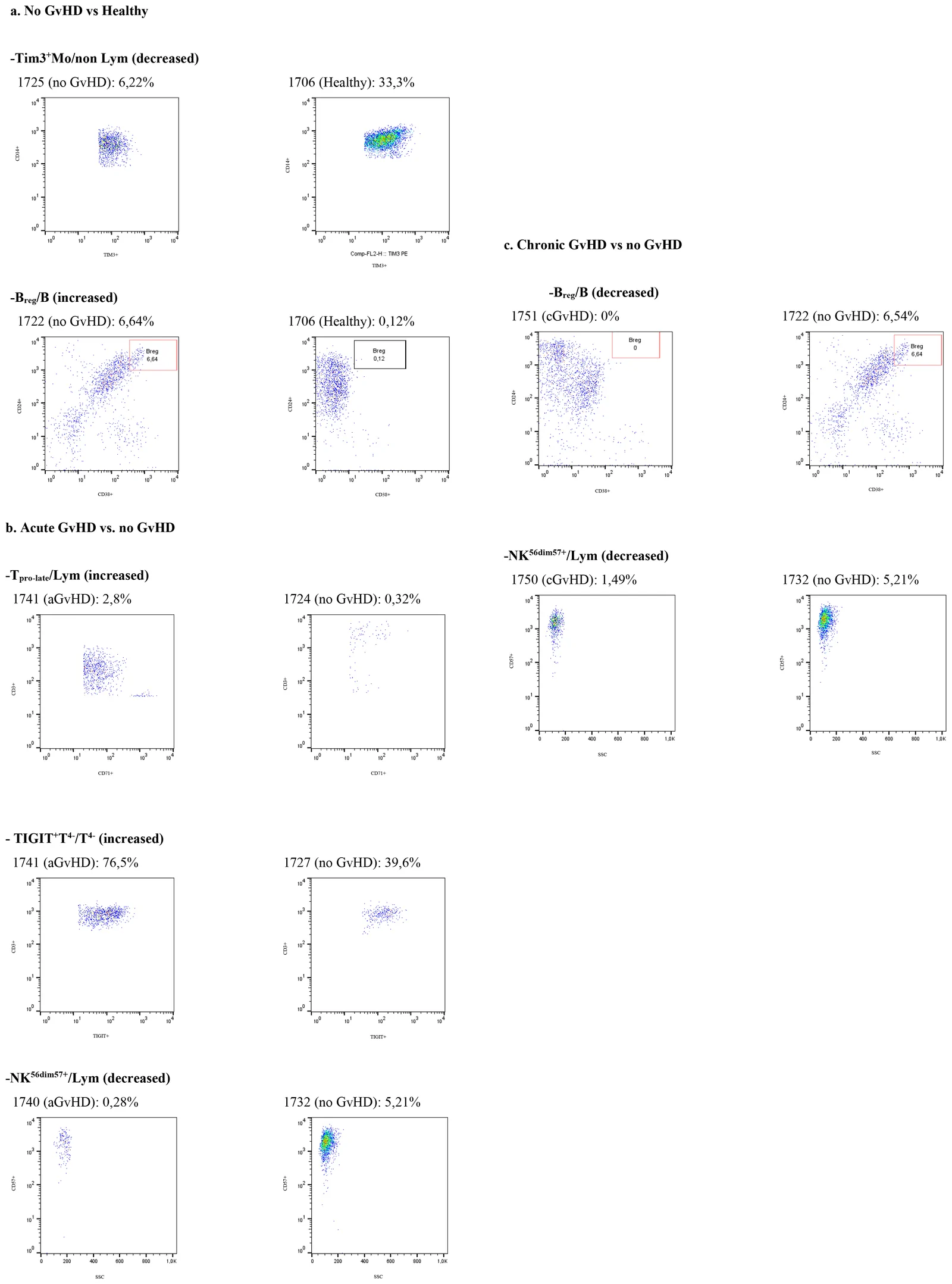
Flow cytometry plots of selected cell subsets and gates. **(a)** No GvHD vs. Healthy, **(b)** Acute GvHD vs. no GvHD, **(c)** Chronic GvHD vs. no GvHD.

### Clinical sub-cohort correlations

We then compared the cellular compositions in patient subgroups sub-classified in clinically defined entities. These included severity of cGvHD, DLI-induced GvHD, overlap a/cGvHD, multi-organ involvement of cGvHD, and clinical onset (*de novo* vs. progressive).

#### Severity of GvHD in patients with cGvHD

We identified patients with either severe (*n* = 10) or mild/moderate (*n* = 8) cGvHD (**Supplementary Tables 9a**; **2**, **Figure 3**).

**Figure 3:**
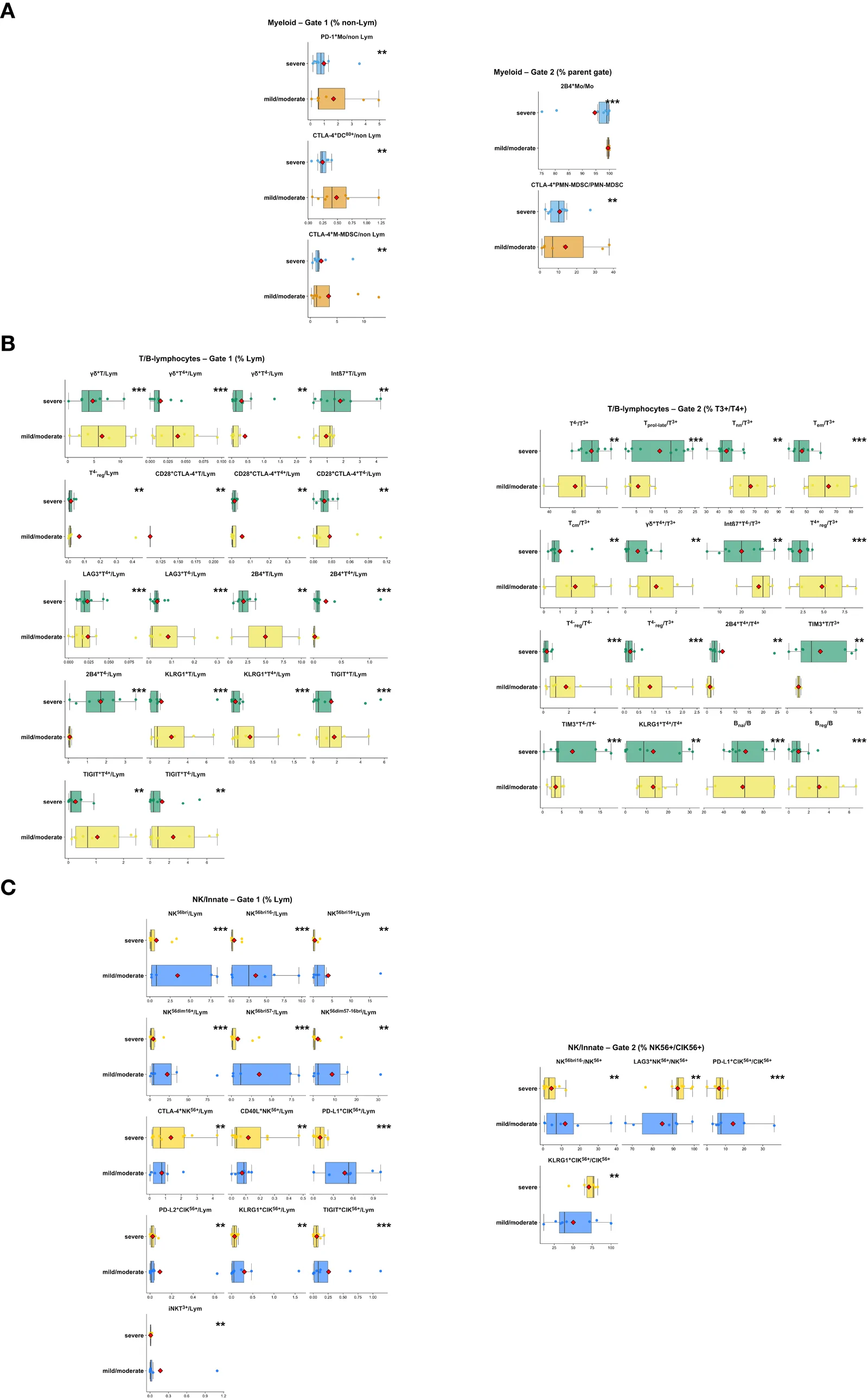
cGvHD severity: severe (*n* = 10) versus mild/moderate (*n* = 8). Comparison of immune cell frequencies in patients with cGvHD stratified by clinical severity. **(a)** Myeloid, **(b)** adaptive, and **(c)** innate cells. Only significant comparisons (*p* ≤ 0.1) are shown. Cell-subset frequencies in severe versus mild/moderate cGvHD, as evaluated by flow cytometry. Tidyplots horizontal-orientation visualization; bars show mean ± SD; significance levels per panel as colour-coded markers (***p ≤ 0.005; **p ≤ 0.05; *p ≤ 0.1). Detailed numerical comparisons are listed in **Supplementary Table 3**. Cell-subset abbreviations are defined in **Supplementary Table 1**.

##### Myeloid compartment

Patients with severe vs. mild/moderate disease showed lower frequencies of regulatory and tolerogenic myeloid subsets: PD-1^+^Mo, DC^80+^, CTLA-4^+^DC^80+^/DC_mat_^80+^, and CTLA-4^+^M-/PMN-MDSC.

##### Adaptive compartment

With respect to adaptive cells, regulatory, anti-tumor and T helper, B- and T-cell subsets were reduced in severe vs. mild/moderate disease (T^H1^, T^H2^, γδ^+^T, T^4+reg^, B_nai_, and B_reg_), coupling myeloid erosion with regulatory T- and B-cell depletion.

##### Innate compartment

With respect to innate cells, NK^56bri^ and NK^56dim^ frequencies were reduced in severe compared to mild/moderate disease, and frequencies of CTLA-4^+^NK^56+^ and CD40L^+^NK^56+^ cells increased, demonstrating regulatory deficits in the adaptive compartment and concurrent exhaustion-marker and activation-marker expression on NK cells.

#### Clinical onset of cGvHD

*De novo*/quiescent disease (*n* = 13) vs. progressive disease (*n* = 5) (**Supplementary Tables 9a**; **3**; **Supplementary Figure 3**).

##### Myeloid compartment

Progressive vs. *de novo*/quiescent cGvHD showed lower myeloid frequencies in CTLA-4^+^M-MDSC and tolerogenic DC subsets, fitting continued tolerance loss rather than a stable post-aGvHD state.

##### Adaptive compartment

Progressive vs. *de novo*/quiescent cGvHD showed increased frequencies of cpm (PD-1^+^T, TIGIT^+^T, LAG3^+^, and 2B4^+^)-expressing and effector-memory T cells.

##### Innate compartment

Progressive vs. *de novo*/quiescent cGvHD was characterized by higher frequencies of NK^56dim16+^ and inhibitory cpm-expressing NK^56+^ cells and lower frequencies of iNKT and MAIT cells.

#### Number of involved organs in patients with cGvHD

We further analyzed patients with cGvHD who had single-organ involvement (*n* = 4) vs. multi-organ involvement (>1 organ, *n* = 12) (**Supplementary Tables 9a**; **4**; **Supplementary Figure 4**).

##### Myeloid compartment

Patients with multi-organ vs. single-organ involvement presented with significantly lower frequencies of PD-1^+^Mo/non Lym and PD-1^+^Mo/Mo. In contrast, higher frequencies of CTLA-4^+^M-MDSC/non Lym and PMN-MDSC/non Lym were observed.

##### Adaptive compartment

Patients with multi-organ vs. single-organ involvement showed increased frequencies of Intβ7^+^T and γδ^+^T^4−^/Lym, but reduced frequencies of 2B4^+^T/Lym, CD28^+^CTLA-4^+^T^4+^/Lym, and regulatory T cells.

##### Innate compartment

Patients with multi-organ vs. single-organ involvement presented with decreased frequencies of NK^56bri/dim^ and increased frequencies of cpm-expressing NK^56+^ cells.

#### DLI-induced vs. non-induced cGvHD

We also observed differences in patients who, prior to GvHD development, had received prophylactic DLIs (DLI-yes, *n* = 4 vs. DLI-no, *n* = 14) (**Supplementary Tables 9a**; **5**, **Figure 4**; **Supplementary Figure 7**).

**Figure 4:**
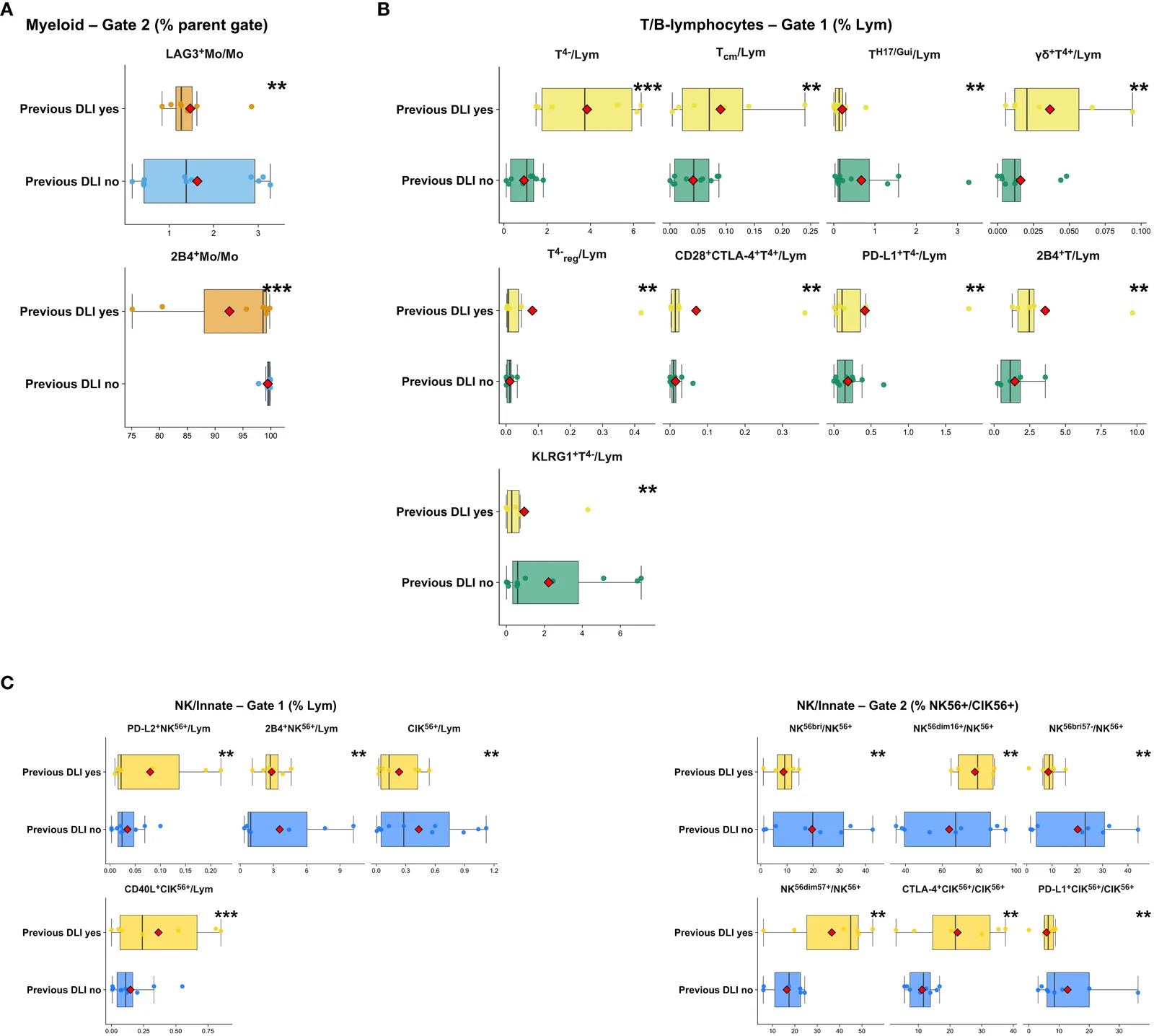
DLI-induced cGvHD versus non-induced cGvHD. Comparison of immune cell frequencies in patients with cGvHD with versus without prior donor lymphocyte infusion (DLI). **(a)** Monocytes, **(b)** adaptive, and **(c)** innate cells. Cell-subset frequencies in DLI-induced versus non-induced cGvHD, as evaluated by flow cytometry. Bars show mean ± SD; significance levels indicated as in **Figure 2**. Detailed numerical comparisons are listed in **Supplementary Table 6**.

##### Myeloid compartment

Patients with DLI-induced cGvHD showed modest changes of the myeloid compartment, with relative preservation of the regulatory monocyte network. Only LAG3^+^Mo and 2B4^+^Mo were decreased in DLI-induced GvHD.

##### Adaptive compartment

The adaptive compartment showed increased T^4−^, T_cm_, γδ^+^T^4+^, T^4−^reg, CD28^+^CTLA-4^+^T^4+^, PD-L1^+^T^4−^, and 2B4^+^T cells and reduced T^H17^ and KLRG1^+^ subsets in DLI-induced vs. non-induced GvHD.

##### Innate compartment

NK^56dim16+^ and CTLA-4^+^CIK^56+^ innate cells were increased in DLI-induced vs. non-induced GvHD.

#### Overlap (a/c) vs. no overlap cGvHD

We also identified patients with a/cGvHD (*n* = 4), which were compared to patients with classic (no overlap) cGvHD (*n* = 14) (**Supplementary Tables 9b**; **6**; **Supplementary Figure 6**).

##### Myeloid compartment

Patients with overlap vs. no overlap cGvHD showed reduced frequencies of PD-1^+^Mo and 2B4^+^Mo cells.

##### Adaptive compartment

Patients with overlap vs. no overlap cGvHD showed higher frequencies of cpm (PD-L1^+^, TIGIT^+^, and CTLA-4^+^ T cells)-expressing T cells alongside with Intβ7^+^ T cells and T_prol-late/_T^3+^, reflecting chronic stimulation accompanied with an aGvHD-like hyperactivation profile.

##### Innate compartment

Patients with overlap vs. no overlap cGvHD showed a profile similar to aGvHD hyperactivation and cGvHD exhaustion, arguing overlap a/cGvHD as a separate biological entity.

#### Development of GvHD in patients initially without GvHD

In further exploratory analyses, we identified patients in our cohort without clinical signs of GvHD at the time of blood analysis, who later on did develop GvHD (*n* = 4) within 6 weeks after the baseline assessment. We compared this group of patients to those who remained without GvHD during a similar or longer period of observation (patients without GvHD, *n* = 15). Importantly, all these patients were off immunosuppression at the time of blood sample acquisition. (**Supplementary Tables 9d**; **7**, **Figure 5**).

**Figure 5:**
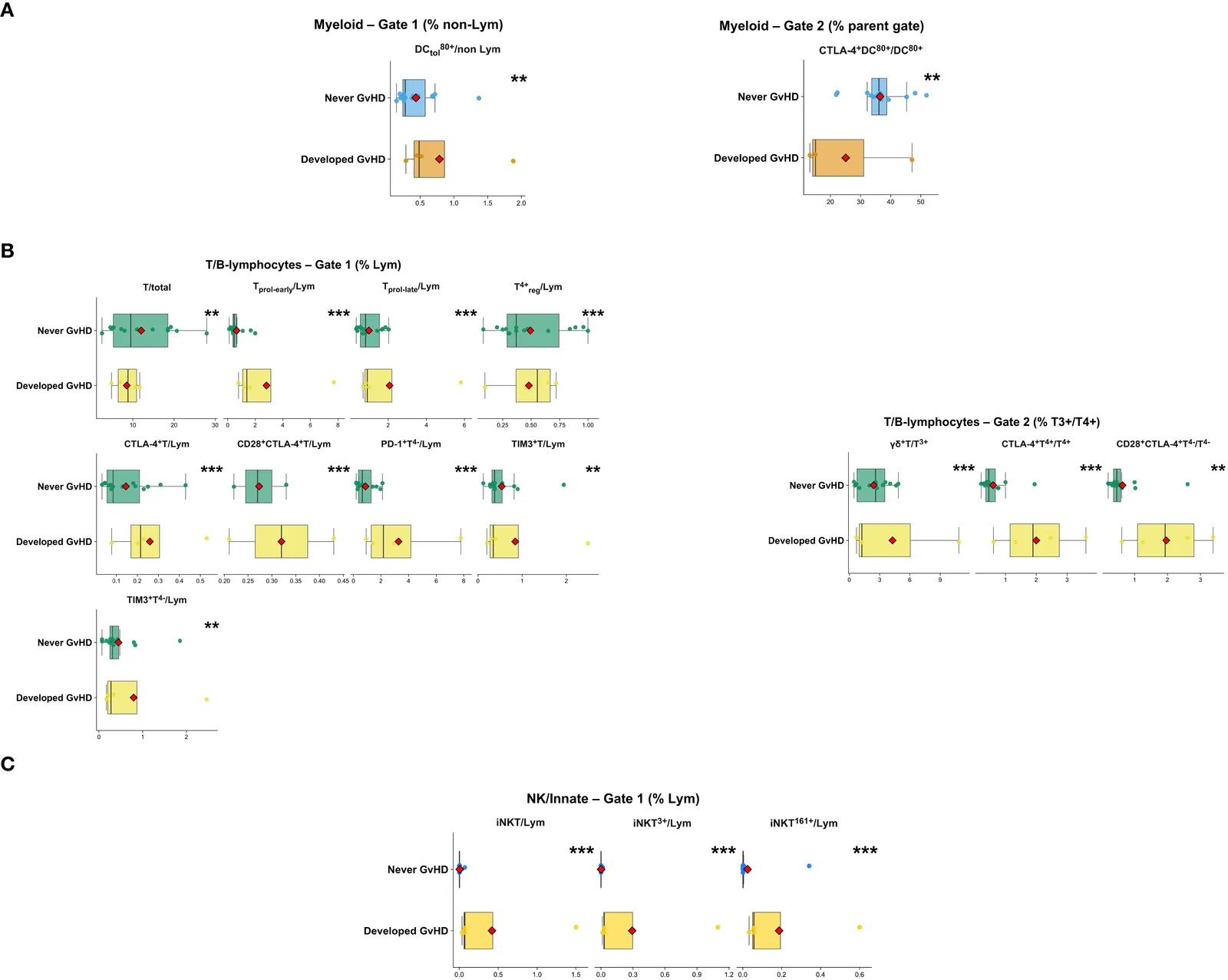
NoGvHD at baseline: subsequent GvHD development versus stable course. Comparison of baseline immune cell frequencies in NoGvHD patients who later developed GvHD (*n* = 4) versus those who remained without GvHD throughout follow-up (*n* = 15). **(a)** Myeloid, **(b)** adaptive, and **(c)** innate cells. Cell-subset frequencies at baseline in NoGvHD patients (*n* = 4 developed GvHD vs. *n* = 14 stable), as evaluated by flow cytometry. Bars show mean ± SD; significance levels as in **Figure 2**. Detailed comparisons are listed in **Supplementary Table 8**.

##### Myeloid compartment

Patients who developed GvHD within 6 weeks showed elevated frequencies of LAG3^+^Mo, DC_tol_^80+^, and PMN-MDSC/Non at baseline compared to those without development of GvHD.

##### Adaptive compartment

Patients who developed GvHD showed elevated early (T_prol-early/_Lym) and late proliferating T cells (T_prol-early_), T_em_, and several cpm-expressing T-cell subsets (CTLA-4^+^T, CD28^+^CTLA-4^+^T, PD-1^+^T, PD-L1^+^T, 2B4^+^T^4+^, and Tim3^+^T) together with reduced T^4+reg^/Lym, compared to the group without development of GvHD.

##### Innate compartment

Patients who developed GvHD within 6 weeks showed higher frequencies of iNKT subsets (iNKT, iNKT^3+^, and iNKT^161+^) and lower frequencies of PD-L2^+^NK^56+^.

#### Development of disease relapse in patients without GvHD

In another exploratory analysis, we describe the composition of immune cell subtypes in patients who were in remission and had no clinical signs of GvHD at the time of the blood sample assessment, but several months after (up to 6 months) developed relapse of their disease (*n* = 2), and compared them to those patients who did not develop a relapse (*n* = 17) during a similar period of observation (**Supplementary Tables 9d**; **8**).

##### Myeloid compartment

Patients who developed a relapse within 6 months showed higher frequencies of LAG3^+^ monocytes (LAG3^+^Mo/non Lym) and CTLA-4^+^PMN-MDSC, whereas most conventional monocyte, dendritic-cell, and MDSC populations remained comparable between groups. Within monocyte subsets, a trend toward increased LAG3^+^Mo/Mo frequencies was observed, suggesting enrichment of inhibitory myeloid phenotypes before clinical relapse.

##### Adaptive compartment

Patients who developed a relapse were characterized by increased frequencies of proliferating T cells (T_prol-early_ and T_prol-late_), regulatory T-cell subsets (T^4−reg^), and multiple cpm-positive T-cell populations, including CTLA-4^+^T, CTLA-4^+^T^4+^, PD-1^+^T^4+^, PD-1^+^T^4−^, LAG3^+^T, and LAG3^+^T^4−^. Increased frequencies of T^H1^ cells, γδ^+^T^4−^ cells, Intβ7^+^-expressing T cells, and PD-L1^+^T^4−^ cells were also observed. In contrast, lower frequencies of naïve T cells (T_nn_) and reduced frequencies of several checkpoint-positive subsets within their parent populations were detected.

##### Innate compartment

Patients who subsequently developed a relapse displayed higher frequencies of MAIT cells and invariant NKT-cell subsets (iNKT, iNKT^3+^, and iNKT^161+^), together with increased NK^56dim57−^ cells and PD-1^+^NK^56+^ cells. In contrast, lower frequencies of CTLA-4^+^CIK^56+^ cells and trends toward reduced mature NK-cell subsets were observed. Overall, relapse development was associated with expansion of innate-like lymphocyte populations and activated NK-cell phenotypes.

## Discussion

Allogeneic HSCT outcomes reflect the complex equilibrium between GvM/GvL activity and GvH immunity (22–24). While limited alloimmune activation (mild-to-moderate GvHD) is manageable, excessive immune activation (severe GvHD) results in tissue injury and increased TRM risk. Despite substantial advances in GvHD prophylaxis and treatment, the mechanisms determining whether immune reconstitution after alloHSCT results in tolerance, effective immune surveillance, or pathological inflammation remain incompletely understood.

In this multicenter collaboration, we applied extensive immune phenotyping of clinically annotated alloHSCT recipients to characterize immune states associated with aGvHD, cGvHD, or the absence of GvHD. Rather than identifying isolated cellular alterations, our findings support a continuum of immune states after transplantation: a distinct post-HSCT immune equilibrium in patients without GvHD (NoGvHD), progressive disruption of regulatory networks in cGvHD, and predominant alloimmune activation in aGvHD. Three concepts emerge from this analysis: first, NoGvHD should not be interpreted as a return to healthy immunity; second, GvHD reflects distinct forms of immune dysregulation; and third, immune profiling may provide a framework for future risk stratification and therapeutic guidance.

### NoGvHD is not “Healthy minus disease”

Patients without GvHD after alloHSCT in our study were clinically asymptomatic, in complete remission, and free of infection. Nevertheless, their immune composition differed substantially from that of age-comparable healthy individuals. Our findings demonstrate that successful transplantation does not restore a pre-transplant immune state, but rather establishes a remodeled immune equilibrium shaped by donor-derived immune reconstitution with persistent antigen exposure and continuous inflammatory pressure (18, 25–28). This distinction is particularly evident in the regulatory compartment. Compared with healthy donors, NoGvHD patients showed reduced frequencies of several tolerogenic myeloid populations, including monocyte subsets, M-MDSCs, and regulatory dendritic-cell populations, while the remaining monocytes displayed increased expression of inhibitory checkpoint molecules (**Figure 1**; **Supplementary Figure 1**). Thus, immune tolerance after alloHSCT is not maintained by restoration of normal cellular composition, but rather by a quantitatively altered and functionally adapted regulatory network. This observation is consistent with previous studies demonstrating that immune reconstitution after alloHSCT occurs through defined temporal trajectories, with recipient immune composition gradually approaching donor-derived patterns only after several months and with distinct intermediate states during recovery (29–35). Similarly, adaptive and innate lymphoid compartments showed profound remodelling in clinically stable NoGvHD patients.

These observations have an important clinical implication: healthy donors are not the appropriate comparator group when interpreting immune-monitoring panels after HSCT.

### What do the cells stand for? Functional reading of the cohort differences

When considered according to biological function rather than individual cell populations, the observed immune differences cluster into distinct regulatory, inflammatory, and dysfunctional effector states (**Figure 1**; **Supplementary Figure 2** for the overall cohort comparison). Across myeloid and lymphoid compartments, regulatory populations were progressively depleted along the spectrum from NoGvHD toward GvHD. These populations, including checkpoint-expressing monocytes, tolerogenic dendritic cells, M-MDSCs, regulatory T cells, and regulatory B-cell subsets, normally contribute to limiting excessive T-cell expansion (36–45). Loss of these regulatory mechanisms may therefore represent a central event in the transition from immune tolerance toward GvHD, which suggests that monitoring its components may identify patients transitioning toward GvHD before clinical onset, providing a potential window for early intervention.

The importance of regulatory recovery is supported by previous studies demonstrating associations between impaired T_reg_ reconstitution and aGvHD risk, as well as an imbalance between regulatory and effector populations in cGvHD (46, 47). Similarly, expansion of pathogenic B-cell populations accompanied by reduced regulatory B-cell activity has been described as a hallmark of cGvHD (48). Together with our findings, these observations suggest that GvHD is not simply caused by increased effector activity, but also by failure of the regulatory networks required to maintain alloimmune tolerance.

### Acute and chronic GvHD represent distinct trajectories of immune dysregulation

Our data suggest that aGvHD and cGvHD represent biologically distinct trajectories rather than different degrees of the same process. aGvHD was characterized by a predominant alloactivation signature, with expansion of proliferative and costimulatory effector T-cell populations accompanied by erosion of regulatory networks. This pattern is consistent with the classical model of aGvHD, in which tissue injury, inflammatory cytokine release, antigen presentation, and donor T-cell activation amplify each other in a self-perpetuating alloimmune response (49–52).

In contrast, cGvHD displayed a different immune landscape characterized by persistent immune stimulation, altered effector differentiation, and accumulation of checkpoint-expressing immune populations. The enrichment of PD-1-, TIGIT-, and KLRG1-expressing T-cell populations, together with a predominance of effector-memory and cytotoxic NK-cell subsets, suggests a state of chronic antigen exposure and dysfunctional immune remodelling. Similar observations have linked cGvHD with persistent T-cell activation, altered immune exhaustion pathways, and impaired immune regulation (53–58). Taken together, aGvHD presents as a hyperactivation profile (alloactivation up, tolerogenic down, and surveillance redistributed), and cGvHD presents as an exhaustion profile (chronic-stimulation cpm-expressing cells elevated, mature cytotoxic NK^56dim^ subsets dominant, and tolerogenic and regulatory networks progressively depleted). The NoGvHD cohort sits functionally between Healthy and cGvHD, with tolerogenic and regulatory networks partly preserved but quantitatively reduced compared with healthy donors.

### Clinical subgroups

Subgroup analyses demonstrated that severe, progressive, multi-organ, and DLI-associated cGvHD were associated with stronger disruption of regulatory networks and increased effector activation (59–63). In severe cGvHD (**Supplementary Table 2**, **Figure 3**), the dominant pattern was regulatory-deficit with reduced PD-1^+^Mo, DC^80+^, CTLA-4^+^DC^80+^/DC_mat_^80+^, CTLA-4^+^M-/PMN-MDSC, T^4+reg^, B_nai_, and B_reg_ while CTLA-4^+^/CD40L^+^NK^56+^ increased. This finding suggests that cGvHD severity is not simply characterized by generalized lymphocyte expansion but strengthens the idea of a broader loss of immune regulation. In multi-organ cGvHD (**Supplementary Table 4**; **Supplementary Figure 4**), increased integrin-β7^+^ T cells and γδ^+^T^4−^ cells are compatible with enhanced tissue-homing/effector activity. In DLI-induced cGvHD (**Supplementary Table 5**, **Figure 3**; **Supplementary Figure 7**), the adaptive compartment showed a distinct activation/regulatory signature with increase in T^4−^, T_cm_, γδ^+^T^4+^, T^4−reg^, CD28^+^CTLA-4^+^T^4+^, PD-L1^+^T^4−^, and 2B4^+^T-cell subsets, accompanied by increased NK^56dim16+^ and CTLA-4^+^CIK^56+^, while myeloid changes were limited to decreased LAG3^+^ and 2B4^+^ monocytes. This phenotype suggests that DLI-associated cGvHD may arise through a different immunological trajectory than non-DLI-associated cGvHD representing a state of recent alloimmune activation accompanied by compensatory regulatory/checkpoint responses, rather than a purely terminal effector phenotype. The concomitant increase in NK^56dim16+^ and CTLA-4^+^CIK^56+^ cells is compatible with recruitment of cytotoxic innate effectors after DLI. In contrast, the relatively limited myeloid changes may indicate that DLI-induced cGvHD is driven less by broad remodeling of the regulatory myeloid compartment than by donor-derived lymphocyte activation.

In overlap a/cGvHD (**Supplementary Table 6**; **Supplementary Figure 5**), chronic T-cell stimulation (PD-L1^+^, TIGIT^+^, and CTLA-4^+^ T), alongside Intβ7^+^ homing and contracted regulatory PD-1^+^Mo and 2B4^+^Mo support its reading as a separate biological entity rather than a “late acute GvHD” (10).

A subgroup of clinically stable NoGvHD patients displayed immune features distinct from the broader NoGvHD population and subsequently developed GvHD within weeks after sampling. This “High-risk” NoGvHD subgroup had altered cpm-expressing T cells, iNKT subsets at baseline, and a contraction of tolerogenic CTLA-4^+^DC^80+^ DCs (**Figure 4**; **Supplementary Table 7**) (36, 64). Although these observations require validation in prospective cohorts, they support the concept that clinical manifestations of GvHD may be preceded by measurable alterations in immune cell subsets (65).

### Potential implications for immune-guided therapeutic strategies

Our findings may have therapeutic implications. If GvHD represents a failure of immune equilibrium, successful treatment may require restoration of regulation rather than nonspecific suppression of immunity. ECP represents one example of such an approach, promoting tolerogenic antigen presentation, reducing pathogenic effector responses, and supporting regulatory immune recovery (66, 67). Similarly, strategies aimed at expanding regulatory T cells, including low-dose IL-2 and adoptive regulatory T-cell approaches, are based on the concept of restoring immune regulation; even CD19-B-cell-directed CART-Cell therapy may be useful in cGvHD where B_mem_ might play a role (68, 69). Conversely, immune states dominated by inflammatory activation or persistent effector dysfunction may be more susceptible to pathway-directed inhibition. The clinical activity of JAK1/2 inhibition and ROCK2 inhibition in GvHD demonstrates that targeting cytokine-driven inflammation, pathogenic T-cell polarization, and fibrotic pathways can restore aspects of immune homeostasis (9, 70, 71). Whether immune phenotypes identified through high-dimensional monitoring may guide selection of immune-directed therapies or serve as pharmacodynamic markers of response remains an important area for future investigation.

## Conclusion, limitations, and future directions

Our study demonstrates that integrated phenotyping across four cohorts (Healthy, NoGvHD, cGvHD, and aGvHD) reveals immunologically distinguishable groups. Immune reconstitution after alloHSCT is characterized by distinct and measurable immune balance rather than a simple return to normal immunity. Patients without GvHD maintain a unique post-transplant immune state in which regulatory networks remain partially preserved, whereas aGvHD and cGvHD represent different trajectories of immune dysregulation characterized by alloimmune hyperactivation and chronic dysfunctional remodelling, respectively. These findings provide a framework for understanding immune heterogeneity after alloHSCT and support future strategies integrating immune monitoring for GvHD prediction and potentially biomarker-based treatment decisions.

We acknowledge some limitations in our study: The design is cross-sectional, the aGvHD cohort is small (*n* = 6), and no longitudinal validation was performed. Sampling time points obviously differed across cohorts (median time from HSCT: NoGvHD 11.4 months, aGvHD 3.2 months, and cGvHD 18.7 months); therefore, part of the between-cohort differences may reflect the stage of immune reconstitution rather than GvHD biology alone. Patients with GvHD were also heterogeneous in disease severity (e.g., severe vs. mild/moderate cGvHD) and in concurrent immunosuppression (corticosteroids, ruxolitinib, and calcineurin inhibitors), each of which can independently shape immune-cell composition and therefore represents a potential confounder. On the other hand, extensive clinical annotation across high-volume, experienced transplant centers enabled detailed characterization of GvHD cohorts and clinically relevant subgroups. Furthermore, consistent and biologically coherent phenotypic patterns emerged across cohorts and sub-cohorts, supporting their robustness, although warranting prospective, longitudinal validation. Future work should integrate single-cell RNA-seq, CyTOF, and spatial transcriptomics to trace these dynamics over time, AI-driven modeling for predictive immune signatures, and interventional studies. The overall aim remains precision immune monitoring that predicts, prevents, and mitigates GvHD while preserving anti-leukemic immunity post-HSCT.

## Data Availability

The data has been uploaded to: https://doi.org/10.5281/zenodo.23188075. Any further queires can be directed to the corresponding author.
