# Supplementary material for: Composition of immune cells in peripheral blood of patients with leukemia in remission after stem cell transplantation (HSCT) with or without graft-versus-host disease (GvHD): a comparative analysis with healthy donors
Source: Front Immunol. 2026 Oct 23;17:1934726. doi: 10.3389/fimmu.2026.1934726 (PMC13740609; doi:10.3389/fimmu.2026.1934726)
Supplement: Supplementary file 1 [file DataSheet1.docx]

1. **Supplementary background: Immune cells and the relevance of immune monitoring**

### **1.1. Myeloid cells**

**1.1.1.	Activated and tolerogenic monocyte subtypes**

In healthy individuals, monocytes occur mainly in a resting state but can differentiate into pro- or anti-inflammatory subsets (Sanberg et al. 2010; Kapellos et al. 2019).

In transplanted patients without GvHD (‘noGvHD’), monocytes often exhibit an anti-inflammatory phenotype that promotes immune tolerance and contributes to tissue protection during reconstitution of the immune system (Tanaka et al. 2022; Lu et al. 2023).

In transplanted patients with GvHD (‘GvHD’), monocytes are generally highly activated and promote inflammation and tissue damage (Melief et al. 2013).

**1.1.2.	Suppressive and checkpoint marker (cpm) expressing monocyte subtypes**

In healthy individuals, monocytes express only low levels of cpm, such as PD-L1, and can change between pro-inflammatory and anti-inflammatory states to modulate immune response (Cheung et al. 2019).

In noGvHD patients, monocytes often express cpm (e.g. PD-L1) and help prevent excessive T cell activation (Sun et al. 2024).

In GvHD patients, monocytes are often highly activated and release pro inflammatory cytokines, thereby amplifying the disease process (Baur et al. 2023; Cao et al. 2023).

The role of defined cpm-expressing monocytes in healthy individuals, noGvHD patients and GvHD patients is unknown.

**1.1.3.	Activated and tolerogenic Dendritic cell (‘DC’) subtypes**

In healthy individuals, DCs are capable of rapid (highly regulated) differentiation into activated subsets to initiate protective immune responses (Tian et al. 2021; Yu et al. 2019).

In noGvHD patients, a balanced pattern of tolerogenic and activated DCs combine immune reconstitution and tolerance (Stenger et al. 2012).

In GvHD patients, DCs are maximally activated, provide costimulatory signals, promote immune cell activation and mediate tissue damage (Yu et al. 2019; Clark and Chakraverty 2002).

**1.1.4.	Suppressive and checkpoint marker expressing DC subtypes**

In healthy individuals, small frequencies of tolerogenic DCs mediate peripheral tolerance (Thomson 2010; Saei and Hadjati 2013).

In noGvHD patients, suppressive and cpm-expressing DCs damp inflammation and support regulatory immune cycles (Domogalla et al. 2017).

In GvHD patients, cpm-expressing or tolerogenic DCs are reduced or functionally impaired, while some of the mature cpm-expressing subsets can promote inflammation (Tian et al. 2021).

The role of defined cpm-expressing DCs in healthy individuals, noGvHD patients and GvHD patients is unknown.

**1.1.5.	Suppressive and cpm-expressing Myeloid derived suppressive cell (‘MDSC’) subtypes**

In healthy individuals, low frequencies of MDSCs with reduced immunosuppressive activity compared to their counterparts in pathological conditions are found (Apodaca et al. 2019; Dross et al. 2017).

In noGvHD patients, both polymorphonuclear (PMN) MDSCs and monocytic(M) MDSCs proliferate, suppress over activation of T cells and thereby promote immune tolerance (Görgün et al. 2013).

In GvHD patients, the immune suppressive function of MDSCs is lost or is modified to a pro-inflammatory condition, resulting in uncontrolled activation of the immune system and tissue damage (Koehn et al. 2015).

The role of defined cpm-expressing MDSCs in healthy individuals, noGvHD patients and GvHD patients is unknown.

### **1.2. Adaptive cells**

**1.2.1.	Activated and differentiated T cell subtypes**

In healthy individuals, CD3^+^ T cells are the major compartment of T cells for adaptive immunity. CD4^+^ T cells and CD8^+^ T cells coordinate immune responses and mediate cytotoxic defense. Naïve T cells are circulating cells that search for new antigens, change to non-naïve and proliferating T cells that coordinate the rapid response to previous encountered threats (Kawabe et al. 2021; Mold et al. 2019).

In noGvHD patients, CD3^+^ T cells reconstitute adaptive immunity without involvement in GvHD, although the T cell repertoire can, at early stages, display decreased diversity due to restricted thymic provision of cells. CD4^+^ T cells recover more slowly compared to CD8^+^ T cells but are crucial for the reconstitution of immune tolerance, particularly by regulatory T cell subsets. CD8^+^ T cells expand more rapidly, support anti-infection protection, and GvL functions. Non-naïve and proliferating T cells have an initial immune protective function, while naïve T cells are reconstituted only slowly due to thymus dependency. Reconstitution is modulated and controlled by immunosuppression (Ma et al. 2025; Bejanyan et al. 2018).

In GvHD patients, CD3^+^ T cells infiltrate target organs and are the main effectors of alloimmune pathology. CD4^+^ T cells produce inflammatory mediators, which promote disease, while CD8^+^ T

cells exert direct cytotoxic effects leading to host tissue damage. Non-naïve and proliferating T cells exhibit hyperactivation and proinflammatory bias, facilitating disease development (Wang et al. 2024; Ranti et al. 2018).

**1.2.2.	Memory T cells**

In healthy individuals, memory T cells (effector memory, T_em_ and central memory, T_cm_) mediate rapid, antigen specific recall responses against infections or tumor cells (Farber et al. 2014).

In noGvHD patients, memory T cells preserve immune defense and anti-leukemic activity. Subsets can also acquire regulatory roles to limit host damage (Jiang et al. 2021).

In GvHD patients, activated memory T cells, especially T_em_, show increased inflammatory activity against GvHD pathology (Jiang et al. 2021).

**1.2.3.	Activated T cells**

In healthy individuals, activated T cells express costimulatory and activation markers such as CD28 and CD40L, mediating cell proliferation, DC-activation, cytokine release, cytotoxic activity, B cell help (Acuto and Michel 2003; van Kooten and Banchereau 2000).

In noGvHD patients, activated T cells have a critical role in immune reconstitution. They protect against infections, mediate GvL effects and immune tolerance (Dekker et al. 2020).

In GvHD patients, activated T cells, including CD4^+^CD28^+^, CD8^+^CD28^+^ and CD40L^+^ subsets, are major effectors and contributors to disease pathology. They migrate into tissues and boost inflammatory cytokine production. CD28 and CD40L are signaling exhibits long term effects, perpetuating augmented alloreactive T cell expansion and tissue damage, which induces further progression of disease (Zeiser and Blazar 2017; Sakoda et al. 2007).

**1.2.4.	T Helper cells (‘TH’)**

In healthy individuals, functional TH subsets balance pro- and anti-inflammatory responses, maintaining immune homeostasis (e.g. TH^1^, TH^2^, TH^17^) (Ogonek et al. 2016; Koning et al. 2021).

In noGvHD patients, TH gradually reconstitute and support adaptive immunity and the coordination of B cell and cytotoxic T cell responses (Ogonek et al. 2016; Koning et al. 2021).

In GvHD patients, dysregulated TH cell compositions (e.g. of TH^1^, TH^2^, TH^17^) can contribute to inflammation and tissue damage (Alho et al. 2016; Teshima et al. 2023). Two different gating strategies for TH cell subsets (e.g. TH^1^, TH^2^, TH^17^) are described (Maecker et al. 2012; Mousset et al. 2019).

**1.2.5.	Anti-tumor directed γδ^+^ T cells**

In healthy individuals, γδ T cells are involved in immune surveillance by recognizing stress induced ligands on hematopoietic cells, leading to tumor cell kill through direct cytotoxicity (Vantourout and Hayday 2013; Hannani et al. 2012).

In noGvHD patients, γδ T cells typically adopt a regulatory or tissue protective phenotype that favors immune reconstitution and supports graft tolerance with residual GvL activity (Ye et al. 2020).

In GvHD patients, γδ T cells are typically hyperactivated, produce inflammatory cytokines and contribute to tissue damage, but also to GvL responses, demonstrating the balance between protection and pathology (Barros et al. 2021; Fang et al. 2023).

**1.2.6.	Anti-tumor directed Integrin ß7^+^ T cells**

In healthy individuals, Integrin ß7^+^ (‘Intß7’) T cells (CD3^+^Intß7^+^, CD4^+^Intß7^+^, CD8^+^Intß7^+^) contribute tissue surveillance and homeostasis. Stimulation by dendritic cells increase the frequencies of Intß7^+^ expressing T cells, which is associated with cytotoxic immune function and contributes to immune control (Rackl et al. 2022)

In noGvHD patients, Intß7^+^ T cells promote immune reconstitution and tissue repair. They can help maintaining tolerance at epithelial interfaces, limiting excessive immune activation and are responsible for anti-leukemic processes (Rejeski et al. 2024; Rackl et al. 2022).

In GvHD patients, Intß7^+^ T cells are often highly activated, migrate into target tissues and can drive inflammation and epithelial damage, but can also acquire regulatory or exhausted phenotypes, depending on microenvironmental signals (Rackl et al. 2022).

The exact role of defined Intß7^+^ T cell subtypes in healthy individuals, no GvHD patients, and GvHD patients is not fully resolved.

1.2.7. **Suppressive and checkpoint marker expressing T cells**

In healthy individuals, low levels of cpm-expressing T cells (such as PD-1, CTLA-4 or Tim3) regulate immune responses (Parry et al. 2005; Szabo et al. 2019; Sousa Linhares et al. 2018).

In noGvHD patients, cpm-expressing T cells promote tolerance and prevent excessive alloreactivity (Parry et al. 2005; Gautron et al. 2014; Sousa Linhares et al. 2018).

In GvHD patients, T cells are highly activated and drive inflammation or gain an “exhausted” phenotype with high cpm frequencies, which limits their cytokine production and proliferation (Jiang et al. 2015; Parry et al. 2005; Sousa Linhares et al. 2018).

The role of defined cpm-expressing T cells in healthy individuals, noGvHD patients and GvHD patients is unknown.

**1.2.8.	Regulatory T cells (T_regs_)**

In healthy individuals, CD127^+^ T cells represent mature conventional T cells, while T_regs_ are identified by low or absent CD127 frequencies, which correlates with strong suppressive function (Zhou et al. 2020b; Liu et al. 2006).

In noGvHD patients, T_regs_ maintain immune tolerance and prevent excessive alloreactivity after transplantation (Liu et al. 2006; Sumii et al. 2023).

In GvHD patients, T_regs_ can be reduced or functionally impaired, while CD127^+^ T cells become dominant, driving inflammation and loss of immune regulation (Magenau et al. 2010; Liu et al. 2006).

The specific roles of CD127^+^ T cells and T_regs_ in healthy individuals, noGvHD patients and GvHD patients is not fully defined.

**1.2.9.	Activated and differentiated B cell subtypes**

In healthy individuals, balanced frequencies of B cell subtypes, such as naïve, memory and regulatory B cells modulate immune homeostasis and immune responses (Catalán et al. 2021; Ahsan et al. 2024; Chekol Abebe et al. 2021).

In noGvHD patients, regulatory and memory B cells prevent alloreactive responses, enable B cell recovery and immune modulation (Liao et al. 2025; Chekol Abebe et al. 2021).

In GvHD patients, B cells are often dysregulated with increased activation and antibody production contributing to inflammation and potentially impaired regulatory B cell function reducing capacity to modulate immune responses and promoting disease progression (Agbogan et al. 2022; McManigle et al. 2019).

### **1.3. Innate cells**

**1.3.1.	Activated and differentiated natural killer (‘NK’) subtypes**

NK cells are among the earlies lymphocytes to reconstitute after allogeneic HSCT and play a dual role in GvL effects and GvHD regulation (Ruggeri et al. 2002; Cooper et al. 2001a; Cooper et al. 2001b). The conventional dichotomy of CD56^bright^ immunoregulatory versus CD56^dim^ cytotoxic NK cells was extended by further dissecting subsets along the CD57/CD16 differentiation axis.

In healthy individuals, CD56^bright^ and CD56^dim^ CD57^-^ cells represent immature NK populations with high proliferative and cytokine producing capacity, maintaining immune homeostasis. Terminally differentiated CD56^dim^ CD57^+^ CD16^+^ NK cells display strong cytotoxic potential, including ADCC, while their proliferative responses to cytokines are limited (Björkström et al. 2010; Nielsen et al. 2013).

In noGvHD patients, a similar pattern of NK subsets recovery as in healthy individuals is observed, with balanced frequencies of immature and mature NK cells supporting tolerance and immune reconstitution (Ullrich et al. 2016; Ullah et al. 2016).

In GvHD patients, this balance is disturbed: Immature regulatory NK cells are reduced or delayed in recovery, while CD57^+^ terminally differentiated NK cells are expanded, reflecting persistent immune activation and impaired immune regulation (Ullrich et al. 2016; Nielsen et al. 2013).

**1.3.2.	Suppressive and checkpoint marker expressing NK cells**

In healthy individuals, NK cells are primarily cytotoxic, eliminate virally infected and malignant cells, and show low baseline frequencies of inhibitory cpms such as PD-1, TIGIT or Tim3 (Cao et al. 2020; Davis et al. 2021; Khan et al. 2020).

In noGvHD patients, upregulated cpm on NK cells lower the risk of tissue damage but also weaken their GvL activity (Simonetta et al. 2017; Foley et al. 2011; Davis et al. 2021).

In GvHD patients, NK cells often exhibit functional exhaustion. They express higher levels of inhibitory molecules and display reduced cytotoxicity (Simonetta et al. 2017; Davis et al. 2021; Nihtilä et al. 2024; Cao et al. 2020).

The role of defined cpm-expressing NK cells in healthy individuals, noGvHD patients and GvHD patients is unknown.

**1.3.3.	Activated differentiated cytokine induced killer (‘CIK’) subtypes**

In healthy individuals, CIK cells are generally quiescent but possess the capability to differentiate quickly into functional subsets that possess inflammatory or regulatory functions, thus making them capable of controlling immune responses effectively (Kohrt et al. 2010; Mavers et al. 2017).

In noGvHD patients, CIK cells often gain a regulatory phenotype maintaining immune tolerance and preventing tissue injury during immune reconstitution (Guan et al. 2016; Kohrt et al. 2010).

In GvHD patients, CIK cells are highly activated and enhance inflammation (Erkers et al. 2020; Hirai et al. 2021).

**1.3.4.	Suppressive and cpm-expressing CIK cells**

In healthy individuals, CIK cells express low levels of cpm such as PD-1 thereby maintain immune homeostasis and surveillance without excessive immune suppression (Godfrey et al. 2015; Shimizu et al. 2020).

In noGvHD patients, CIK cells upregulate cpm (e.g. PD-1 and CTLA-4), thereby gaining an immunosuppressive effect of T cell supporting and prevention of immune mediated tissue damage (Blazar et al. 2018; Chakrabarti and Jaiswal 2023). In GvHD patients, some subsets of CIK cells upregulate cpm such as PD-1 and CTLA-4 (Hossain et al. 2017; Nelson et al. 2021).

The role of defined cpm-expressing CIK cells in healthy individuals, noGvHD patients and GvHD patients is unknown.

**1.3.5.	Activated and differentiated invariant natural killer T cells (‘iNKT’) subtypes**

In healthy individuals, iNKT cells are responsible for immune balance by rapidly producing cytokines and controlling other immune cells (Leadbetter and Karlsson 2021; Bharadwaj and Gumperz 2022; Krovi and Gapin 2018).

In noGvHD patients, iNKT cells inhibit alloreactive T cells and promote regulatory pathways thereby preventing the development of GvHD (Krovi and Gapin 2018; Boonchalermvichian et al. 2024).

In GvHD patients, iNKT cells are often downregulated and/or dysfunctional, resulting in the failure of immunoregulatory functions and increased inflammation to drive GvHD pathology (Schmid et al. 2022b).

## 2. Supplemental methods

## Figure preparation

All sub-cohort comparison figures (**Figures 1–4** and **Supplementary Figures 2–6**) were rendered with the tidyplots R package (Engler 2025) from the curated cell-frequency table. Each figure pairs two horizontally arranged panels, Gate 1 (frequencies referenced to the lymphocyte or non-lymphocyte gate) and Gate 2 (frequencies referenced to the corresponding cell subset, e.g. /Mo, /T^3+^, /T^4+^, /T^4-^, /B, /NK^56+^, /CIK^56+^). The order of cell subsets within every panel was forced to match the canonical row order of **Supplementary Table 1** (a/b/c) so that myeloid, adaptive, and innate populations appear identically across the manuscript text, the **supplementary tables**, and every panel of every figure. Only comparisons reaching at least p<0.05 (highly significant or significant; borderline-only effects excluded) are displayed; the corresponding star annotations (***, **) are taken from the **Supplementary Tables 2–9**. Box plots show median and interquartile range; jittered points show individual values; the diamond marker indicates the group mean. Frequencies of basic cell compositions across all four cohorts are summarized in **Figure 1** and **Supplementary Figure 1**.

3. **Supplementary Figures**

*Supplementary figures: four-cohort overview (fold changes and absolute frequencies; Supplementary Figures 1 and 2) and extended frequency comparisons across clinical sub-cohorts (Supplementary Figures 3-7). Plots in Supplementary Figures 1 and 2 generated in GraphPad Prism (provided by V.M.S.); plots in Supplementary Figures 3-7 generated with the tidyplots R package (Engler 2025).*

# **Supplementary Figure 1 — Frequencies of cell subsets across cohorts**

*Mean ± SD frequencies of myeloid (a), adaptive (b), and innate (c) cells, referenced to non-Lym or Lym (left) or to specific subsets (right), in Healthy (n=10), NoGvHD (n=19), cGvHD (n=18), and aGvHD (n=6).*

**a) Myeloid cells**

**b) Adaptive cells**

**c) Innate cells**

*Supplementary Figure 1. Raw frequencies underlying Supplementary Figure 2, as evaluated by flow cytometry. Group comparisons were pairwise; p values are reported in Supplementary Figure 1. Cell-subset abbreviations are listed in Supplementary Table 1*

**Supplementary figure 2**

**a)** **Myeloid cells**

***

***

***

**Fold change**

**Fold change**

***

***

***

***

***

***

**

***

***

***

***

**

**b) Adaptive cells**

**

**

***

***

***

***

**

***

**Fold change**

**Fold change**

***

***

***

***

***

***

***

***

***

**

**

**

**

***

***

**

***

***

***

***

***

***

***

***

***

***

***

***

***

***

***

***

**

**

**

**

***

***

***

***

**

**

***

***

**

***

***

***

***

***

***

***

***

***

***

***

***

***

***

***

**Fold change**

**Fold change**

***

***

***

***

***

***

**

***

***

**

***

**

***

***

**

*

***

***

***

***

***

***

***

***

***

***

***

***

**

***

***

**

**

**

**

**

**

**

**

***

***

***

***

***

***

***

***

***

***

***

***

***

***

***

***

***

***

***

***

**

**

**

***

***

***

***

***

***

***

***

***

***

***

**c) Innate cells**

***

***

***

***

***

***

***

***

**Fold change**

**Fold change**

***

***

***

***

***

***

***

***

***

***

***

***

***

***

***

***

***

***

***

***

***

***

***

***

***

***

**

***

**

***

***

***

***

***

***

***

***

***

**

***

**

***

***

*Supplementary Figure 2. Fold changes of frequencies of myeloid (a), adaptive (b), and innate (c) cells across cohorts, as evaluated by flow cytometry. Statistical analyses used the unpaired t-test. ***: p≤0.005; **: p≤0.05; *: p≤0.1; n.s.: p>0.1. Cell-subset abbreviations are listed in Supplementary Table 1.*

# **Supplementary Figure 3 — cGvHD: de novo or quiescent versus progressive**

*Cell-subset comparison between de novo / quiescent and progressive cGvHD. Panels: (a) myeloid, (b) adaptive, (c) innate cells.*

**(a) Myeloid cells**

**(b) Adaptive cells**

**(c) Innate cells**

*Supplementary Figure 3. Cell-subset frequencies in de novo / quiescent versus progressive cGvHD, as evaluated by flow cytometry. Detailed comparisons are listed in Supplementary Table 4.*

# **Supplementary Figure 4— cGvHD: single-organ versus multi-organ involvement**

*Cell-subset comparison between cGvHD patients with single-organ versus multi-organ involvement. Panels: (a) myeloid, (b) adaptive, (c) innate cells.*

**(a) Myeloid cells**

**(b) Adaptive cells**

**(c) Innate cells**

*Supplementary Figure 4. Cell-subset frequencies in single-organ versus multi-organ cGvHD, as evaluated by flow cytometry. Detailed comparisons are listed in Supplementary Table 5.*

# **Supplementary Figure 6 — Overlap a/cGvHD versus classic cGvHD**

*Cell-subset comparison between overlap a/cGvHD and classic cGvHD patients. Panels: (a) myeloid, (b) adaptive cells; no significant innate-cell differences were detected at p≤0.1.*

**(a) Myeloid cells**

**(b) Adaptive cells**

*Supplementary Figure 6. Cell-subset frequencies in overlap a/cGvHD versus classic cGvHD, as evaluated by flow cytometry. Detailed comparisons are listed in Supplementary Table 7.*

# **Supplementary Figure 7— DLI-induced cGvHD versus non-induced cGvHD (extended panels)**

*Extended cell-subset comparison between DLI-induced and non-induced cGvHD; same data as Figure 2 of the main paper, shown here in supplementary detail. Panels: (a) myeloid, (b) adaptive, (c) innate cells.*

**(a) Myeloid cells**

**(b) Adaptive cells**

**(c) Innate cells**

*Supplementary Figure 7. Cell-subset frequencies in DLI-induced versus non-induced cGvHD (extended panel set; corresponds to Figure 2 of the main paper), as evaluated by flow cytometry. Detailed comparisons are listed in Supplementary Table 6.*

5. **Supplementary tables:**

**Supplementary Table 1: Cellular Subtype Characteristics**

Supp. Table 1a: Myeloid cells

| **I. Monocytes (n=7)** | | | | | | |
| --- | --- | --- | --- | --- | --- | --- |
|  | Name of Subgroups | Abbreviation of Subgroup | Surface Marker | /Cells | /Subtypes | Reference |
| Activated and differentiated monocyte subtypes | | | | | | |
|  | Monocytes | Mo | CD14+ | non Lym | - | (Li et al. 2024a) |
| Suppressive and checkpoint marker expressing monocyte subtypes | | | | | | |
|  | CTLA-4^+^ Monocytes | CTLA-4^+^Mo | CD14+CD152+ | non Lym | Mo | - |
|  | PD-1^+^ Monocytes | PD-1^+^Mo | CD14+CD279+ | non Lym | Mo | (Vago and Gojo 2020) |
|  | PD-L1^+^ Monocytes | PD-L1^+^Mo | CD14+CD274+ | non Lym | Mo | (Ando et al. 2021) |
|  | LAG3^+^ Monocytes | LAG3^+^Mo | CD14+LAG3+ | non Lym | Mo | (Workman et al. 2009) |
|  | 2B4^+^ Monocytes | 2B4^+^Mo | CD14+2B4+ | non Lym | Mo | (Kim et al. 2024) |
|  | Tim3^+^ Monocytes | Tim3^+^Mo | CD14+TIM-3+ | non Lym | Mo | (Ocaña-Guzman et al. 2016) |
| **II. Dendritic cells (n=9)** | | | | | | |
| Activated and differentiated DC subtypes | | | | | | |
|  | DC | DC^80+^ | CD80+ | non Lym | - | (Stenger et al. 2012) |
|  | DC | DC^206+^ | CD206+ | non Lym | - | (Jardine et al. 2020) |
|  | CD80^+^ Mature DC | DC_mat_^80+^ | CD80+CD197+ | non Lym | DC^80+^ | (Yu et al. 2019) |
|  | CD206^+^ Mature DC | DC_mat_^206+^ | CD206+CD197+ | non Lym | DC^206+^ | (Yu et al. 2019) |
| Suppressive and checkpoint marker expressing DC subtypes | | | | | | |
|  | Tolerogenic CD80^+^ DC | DC_tol_^80+^ | CD80+CD85K+ | non Lym | DC^80+^ | (Scroggins et al. 2013) |
|  | Tolerogenic CD206^+^ DC | DC_tol_^206+^ | CD206+CD85K+ | non Lym | DC^206+^ | (Scroggins et al. 2013) |
|  | CTLA-4^+^ DC | CTLA-4^+^DC^80+^ | CD80+CD152+ | non Lym | DC^80+^ | (Sato et al. 2003) |
|  | CTLA-4^+^ DC | CTLA-4^+^DC^206+^ | CD206+CD152+ | non Lym | DC^206+^ | (Sato et al. 2003) |
|  | CTLA-4^+^ CD80^+^ Mature DC | CTLA-4^+^DC_mat_^80+^ | CD80+CD197+CD152+ | non Lym | DC_mat_^80+^ | - |
|  | CTLA-4^+^ CD206^+^ Mature DC | CTLA-4^+^DC_mat_^206+^ | CD206+CD197+CD152+ | non Lym | DC_mat_^206+^ | - |
| **III. Myeloid derived suppressive cells (n=4)** | | | | | | |
| Suppressive and checkpoint marker expressing MDSC subtypes | | | | | | |
|  | PMN-MDSC | PMN-MDSC | CD11b+CD33+CD14- | non Lym | CD11b+CD33^+^ | (Demosthenous et al. 2021) |
|  | M-MDSC | M-MDSC | CD11b+CD33+CD14+ | non Lym | CD11b+CD33^+^ | (Wang et al. 2013) |
|  | CTLA-4^+^ PMN-MDSC | CTLA-4^+^PMN-MDSC | CD11b+CD33+CD14-CD152+ | non Lym | PMN-MDSC | (Hess et al. 2023) |
|  | CTLA-4^+^ M-MDSC | CTLA-4^+^-MDSC | CD11b+CD33+CD14+CD152+ | non Lym | PMN-MDSC | - |

Supp. Table 1b: Adaptive cells

| **I. Activated and differentiated T cell subtypes** | | | | | | | |
| --- | --- | --- | --- | --- | --- | --- | --- |
| Groups | Name of Subgroups | | Abbreviation of Subgroup | Surface Marker | /Cells | /Subtypes | Reference |
| T Cells | | | | | | | |
| T Cells | CD3^+^ T cells | T | | CD3+ | total | - | (Schutti et al. 2024; Maecker et al. 2012) |
|  | CD4^+^ T cells | T^4+^ | | CD3+CD4+ | Lym | T^3+^ | (Schutti et al. 2024; Maecker et al. 2012) |
|  | CD8^+^ T cells | T^4-^ | | CD3+CD4- | Lym | T^3+^ | (Schutti et al. 2024; Maecker et al. 2012) |
| Proliferating T cells | Early CD69^+^ Proliferating T cells | T_prol-early_ | | CD3+CD69+ | Lym | T^3+^ | (Weiner et al. 2021) |
|  | Late CD71^+^ Proliferating T cells | T_prol-late_ | | CD3+CD71+ | Lym | T^3+^ | (Verma et al. 2024) |
| Non-naive T cells | Non-naive T cells | T_nn_ | | CD3+CD45RO+ | Lym | T^3+^ | (Zhang et al. 2005) |
| Naive T cells | Naive T | T_nai_ | | CD3+CD45RO- | Lym | T^4+^ | (Loschi et al. 2015) |
| Memory T cells | Effector (memory) T cells | T_em_ | | CD3+CD45RO+CD197- | Lym | T^3-^ | (Zhang et al. 2005) |
|  | Central (memory) T cells | T_cm_ | | CD3+CD45RO+CD197+ | Lym | T^3+^ | (Soares et al. 2019) |
| Activated T cells | CD28^+^ T cells | CD28^+^T | | CD3+CD28+ | Lym | T^4+^ | (Sakamoto et al. 2022) |
|  | CD4^+^CD28^+^ T cells | CD28^+^T^4+^ | | CD3+CD4+CD28+ | Lym | T^4-^ | (Sakamoto et al. 2022) |
|  | CD8^+^CD28^+^ T cells | CD28^+^T^4-^ | | CD3+CD4-CD28+ | Lym | T^3+^ | (Sakamoto et al. 2022) |
|  | CD40L^+^T cells | CD40L^+^T | | CD3+CD154+ | Lym | T^4+^ | (Carbone et al. 1995) |
|  | CD4^+^CD40L^+^ T cells | CD40L^+^T^4+^ | | CD3+CD4+CD154+ | Lym | T^4-^ | (Carbone et al. 1995) |
|  | CD8^+^CD40L^+^ T cells | CD40L^+^T^4-^ | | CD3+CD4-CD154+ | Lym | T^3+^ | (Tay et al. 2017; Schiele et al. 2025) |
| T helper cells strategy 1 | T helper 1 cells | T^H1/Na^ | | CD4+CD183+ CD196- | Lym | T^4+^ | (Maecker et al. 2012) |
|  | T helper 2 cells | T^H2/Na^ | | CD4+ CD183-CD196- | Lym | T^4+^ | (Maecker et al. 2012) |
|  | T helper 9 cells | T^H9/Na^ | | CD4+ CD194-CD196+ | Lym | T^4+^ | (Maecker et al. 2012) |
|  | T helper 17 cells | T^H17/Na^ | | CD4+ CD183-CD196+ | Lym | T^4+^ | (Maecker et al. 2012) |
| T helper cells strategy 2 | T helper 1 cells | T^H1/Gui^ | | CD4+CD183+CD194-CD196- | Lym | T^4+^ | (Mousset et al. 2019) |
|  | T helper 2 cells | T^H2/Gui^ | | CD4+CD183-CD194+CD196- | Lym | T^4+^ | (Mousset et al. 2019) |
|  | T helper 17 cells | T^H17/Gui^ | | CD4+CD183-CD194+CD196+ | Lym | T^4+^ | (Mousset et al. 2019) |
| Anti-tumor directed T cells | γδ T cells | γδ^+^T | | CD3+TCRγδ+ | Lym | T^3+^ | (Rackl et al. 2024; Rackl et al. 2022) |
|  | CD4^+^ γδ T cells | γδ^+^T^4+^ | | CD3+CD4+TCRγδ+ | Lym | T^4+^ | (Rackl et al. 2024; Rackl et al. 2022) |
|  | CD8^+^ γδ T cells | γδ^+^T^4-^ | | CD3+CD4-TCRγδ+ | Lym | T^4-^ | (Rackl et al. 2024; Rackl et al. 2022) |
|  | Integrin β7 T cells | Intβ7^+^T | | CD3+Intβ7+ | Lym | T^3+^ | (Rackl et al. 2024; Rackl et al. 2022) |
|  | CD4^+^ Intβ7 T cells | Intβ7^+^T^4+^ | | CD3+CD4+Intβ7+ | Lym | T^4+^ | (Rackl et al. 2024; Rackl et al. 2022) |
|  | CD8^+^ Intβ7 T cells | Intβ7^+^T^4-^ | | CD3+CD4-Intβ7+ | Lym | T^4-^ | (Rackl et al. 2024; Rackl et al. 2022) |
| Suppressive and Checkpoint marker expressing T cells | | | | | | | |
| CD127+ T | CD127^+^ T | CD127^+^T | | CD3+CD127+ | Lym | T^3+^ | (Alpdogan et al. 2003) |
| T_regs_ | CD4^+^ Regulatory T cells | T^4+^_reg_ | | CD3+CD4+CD25++CD127(low/-) | Lym | T^4+^ | (Danby et al. 2016) |
|  | CD8^+^ Regulatory T cells | T^4-^_reg_ | | CD3+CD4-CD25++CD127(low/-) | Lym | T^4-^ | (Pepeldjiyska et al. 2022; Schick et al. 2013) |
| CTLA-4^+^ T cells | CTLA-4^+^ T cells | CTLA-4^+^T | | CD3+CD152+ | Lym | T^3+^ | (El Dosoky et al. 2024; Do et al. 2019) |
|  | CD4^+^ CTLA-4^+^ T cells | CTLA-4^+^T^4+^ | | CD3+CD4+CD152+ | Lym | T^4+^ | (El Dosoky et al. 2024; Do et al. 2019) |
|  | CD8^+^ CTLA-4^+^ T cells | CTLA-4^+^T^4-^ | | CD3+CD4-CD152+ | Lym | T^4-^ | (El Dosoky et al. 2024; Do et al. 2019) |
|  | CD28^+^ CTLA-4^+^ T cells | CD28^+^CTLA-4^+^T | | CD3+CD28+CD152+ | Lym | T^3+^ | (Oyewole-Said et al. 2020; Yoo et al. 2016) |
|  | CD4^+^ CD28^+^ CTLA-4^+^ T cells | CD28^+^CTLA-4^+^T^4+^ | | CD3+CD4+CD28+CD152+ | Lym | T^4+^ | (Oyewole-Said et al. 2020; Yoo et al. 2016) |
|  | CD8^+^CD28^+^ CTLA-4^+^ T cells | CD28^+^CTLA-4^+^T^4-^ | | CD3+CD4-CD28+CD152+ | Lym | T^4-^ | (Oyewole-Said et al. 2020; Yoo et al. 2016) |
| PD1^+^ T cells | PD1^+^ T cells | PD-1^+^T | | CD3+CD279+ | Lym | T^3+^ | (Simonetta et al. 2019) |
|  | CD4^+^ PD1^+^ T cells | PD-1^+^T^4+^ | | CD3+CD4+CD279+ | Lym | T^4+^ | (Hossain et al. 2017) |
|  | CD8^+^ PD1^+^ T cells | PD-1^+^T^4-^ | | CD3+CD4-CD279+ | Lym | T^4-^ | (Simonetta et al. 2019) |
| PD-L1^+^ T cells | PD-L1^+^ T cells | PD-L1^+^T | | CD3+CD274+ | Lym | T^3+^ | (Saha et al. 2016) |
|  | CD4^+^ PD-L1^+^ T cells | PD-L1^+^T^4+^ | | CD3+CD4+CD274+ | Lym | T^4+^ | (Saha et al. 2016) |
|  | CD8^+^ PD-L1^+^ T cells | PD-L1^+^T^4-^ | | CD3+CD4-CD274+ | Lym | T^4-^ | (Ni et al. 2017) |
| LAG3+ T cells | LAG3^+^ T cells | LAG3^+^T | | CD3+LAG3+ | Lym | T^3+^ | (Seifert et al. 2021) |
|  | CD4^+^ LAG3^+^ T cells | LAG3^+^T^4+^ | | CD3+CD4+ LAG3+ | Lym | T^4+^ | (Shapiro et al. 2017) |
|  | CD8^+^ LAG3^+^ T cells | LAG3^+^T^4-^ | | CD3+CD4- LAG3+ | Lym | T^4-^ | (Abdelhakim et al. 2018) |
| 2B4^+^ T cells | 2B4^+^ T cells | 2B4^+^T | | CD3+2B4+ | Lym | T^3+^ | (Chen et al. 2017; Speiser et al. 2001) |
|  | CD4^+^ 2B4^+^ T cells | 2B4^+^T^4+^ | | CD3+CD4+2B4+ | Lym | T^4+^ | (Chen et al. 2017; Speiser et al. 2001) |
|  | CD8^+^ 2B4^+^ T cells | 2B4^+^T^4-^ | | CD3+CD4-2B4+ | Lym | T^4-^ | (Chen et al. 2017; Speiser et al. 2001) |
| Tim3^+^ T cells | Tim3^+^ T cells | Tim3^+^T | | CD3+Tim3+ | Lym | T^3+^ | (Irani et al. 2023; Rezaei et al. 2021) |
|  | CD4^+^ Tim3^+^ T cells | Tim3^+^T^4+^ | | CD3+CD4+Tim3+ | Lym | T^4+^ | (Rezaei et al. 2021; Hong et al. 2022) |
|  | CD8^+^ Tim3^+^ T cells | Tim3^+^T^4-^ | | CD3+CD4-Tim3+ | Lym | T^4-^ | (Rezaei et al. 2021; Hong et al. 2022) |
| KLRG1^+^ T cells | KLRG1^+^ T cells | KLRG1^+^T | | CD3+KLRG1+ | Lym | T^3+^ | (Borys et al. 2022) |
|  | CD4^+^ KLRG1^+^ T cells | KLRG1^+^T^4+^ | | CD3+CD4+ KLRG1+ | Lym | T^4+^ | (Minnie et al. 2022) |
|  | CD8^+^ KLRG1^+^ T cells | KLRG1^+^T^4-^ | | CD3+CD4- KLRG1+ | Lym | T^4-^ | (Cohen et al. 2023) |
| TIGIT^+^ T cells | TIGIT^+^ T cells | TIGIT^+^T | | CD3+TIGIT+ | Lym | T^3+^ | (Zhang et al. 2018) |
|  | CD4^+^TIGIT^+^ T cells | TIGIT^+^T^4+^ | | CD3+CD4+TIGIT+ | Lym | T^4+^ | (Gournay et al. 2022) |
|  | CD8^+^ TIGIT^+^ T cells | TIGIT^+^T^4-^ | | CD3+CD4-TIGIT+ | Lym | T^4-^ | (Minnie et al. 2022) |
| **II. B cells** | | | | | | | |
| Activated and differentiated subsets | | | | | | | |
|  | B cells | B | | CD19+ | Lym | B | (Shimabukuro-Vornhagen et al. 2009) |
|  | Naive B cells | B_nai_ | | CD19+CD27-IgD+ | Lym | B | (Ogonek et al. 2016) |
|  | Class switched Memory B cells | B_mem(class)_ | | CD19+CD27+IgD- | Lym | B | (Sarantopoulos et al. 2009) |
|  | Memory B cells | B_mem_ | | CD19+CD27+IgD+ | Lym | B | (Ogonek et al. 2016) |
|  | Suppressive Regulatory B cells | B_reg_ | | CD19+CD24++CD38++ | Lym | B | (Ramzi et al. 2025) |

Supp. Table 1c: Innate cells

| **I. NK Cells** | | | | | | |
| --- | --- | --- | --- | --- | --- | --- |
|  | Name of Subgroups | Abbreviation of Subgroup | Surface Marker | /Cells | /Subtypes | Reference |
| Activated and differentiated NK subtypes | | | | | | |
|  | CD56^+^ NK cells | NK^56+^ | CD3-CD56+ | Lym | - | (Boeck et al. 2017) |
|  | CD56^bri^ NK cells | NK^56bri^ | CD3-CD56++ | Lym | - | (Lauener et al. 2023) |
|  | CD56^bri^CD16^-^ NK cells | NK^56bri16-^ | CD3-CD56++CD16- | Lym | - | (Ni et al. 2019) |
|  | CD56^bri^CD16^+^ NK cells | NK^56bri16+^ | CD3-CD56++CD16+ | Lym | - | (Huenecke et al. 2017) |
|  | CD56^dim^CD16^+^ NK cells | NK^56dim16+^ | CD3-CD56dimCD16+ | Lym | - | (Pfeiffer et al. 2010) |
|  | CD56^bri^CD57^-^ NK cells | NK^56bri57-^ | CD3-CD56++57- | Lym | - | (Björkström et al. 2010) |
|  | CD56^dim^CD57^-^ NK cells | NK^56dim57-^ | CD3-CD56dim57- | Lym | - | (Björkström et al. 2010) |
|  | CD56^dim^CD57^+^ NK cells | NK^56dim57+^ | CD3-CD56dimCD57+ | Lym | - | (Björkström et al. 2010) |
|  | CD56^dim^CD57^-^CD16^bri^ NK cells | NK^56dim57-16bri^ | CD3- CD56dimCD57-CD16++ | Lym | - | (Lopez-Vergès et al. 2010; Nielsen et al. 2013) |
|  | CD56^dim^CD57^+^CD16^+^NK cells | NK^56dim57+16+^ | CD3-CD56dimCD57+CD16+ | Lym | - | (Lopez-Vergès et al. 2010; Nielsen et al. 2013) |
| Suppressive and checkpoint marker expressing NK subtypes | | | | | | |
|  | CTLA-4^+^ NK cells | CTLA-4^+^NK^56+^ | CD3-CD56+CD152+ | Lym | NK^56+^ | (Parvini et al. 2024) |
|  | PD-1^+^ NK cells | PD-1^+^NK^56+^ | CD3-CD56+CD279+ | Lym | NK^56+^ | (Hsu et al. 2018) |
|  | PD-L1^+^ NK cells | PD-L1^+^NK^56+^ | CD3-CD56+CD274+ | Lym | NK^56+^ | (Sierra et al. 2021) |
|  | PD-L2^+^ NK cells | PD-L2^+^NK^56+^ | CD3-CD56+CD273+ | Lym | NK^56+^ | (Wang et al. 2023) |
|  | CD40L^+^ NK cells | CD40L^+^NK^56+^ | CD3-CD56+CD154+ | Lym | NK^56+^ | (Carbone et al. 1997) |
|  | LAG3^+^ NK cells | LAG3^+^NK^56+^ | CD3-CD56+LAG3+ | Lym | NK^56+^ | (Köhler et al. 2021) |
|  | 2B4^+^ NK cells | 2B4^+^NK^56+^ | CD3-CD56+2B4+ | Lym | NK^56+^ | (Altvater et al. 2009) |
|  | Tim3^+^ NK cells | Tim3^+^NK^56+^ | CD3-CD56+Tim3+ | Lym | NK^56+^ | (Vago and Gojo 2020) |
|  | KLRG1^+^ NK cells | KLRG1^+^NK^56+^ | CD3-CD56+KLRG1+ | Lym | NK^56+^ | (Borys et al. 2022) |
|  | TIGIT^+^ NK cells | TIGIT^+^NK^56+^ | CD3-CD56+TIGIT+ | Lym | NK^56+^ | (Hasan et al. 2023) |
| **II. CIK Cells** | | | | | | |
| Activated and differentiated CIK cells | | | | | | |
|  | CIK cells | CIK^56+^ | CD3+CD56+ | Lym | - | (Boeck et al. 2017) |
|  | CD40L^+^ CIK cells | CD40L^+^CIK^56+^ | CD3+CD56+CD154+ | Lym | CIK^56+^ | (Zhang et al. 2022) |
|  | Mucosal-associated invariant CIK cells | MAIT cells CD^III+^CD^56+^ | CD3+CD161+ | Lym | - | (Talvard-Balland et al. 2024) |
|  | CD40L^+^ CIK cells | CD40L^+^CIK^56+^ | CD3+CD56+CD154+ | Lym | - | - |
| Suppressive and Checkpoint marker expressing CIK cells | | | | | | |
|  | CTLA-4^+^ CIK cells | CTLA-4^+^CIK^56+^ | CD3+CD56+CD152+ | Lym | CIK^56+^ | (Zhang et al. 2022) |
|  | PD-1^+^ CIK cells | PD-1^+^CIK^56+^ | CD3+CD56+CD279+ | Lym | CIK^56+^ | (Li et al. 2022) |
|  | PD-L1^+^ CIK cells | PD-L1^+^CIK^56+^ | CD3+CD56+CD274+ | Lym | CIK^56+^ | (Li et al. 2022) |
|  | PD-L2^+^ CIK cells | PD-L2^+^CIK^56+^ | CD3+CD56+CD273+ | Lym | CIK^56+^ | (Li et al. 2024b) |
|  | LAG3^+^ CIK cells | LAG3^+^CIK^56+^ | CD3+CD56+LAG3+ | Lym | CIK^56+^ | (Li et al. 2024b) |
|  | 2B4^+^ CIK cells | 2B4^+^CIK^56+^ | CD3+CD56+2B4+ | Lym | CIK^56+^ | - |
|  | Tim3^+^ CIK cells | Tim3^+^CIK^56+^ | CD3+CD56+Tim3+ | Lym | CIK^56+^ | - |
|  | KLRG1^+^ CIK cells | KLRG1^+^CIK^56+^ | CD3+CD56+KLRG1+ | Lym | CIK^56+^ | - |
|  | TIGIT^+^ CIK cells | TIGIT^+^CIK^56+^ | CD3+CD56+TIGIT+ | Lym | CIK^56+^ | - |
| **III. iNKT cells** | | | | | | |
| Activated and differentiated iNKT cells | | | | | | |
|  | iNKT cells | iNKT | 6B11+ | Lym | - | (Mavers et al. 2017) |
|  | T cell like iNKT cells | iNKT^3+^ | 6B11+CD3+ | Lym | 6B11 | (Nakamura and Matsuoka 2019) |
|  | NK cell like iNKT cells | iNKT^161+^ | 6B11+CD161+ | Lym | 6B11 | (van der Waart et al. 2012) |

# **Supplementary Table 2 — Grade of chronic GvHD: severe vs. mild-moderate**

| Myeloid cells | | | | | | | | | |
| --- | --- | --- | --- | --- | --- | --- | --- | --- | --- |
| In non Lym-gate | | | | | In subsets | | | | |
| Grade of cGvHD | Mild/moderate vs. severe | N | % Mean  SD | *p* | Grade of cGvHD | Mild/moderate vs. severe | N | % Mean  SD | *p* |
| CD14^+^/non Lym | mild/moderate | 7 | 8.53±6.71 |  |  |  |  |  |  |
|  | severe | 9 | 6.06±5.58 |  |  |  |  |  |  |
| CTLA-4^+^Mo/non Lym | mild/moderate | 7 | 1.68±2.29 |  | CTLA-4^+^Mo/Mo | mild/moderate | 6 | 16.23±6.22 |  |
|  | severe | 10 | 1.67±1.86 |  |  | severe | 9 | 18.20±4.64 |  |
| PD-1^+^Mo/non Lym | mild/moderate | 7 | 1.86±2.03 | significant (**) | PD-1^+^Mo/Mo | mild/moderate | 6 | 15.59±16.11 |  |
|  | severe | 10 | 0.98±0.97 |  |  | severe | 10 | 14.33±7.22 |  |
|  | mild/moderate | 7 | 5.90±2.44 |  | PD-L1^+^Mo/Mo | mild/moderate | 6 | 91.13±9.37 |  |
|  | severe | 9 | 3.51±2.67 |  |  | severe | 8 | 85.95±12.92 |  |
| LAG3^+^Mo/non Lym | mild/moderate | 8 | 0.12±0.03 |  | LAG3^+^Mo/Mo | mild/moderate | 7 | 1.34±0.81 |  |
|  | severe | 10 | 0.09±0.04 |  |  | severe | 10 | 1.84±1.11 |  |
| 2B4^+^Mo/non Lym | mild/moderate | 6 | 6.70±2.68 |  | 2B4^+^Mo/Mo | mild/moderate | 6 | 99.45±0.33 | Highly significant (***) |
|  | severe | 8 | 4.91±2.66 |  |  | severe | 10 | 94.59±9.03 |  |
| Tim3^+^Mo/non Lym | mild/moderate | 6 | 4.73±3.00 |  | Tim3^+^Mo/Mo | mild/moderate | 5 | 92.92±4.21 |  |
|  | severe | 8 | 4.51±2.45 |  |  | severe | 10 | 88.75±7.87 |  |
| DC^80+^/non Lym | mild/moderate | 8 | 1.36±0.89 |  |  |  |  |  |  |
|  | severe | 9 | 1.15±0.57 |  |  |  |  |  |  |
| DC_mat_^80+^/non Lym | mild/moderate | 8 | 0.43±0.24 |  | DC_mat_^80+^/DC^80+^ | mild/moderate | 6 | 30.57±10.24 |  |
|  | severe | 10 | 0.37±0.49 |  |  | severe | 10 | 21.54±11.78 |  |
| DC_tol_^80+^/non Lym | mild/moderate | 8 | 0.21±0.16 |  | DC_tol_^80+^/DC^80+^ | mild/moderate | 6 | 19.64±12.67 |  |
|  | severe | 10 | 0.52±0.28 |  |  | severe | 8 | 35.05±16.00 |  |
| CTLA-4^+^DC^80+^/non Lym | mild/moderate | 8 | 0.51±0.38 | significant (**) | CTLA-4^+^DC^80+^/DC^80+^ | mild/moderate | 6 | 30.15±1.,80 |  |
|  | severe | 10 | 0.24±0.10 |  |  | severe | 8 | 19.47±13.32 |  |
| CTLA-4^+^DC_mat_^80+^/non Lym | mild/moderate | 8 | 0.30±0.21 | borderline significant (*) | CTLA-4^+^DC_mat_^80+^/DC_mat_^80+^ | mild/moderate | 7 | 68.74±18.89 |  |
|  | severe | 10 | 0.15±0.08 |  |  | severe | 9 | 72.50±19.43 |  |
| PMN-MDSC/non Lym | mild/moderate | 7 | 22.19±17.38 |  | PMN-MDSC/CD11b+CD33^+^ | mild/moderate | 7 | 68.70±17.16 |  |
|  | severe | 10 | 16.40±12.09 |  |  | severe | 9 | 71.38±19.90 |  |
| M-MDSC/non Lym | mild/moderate | 7 | 8.57±7.49 |  | M-MDSC/CD11b+CD33^+^ | mild/moderate | 6 | 26.44±12.43 |  |
|  | severe | 9 | 6.37±5.69 |  |  | severe | 8 | 23.04±11.47 |  |
| CTLA-4^+^PMN-MDSC/non Lym | mild/moderate | 7 | 1.86±2.93 | Borderline significant (*) | CTLA-4^+^PMN-MDSC/PMN-MDSC | mild/moderate | 6 | 10.57±14.01 | Significant (**) |
|  | severe | 10 | 1.30±0.69 |  |  | severe | 10 | 10.72±7.07 |  |
| CTLA-4^+^M-MDSC/non Lym | mild/moderate | 8 | 3.83±4.96 | significant (**) | CTLA-4^+^M-MDSC/PMN-MDSC | mild/moderate | 5 | 22.83±11.67 |  |
|  | severe | 10 | 2.03±2.18 |  |  | severe | 9 | 22.69±6.34 |  |
| In non Lym-Gate | | | | | In subsets | | | | |
| Grade of cGvHD | Mild/moderate vs. severe | N | % Mean  SD | *p* | Grade of cGvHD | Mild/moderate vs. severe | N | % Mean  SD | *p* |
| Adaptive cells | | | | | | | | | |
| T/Lym | mild/moderate | 7 | 0.03±0.02 | borderline significant (*) |  |  |  |  |  |
|  | severe | 9 | 0.07±0.09 |  |  |  |  |  |  |
| T^4+^/Lym | mild/moderate | 7 | 1.07±0.82 |  | T^4+^/T^3+^ | mild/moderate | 4 | 35.80±25.08 |  |
|  | severe | 9 | 1.73±1.80 |  |  | severe | 9 | 25.89±10.39 |  |
| T^4-/^Lym | mild/moderate | 7 | 1.82±2.21 |  | T^4-^/T^3+^ | mild/moderate | 5 | 61.00±19.82 | Borderline significant (**) |
|  | severe | 7 | 2,76±2,14 |  |  | severe | 9 | 74.11±10.39 |  |
| T_prol-early_ /Lym | mild/moderate | 7 | 0.25±0.23 |  | T_prol-early_ /T^3+^ | mild/moderate | 6 | 5.37±4.08 |  |
|  | severe | 9 | 0.48±0.32 |  |  | severe | 9 | 7.81±5.71 |  |
| T_prol-late_ /Lym | mild/moderate | 7 | 0.37±0.50 |  | T_prol-late_ /T^3+^ | mild/moderate | 5 | 4.14±3.72 | highly significant (***) |
|  | severe | 9 | 0.70±0.30 |  |  | severe | 9 | 13.00±9.47 |  |
| T_nn_ /Lym | mild/moderate | 7 | 1.58±1.35 |  | T_nn_ /T^3+^ | mild/moderate | 6 | 70.06±15.60 | Borderline significant (**) |
|  | severe | 8 | 2.09±1.58 |  |  | severe | 9 | 46.69±9.94 |  |
| T_em_ /Lym | mild/moderate | 7 | 1.54±1.36 |  | T_em_ /T^3+^ | mild/moderate | 6 | 68.10±15.48 | Highly significant (**) |
|  | severe | 8 | 2.01±1.55 |  |  | severe | 8 | 46.74±7.51 |  |
| T_cm_ /Lym | mild/moderate | 7 | 0.05±0.04 |  | T_cm_ /T^3+^ | mild/moderate | 7 | 1.96±1.62 | Borderline significant (**) |
|  | severe | 9 | 0.07±0.08 |  |  | severe | 8 | 0.99±0.93 |  |
| CD28^+^T/Lym | mild/moderate | 7 | 1.55±1.12 |  | CD28^+^T/T^3+^ | mild/moderate | 6 | 78.90±16.18 |  |
|  | severe | 7 | 2.24±1.66 |  |  | severe | 7 | 46.89±14.81 |  |
| CD28^+^T^4+^/Lym | mild/moderate | 8 | 1.91±2.60 |  | CD28^+^T^4+^/T^4+^ | mild/moderate | 7 | 92.10±10.08 |  |
|  | severe | 7 | 1.06±0.82 |  |  | severe | 8 | 86.59±11.71 |  |
| CD28^+^T^4-^/Lym | mild/moderate | 7 | 0.57±0.48 |  | CD28^+^T^4-^/T^4-^ | mild/moderate | 6 | 62.26±22.71 |  |
|  | severe | 7 | 1.39±1.45 |  |  | severe | 9 | 28.81±14.48 |  |
| CD40L^+^T^4+^/Lym | mild/moderate | 8 | 0.14±0.22 | borderline significant (*) | CD40L^+^T/T^3+^ | mild/moderate | 3 | 11.60±1.70 |  |
|  | severe | 7 | 0.18±0.17 |  |  | severe | 5 | 2.55±2.03 |  |
| CD40L^+^T^4-^/Lym | mild/moderate | 8 | 0.05±0.07 |  | CD40L^+^T^4+^/T^4+^ | mild/moderate | 7 | 4.85±7.62 |  |
|  | severe | 7 | 0.06±0.06 |  |  | severe | 7 | 9.87±7.65 |  |
|  |  |  |  |  | CD40L^+^T^4-^/T^4-^ | mild/moderate | 8 | 1.61±2.05 |  |
|  |  |  |  |  |  | severe | 7 | 1.45±1.21 |  |
| T^H1/Gui^/Lym | mild/moderate | 7 | 1.42±1.69 | highly significant (***) | T^H1/Gui^/T^4+^ | mild/moderate | 8 | 15.38±9.03 |  |
|  | severe | 10 | 0.22±0.16 |  |  | severe | 8 | 11.60±7.99 |  |
| T^H2/Gui^/Lym | mild/moderate | 8 | 0.53±0.77 | borderline significant (*) | T^H2/Gui^/T^4+^ | mild/moderate | 8 | 8.10±3.86 |  |
|  | severe | 10 | 0.22±0.18 |  |  | severe | 9 | 6.65±2.49 |  |
| T^H17/Gui^/Lym | mild/moderate | 8 | 0.70±1.16 |  | T^H17/Gui^/T^4+^ | mild/moderate | 6 | 7.69±5.12 |  |
|  | severe | 10 | 0.38±0.57 |  |  | severe | 9 | 8.84±7.00 |  |
| γδ^+^T/Lym | mild/moderate | 6 | 6.28±5.84 |  | γδ^+^T/T^3+^ | mild/moderate | 6 | 6.28±5.84 |  |
|  | severe | 8 | 4.74±3.38 |  |  | severe | 8 | 4.74±3.38 |  |
| γδ^+^T^4+^/Lym | mild/moderate | 6 | 0.05±0.04 | Highly significant (**) | γδ^+^T^4+^/T^3+^ | mild/moderate | 6 | 1.22±0.99 | significant (**) |
|  | severe | 9 | 0.01±0.01 |  |  | severe | 9 | 0.49±0.54 |  |
| γδ^+^T^4-^/Lym | mild/moderate | 6 | 0.49±0.90 |  | γδ^+^T^4-^/T^3+^ | mild/moderate | 6 | 5.11±5.39 |  |
|  | severe | 9 | 0.31±0.42 |  |  | severe | 8 | 4.30±3.56 |  |
| Intß7^+^T/Lym | mild/moderate | 6 | 1.09±0.45 | significant (**) | Intß7^+^T/T^3+^ | mild/moderate | 6 | 32.74±18.89 |  |
|  | severe | 9 | 1.81±1.52 |  |  | severe | 8 | 26.79±13.50 |  |
| Intß7^+^T^4+^/Lym | mild/moderate | 6 | 0.53±0.92 |  | Intß7^+^T^4+^/T^3+^ | mild/moderate | 6 | 13.67±7.78 |  |
|  | severe | 9 | 0.32±0.42 |  |  | severe | 7 | 9.97±6.54 |  |
| Intß7^+^T^4-^/Lym | mild/moderate | 6 | 0.67±0.35 | borderline significant (*) | Intß7^+^T^4-^/T^3+^ | mild/moderate | 4 | 27.97±8.85 | Borderline significant (**) |
|  | severe | 9 | 1.34±1.20 |  |  | severe | 8 | 20.01±10.92 |  |
| CD127^+^T/Lym | mild/moderate | 7 | 2.25±1.72 |  | CD127^+^T/T^3^ | mild/moderate | 6 | 74.46±7.10 |  |
|  | severe | 9 | 2.66±2.43 |  |  | severe | 7 | 47.57±18.11 |  |
|  |  |  |  |  | T^4+^_reg_ /T^4+^ | mild/moderate | 7 | 8.59±4.29 |  |
|  |  |  |  |  |  | severe | 7 | 7.36±4.53 |  |
| T^4+^_reg_/Lym | mild/moderate | 7 | 0.12±0.08 | borderline significant (*) | T^4+^_reg_ /T^3+^ | mild/moderate | 7 | 4.77±3.14 | highly significant (***) |
|  | severe | 8 | 0.17±0.14 |  |  | severe | 8 | 2.14±1.17 |  |
|  |  |  |  |  | T^4-^_reg_ /T^4-^ | mild/moderate | 7 | 2.02±1.86 | highly significant (***) |
|  |  |  |  |  |  | severe | 8 | 0.27±0.26 |  |
| T^4-^_reg_/Lym | mild/moderate | 7 | 0.08±0.17 | significant (**) | T^4-^_reg_ /T^3+^ | mild/moderate | 7 | 1.00±0,87 | highly significant (***) |
|  | severe | 8 | 0.02±0.02 |  |  | severe | 8 | 0.20±0.20 |  |
| CTLA-4^+^T/Lym | mild/moderate | 2 | 0.20 |  | CTLA-4^+^T/T^3+^ | mild/moderate | 1 | 0.92 |  |
|  | severe | 0^a^ |  |  |  | severe | 0^a^ |  |  |
| CTLA-4^+^T^4+^/Lym | mild/moderate | 8 | 0.01±0.02 |  | CTLA-4^+^T^4+^/T^4+^ | mild/moderate | 8 | 0.38±0.36 |  |
|  | severe | 9 | 0.02±0.01 |  |  | severe | 8 | 0.72±0.42 |  |
| CTLA-4^+^T^4-^/Lym | mild/moderate | 8 | 0.04±0.06 |  | CTLA-4^+^T^4-^/T^4-^ | mild/moderate | 7 | 1.32±1.24 |  |
|  | severe | 9 | 0.05±0.04 |  |  | severe | 9 | 0.95±0.85 |  |
| CD28^+^CTLA-4^+^T/Lym | mild/moderate | 2 | 0.11 |  | CD28^+^CTLA-4^+^T^4-^/T^4-^ | mild/moderate | 8 | 0.34±0.33 |  |
|  | severe | 0^a^ |  |  |  | severe | 9 | 0.69±0.44 |  |
| CD28^+^CTLA-4^+^T^4+^/Lym | mild/moderate | 8 | 0.06±0.13 | significant (**) | CD28^+^CTLA-4^+^T^4+^/T^4+^ | mild/moderate | 6 | 0.37±0.34 |  |
|  | severe | 9 | 0.01±0.01 |  |  | severe | 9 | 0.36±0.27 |  |
| CD28^+^CTLA-4^+^T^4-^/Lym | mild/moderate | 8 | 0.03±0.04 | significant (**) |  |  |  |  |  |
|  | severe | 9 | 0.02±0.01 |  |  |  |  |  |  |
| PD-1^+^T/Lym | mild/moderate | 8 | 1.77±2.74 |  | PD-1^+^T/T^3+^ | mild/moderate | 8 | 19.37±11.63 |  |
|  | severe | 9 | 0.92±1.08 |  |  | severe | 7 | 20.85±9.96 |  |
| PD-1^+^T^4+^/Lym | mild/moderate | 8 | 0.19±0.23 |  | PD-1^+^T^4+^/T^4+^ | mild/moderate | 7 | 11.81±7.90 |  |
|  | severe | 9 | 0.11±0.12 |  |  | severe | 7 | 12.97±7.14 |  |
| PD-1^+^T^4-^/Lym | mild/moderate | 8 | 1.57±2.52 |  | PD-1^+^T^4-^/T^4-^ | mild/moderate | 8 | 84.61±11.88 |  |
|  | severe | 9 | 0.81±0.99 |  |  | severe | 7 | 87.03±7.14 |  |
| PD-L1^+^T/Lym | mild/moderate | 8 | 1.02±1.22 |  | PD-L1^+^T/T^3+^ | mild/moderate | 8 | 9.07±3.79 |  |
|  | severe | 9 | 0.49±0.60 |  |  | severe | 7 | 14.00±6.12 |  |
| PD-L1^+^T^4+^/Lym | mild/moderate | 8 | 0.63±0.68 |  | PD-L1^+^T^4+^/T^4+^ | mild/moderate | 8 | 64.00±21.20 |  |
|  | severe | 9 | 0.29±0.39 |  |  | severe | 6 | 61.98±19.83 |  |
| PD-L1^+^T^4-^/Lym | mild/moderate | 8 | 0.39±0.64 |  | PD-L1^+^T^4-^/T^4-^ | mild/moderate | 7 | 35.99±21.21 |  |
|  | severe | 9 | 0.19±0.22 |  |  | severe | 6 | 38.02±19.83 |  |
| LAG3^+^T/Lym | mild/moderate | 2 | 0.14 |  | LAG3^+^T/T^3+^ | mild/moderate | 0^a^ | |  |
|  | severe | 1 | 0.08 |  |  | severe | 0^a^ |  |  |
| LAG3^+^T^4+^/Lym | mild/moderate | 8 | 0.02±0.03 |  | LAG3^+^T^4+^/T^4+^ | mild/moderate | 7 | 0.73±0.58 |  |
|  | severe | 10 | 0.02±0.01 |  |  | severe | 8 | 0.92±0.40 |  |
| LAG3^+^T^4-^/Lym | mild/moderate | 8 | 0.08±0.12 | highly significant (***) | LAG3^+^T^4-^/T^4-^ | mild/moderate | 6 | 0.99±0.50 |  |
|  | severe | 10 | 0.04±0.02 |  |  | severe | 10 | 0.99±0.87 |  |
| 2B4^+^T/Lym | mild/moderate | 3 | 4.97±6.68 | highly significant (***) | 2B4^+^T/T^3+^ | mild/moderate | 1 | 43.40 |  |
|  | severe | 8 | 1.91±1.00 |  |  | severe | 9 | 35.84±14.69 |  |
| 2B4^+^T^4+^/Lym | mild/moderate | 8 | 0.03±0.03 | significant (**) | 2B4^+^T^4+^/T^4+^ | mild/moderate | 8 | 1.02±0.83 | significant (**) |
|  | severe | 9 | 0.22±0.38 |  |  | severe | 7 | 5.50±8.23 |  |
| 2B4^+^T^4-^/Lym | mild/moderate | 7 | 0.08±0.06 | highly significant (***) | 2B4^+^T^4-^/T^4-^ | mild/moderate | 3 | 32.87±21.58 |  |
|  | severe | 8 | 1.70±1.17 |  |  | severe | 9 | 46.66±17.02 |  |
| TIM3^+^T/Lym | mild/moderate | 5 | 0.35±0.26 |  | TIM3^+^T/T^3+^ | mild/moderate | 2 | 2.41±0.76 | significant (**) |
|  | severe | 7 | 0.34±0.17 |  |  | severe | 8 | 6.96±5.62 |  |
| TIM3^+^T^4+^/Lym | mild/moderate | 8 | 0.04±0.04 |  | TIM3^+^T^4+^/T^4+^ | mild/moderate | 7 | 1.37±0.84 |  |
|  | severe | 9 | 0.04±0.04 |  |  | severe | 7 | 1.69±1.03 |  |
| TIM3^+^T^4-^/Lym | mild/moderate | 8 | 0.19±0.20 |  | TIM3^+^T^4-^/T^4-^ | mild/moderate | 6 | 3.39±1.74 | highly significant (***) |
|  | severe | 9 | 0.24±0.16 |  |  | severe | 9 | 7.81±6.63 |  |
| KLRG1^+^T/Lym | mild/moderate | 7 | 2.61±2.61 |  | KLRG1^+^T/T^3+^ | mild/moderate | 8 | 35.11±20.72 |  |
|  | severe | 9 | 1.18±2.27 |  |  | severe | 5 | 39.46±15.12 |  |
| KLRG1^+^T^4+^/Lym | mild/moderate | 8 | 0.50±0.62 | highly significant (***) | KLRG1^+^T^4+^/T^4+^ | mild/moderate | 7 | 12.98±7.12 | significant (**) |
|  | severe | 9 | 0.09±0.11 |  |  | severe | 8 | 13.06±13.68 |  |
| KLRG1^+^T^4+^/Lym | mild/moderate | 8 | 2.88±2.61 |  | KLRG1^+^T4-/T4- | mild/moderate | 8 | 82.24±15.54 |  |
|  | severe | 9 | 1.10±2.28 |  |  | severe | 8 | 86.95±13.69 |  |
| TIGIT^+^T/Lym | mild/moderate | 6 | 2.07±1.69 |  | TIGIT^+^T/T^3+^ | mild/moderate | 7 | 35.52±11.30 |  |
|  | severe | 9 | 1.52±2.08 |  |  | severe | 8 | 35.33±14.84 |  |
| TIGIT^+^T^4+^/Lym | mild/moderate | 8 | 1.18±0.94 | highly significant (***) | TIGIT^+^T^4+^/T^4+^ | mild/moderate | 7 | 62.69±29.34 |  |
|  | severe | 9 | 0.26±0.30 |  |  | severe | 8 | 70.75±25.41 |  |
| TIGIT^+^T^4+^/Lym | mild/moderate | 8 | 2.77±3.02 | significant (**) | TIGIT^+^T^4-^/T^4-^ | mild/moderate | 7 | 37.75±30.29 |  |
|  | severe | 9 | 1.26±1.84 |  |  | severe | 8 | 29.25±25.41 |  |
| B/Lym | mild/moderate | 6 | 0.06±0.10 |  |  |  |  |  |  |
|  | severe | 9 | 0.04±0.04 |  |  |  |  |  |  |
| B_nai_/Lym | mild/moderate | 6 | 5.52±8.81 | borderline significant (*) | B_nai_/B | mild/moderate | 4 | 59.08±36.90 | highly significant (***) |
|  | severe | 9 | 2.98±4.01 |  |  | severe | 8 | 62.01±19.46 |  |
| B_mem(class)_/Lym | mild/moderate | 6 | 0.21±0.32 |  | B_mem(class)/_B | mild/moderate | 6 | 9.05±7.96 |  |
|  | severe | 9 | 0.55±0.57 |  |  | severe | 8 | 18.80±12.89 |  |
| B_mem_/Lym | mild/moderate | 6 | 0.10±0.14 |  | B_mem_/B | mild/moderate | 6 | 2.52±3.40 |  |
|  | severe | 9 | 0.24±0.34 |  |  | severe | 9 | 5.59±5.10 |  |
| B_reg_/Lym | mild/moderate | 0^a^ |  |  | B_reg_/B | mild/moderate | 6 | 3.63±2.71 | highly significant (***) |
|  | severe | 0^a^ |  |  |  | severe | 9 | 0.98±0.96 |  |
| Innate cells | | | | | | | | | |
| In Lym-Gate | | | | | In subsets | | | | |
| Grade of cGvHD | Mild/moderate vs. severe | N | % Mean  SD | *p* | Grade of cGvHD | Mild/moderate vs. severe | N | % Mean  SD | *p* |
| NK^56+^/Lym | mild/moderate | 7 | 0.06±.,05 |  |  |  |  |  |  |
|  | severe | 9 | 0.06±0.07 |  |  |  |  |  |  |
| NK^56bri^/Lym | mild/moderate | 5 | 3.40±4.14 | highly significant (***) | NK^56bri^/NK^56+^ | mild/moderate | 5 | 20.87±15.63 |  |
|  | severe | 9 | 0.79±1.27 |  |  | severe | 9 | 11.53±11.16 |  |
| NK^56bri16-^/Lym | mild/moderate | 6 | 3.44±4.02 | highly significant (***) | NK^56bri16-^/NK^56+^ | mild/moderate | 6 | 12.00±13.99 | significant (**) |
|  | severe | 9 | 0.37±0.63 |  |  | severe | 9 | 4.53±4.25 |  |
| NK^56bri16+^/Lym | mild/moderate | 6 | 3.98±6.94 | significant (**) | NK^56bri16+^/NK^56+^ | mild/moderate | 6 | 10.53±5.81 |  |
|  | severe | 9 | 0.42±0.65 |  |  | severe | 8 | 4.70±3.68 |  |
| NK^56dim16+^/Lym | mild/moderate | 6 | 22.44±34.43 | highly significant (***) | NK^56dim16+^/NK^56+^ | mild/moderate | 7 | 57.49±20.12 |  |
|  | severe | 9 | 4.23±5.59 |  |  | severe | 7 | 79.94±14.49 |  |
| NK^56bri57-^/Lym | mild/moderate | 5 | 3.46±4.07 | highly significant (***) | NK^56bri57-^/NK^56+^ | mild/moderate | 5 | 22.63±15.68 |  |
|  | severe | 9 | 0.79±1.30 |  |  | severe | 9 | 10.94±10.76 |  |
| NK^56dim57-^/Lym | mild/moderate | 4 | 2.36±2.00 |  | NK^56dim57-^/NK^56+^ | mild/moderate | 6 | 45.02±8.77 |  |
|  | severe | 9 | 3.42±4.78 |  |  | severe | 8 | 51.56±20.03 |  |
| NK^56dim57+^/Lym | mild/moderate | 6 | 4.91±6.47 | borderline significant (*) | NK^56dim57+^/NK^56+^ | mild/moderate | 7 | 26.09±16.25 |  |
|  | severe | 9 | 1.93±1.86 |  |  | severe | 6 | 25.19±19.63 |  |
| NK^56dim57-16bri^/Lym | mild/moderate | 6 | 8.70±12.44 | significant (**) | NK^56dim57-16bri^/NK^56+^ | mild/moderate | 7 | 28.89±14.15 |  |
|  | severe | 9 | 2.18±4.17 |  |  | severe | 8 | 20.01±10.71 |  |
| NK^56dim57+16+^/Lym | mild/moderate | 6 | 4.89±6.47 | borderline significant (*) | NK^56dim57+16+^/NK^56+^ | mild/moderate | 7 | 26.03±16.26 |  |
|  | severe | 9 | 1.92±1.85 |  |  | severe | 7 | 31.62±24.95 |  |
| CTLA-4^+^NK^56+^/Lym | mild/moderate | 7 | 0.76±0.73 | significant (**) | CTLA-4^+^NK^56+^/NK^56+^ | mild/moderate | 7 | 40.37±17.03 |  |
|  | severe | 10 | 1.34±1.41 |  |  | severe | 8 | 27.20±10.39 |  |
| PD-1^+^NK^56+^/Lym | mild/moderate | 8 | 0.03±0.04 | borderline significant (*) | PD-1^+^NK^56+^/NK^56+^ | mild/moderate | 7 | 16.04±14.31 |  |
|  | severe | 10 | 0.08±0.08 |  |  | severe | 10 | 11.56±9.82 |  |
| PD-L1^+^NK^56+^/Lym | mild/moderate | 8 | 0.07±0.12 | borderline significant (*) | PD-L1^+^NK^56+^/NK^56+^ | mild/moderate | 8 | 21.85±16.34 | borderline significant (*) |
|  | severe | 9 | 0.06±0.04 |  |  | severe | 8 | 11.88±9.27 |  |
| PD-L2^+^NK^56+^/Lym | mild/moderate | 7 | 0.06±0.07 |  | PD-L2^+^NK^56+^/NK^56+^ | mild/moderate | 8 | 1.29±0.92 |  |
|  | severe | 10 | 0.05±0.06 |  |  | severe | 9 | 1.13±1.14 |  |
| CD40L^+^NK^56+^/Lym | mild/moderate | 8 | 0.06±0.05 | significant (**) | CD40L^+^NK^56+^/NK^56+^ | mild/moderate | 7 | 12.78±12.54 |  |
|  | severe | 10 | 0.12±0.16 |  |  | severe | 10 | 18.74±15.79 |  |
| LAG3^+^NK^56+^/Lym | mild/moderate | 7 | 0.03±0.02 |  | LAG3^+^NK^56+^/NK^56+^ | mild/moderate | 6 | 84.53±12.90 | significant (**) |
|  | severe | 10 | 0.04±0.04 |  |  | severe | 9 | 92.01±6.66 |  |
| 2B4^+^NK^56+^/Lym | mild/moderate | 6 | 2.47±2.82 |  | 2B4^+^NK^56+^/NK^56+^ | mild/moderate | 7 | 62.30±23.22 |  |
|  | severe | 9 | 3.45±2.87 |  |  | severe | 7 | 68.77±14.13 |  |
| TIM3^+^NK^56+^/Lym | mild/moderate | 6 | 1.20±0.77 |  | TIM3^+^NK^56+^/NK^56+^ | mild/moderate | 7 | 55.00±8.40 |  |
|  | severe | 9 | 1.85±1.47 |  |  | severe | 9 | 57.77±19.18 |  |
| KLRG1^+^NK^56+^/Lym | mild/moderate | 8 | 1.23±1.02 |  | KLRG1^+^NK^56+^/NK^56+^ | mild/moderate | 8 | 22.87±16.52 |  |
|  | severe | 10 | 1.08±1.22 |  |  | severe | 9 | 23.68±13.57 |  |
| TIGIT^+^NK^56+^/Lym | mild/moderate | 7 | 1.58±1.94 |  | TIGIT^+^NK^56+^/NK^56+^ | mild/moderate | 6 | 27.93±11.75 |  |
|  | severe | 10 | 1.87±1.51 |  |  | severe | 9 | 32.70±15.44 |  |
| CIK^56+^/Lym | mild/moderate | 8 | 0.41±0.44 |  | CD40L^+^CIK^56+^/CIK^56^ | mild/moderate | 8 | 13.66±14.90 |  |
|  | severe | 10 | 0.31±0.32 |  |  | severe | 9 | 6.62±6.58 |  |
| CD40L^+^CIK^56+^/Lym | mild/moderate | 8 | 0.26±0.29 |  | CTLA-4^+^CIK^56+^/CIK^56+^ | mild/moderate | 7 | 14.00±7.81 |  |
|  | severe | 10 | 0.21±0.27 |  |  | severe | 9 | 17.66±12.39 |  |
| MAIT cells /Lym | mild/moderate | 8 | 0.00±0.00 |  | PD-1^+^CIK^56+^/CIK^56+^ | mild/moderate | 8 | 3.75±2.65 | borderline significant (*) |
|  | severe | 9 | 0.00±0.01 |  |  | severe | 9 | 6.48±5.12 |  |
| CTLA-4^+^CIK56+/Lym | mild/moderate | 8 | 0.04±0.05 |  | PD-L1^+^CIK^56+^/CIK^56+^ | mild/moderate | 7 | 14.04±12.00 | highly significant (***) |
|  | severe | 10 | 0.04±0.03 |  |  | severe | 8 | 6.53±3.44 |  |
| PD-1^+^CIK^56+^/Lym | mild/moderate | 8 | 0.21±0.24 |  | PD-L2^+^CIK^56+^/CIK^56+^ | mild/moderate | 8 | 8.79±9.31 |  |
|  | severe | 10 | 0.12±0.27 |  |  | severe | 9 | 10.34±11.15 |  |
| PD-L1^+^CIK^56+^/Lym | mild/moderate | 8 | 0.47±0.37 | highly significant (***) | LAG3^+^CIK^56+^/CIK^56+^ | mild/moderate | 1 | 2.13 |  |
|  | severe | 10 | 0.10±0.08 |  |  | severe | 6 | 2.00±1.63 |  |
| PD-L2^+^CIK^56+^/Lym | mild/moderate | 8 | 0.09±0.22 | significant (**) | 2B4^+^CIK^56+^/CIK^56+^ | mild/moderate | 2 | 55.65±3.46 | borderline significant (*) |
|  | severe | 10 | 0.02±0.02 |  |  | severe | 9 | 45.51±25.36 |  |
| LAG3^+^CIK^56+/^Lym | mild/moderate | 2 | 0.02±0.01 |  | Tim3^+^CIK^56+^/CIK^56+^ | mild/moderate | 2 | 7.57±4.15 |  |
|  | severe | 6 | 0.01±0.01 |  |  | severe | 6 | 7.43±4.65 |  |
| 2B4^+^CIK^56+^/Lym | mild/moderate | 2 | 0.21±0.02 | borderline significant (*) | KLRG1^+^CIK^56+^/CIK^56+^ | mild/moderate | 8 | 50.13±30.57 | significant (**) |
|  | severe | 9 | 0.33±0.31 |  |  | severe | 7 | 70.69±12.97 |  |
| Tim3^+^CIK^56+^/Lym | mild/moderate | 2 | 0.03±0.03 | borderline significant (*) | TIGIT^+^CIK^56+^/CIK^56+^ | mild/moderate | 6 | 37.27±9.88 | borderline significant (*) |
|  | severe | 6 | 0.06±0.04 |  |  | severe | 8 | 31.08±17.35 |  |
| KLRG1^+^CIK^56+^/Lym | mild/moderate | 8 | 0.30±0.54 | significant (**) | iNKT^3+^/6B11 | mild/moderate | 0^a^ |  |  |
|  | severe | 10 | 0.07±0.06 |  |  | severe | 0^a^ |  |  |
| TIGIT^+^CIK^56+^/Lym | mild/moderate | 8 | 0.26±0.41 | highly significant (***) | iNKT^161+^/6B11 | mild/moderate | 0^a^ |  |  |
|  | severe | 10 | 0.06±0.06 |  |  | severe | 0^a^ |  |  |
| iNKT/Lym | mild/moderate | 7 | 0.34±0.52 | borderline significant (*) |  |  |  |  |  |
|  | severe | 9 | 0.12±0.09 |  |  |  |  |  |  |
| iNKT^3+^/Lym | mild/moderate | 7 | 0.17±0.41 | significant (**) |  |  |  |  |  |
|  | severe | 9 | 0.01±0.01 |  |  |  |  |  |  |
|  |  |  |  |  |  |  |  |  |  |

# **Supplementary Table 3— Chronic GvHD: de novo / quiescent vs progressive**

| Myeloid cells | | | | | | | | | |
| --- | --- | --- | --- | --- | --- | --- | --- | --- | --- |
| In non Lym-gate | | | | | In subsets | | | | |
| De novo/quiescent vs progressive |  | N | % Mean  SD | *p* | De novo/quiescent vs progressive |  | N | % Mean  SD | *p* |
| CD14^+^/non Lym | de novo / quiescent | 11 | 8.44±6.31 |  |  |  |  |  |  |
|  | progressive | 5 | 2.92±2.25 |  |  |  |  |  |  |
| CTLA-4^+^Mo/non Lym | de novo / quiescent | 12 | 2.05±2.13 | borderline significant (*) | CTLA-4^+^Mo/Mo | de novo / quiescent | 11 | 17.30±5.92 |  |
|  | progressive | 5 | 0.53±0.32 |  |  | progressive | 5 | 20.06±5.87 |  |
| PD-1^+^Mo/non Lym | de novo / quiescent | 13 | 1.44±1.59 | significant (**) | PD-1^+^Mo/Mo | de novo / quiescent | 13 | 14.76±11.08 |  |
|  | progressive | 4 | 0.67±0.39 |  |  | progressive | 4 | 13.46±10.46 |  |
| PD-L1^+^Mo/non Lym | de novo / quiescent | 11 | 5.29±2.73 |  | PD-L1^+^Mo/Mo | de novo / quiescent | 12 | 87.44±12.06 |  |
|  | progressive | 5 | 2.89±1.89 |  |  | progressive | 3 | 91.90±5.37 |  |
| LAG3^+^Mo/non Lym | de novo / quiescent | 13 | 0.10±0.04 |  | LAG3^+^Mo/Mo | de novo / quiescent | 13 | 1.56±0.89 |  |
|  | progressive | 5 | 0.09±0.04 |  |  | progressive | 5 | 1.60±1.42 |  |
| 2B4^+^Mo/non Lym | de novo / quiescent | 11 | 6.01±2.65 |  | 2B4^+^Mo/Mo | de novo / quiescent | 13 | 97.72±5.30 |  |
|  | progressive | 3 | 3.80±1.90 |  |  | progressive | 4 | 93.05±12.00 |  |
| Tim3^+^Mo/non Lym | de novo / quiescent | 11 | 4.86±2.57 |  | Tim3^+^Mo/Mo | de novo / quiescent | 11 | 90.48±6.90 |  |
|  | progressive | 3 | 3.49±1.93 |  |  | progressive | 5 | 89.40±7.18 |  |
| DC^80+^/non Lym | de novo / quiescent | 12 | 1.35±0.77 |  |  |  |  |  |  |
|  | progressive | 5 | 1.05±0.46 |  |  |  |  |  |  |
| DC_mat_^80+^/non Lym | de novo / quiescent | 13 | 0.44±0.43 |  | DC_mat_^80+^/DC^80+^ | de novo / quiescent | 13 | 27.55±10.90 |  |
|  | progressive | 5 | 0.23±0.22 |  |  | progressive | 4 | 14.19±7.71 |  |
| DC_tol_^80+^/non Lym | de novo / quiescent | 13 | 0.39±0.26 | significant (**) | DC_tol_^80+^/DC^80+^ | de novo / quiescent | 11 | 28.37±14.69 |  |
|  | progressive | 5 | 0.60±0.53 |  |  | progressive | 3 | 28.73±25.00 |  |
| CTLA-4^+^DC^80+^/non Lym | de novo / quiescent | 13 | 0.38±0.31 |  | CTLA-4^+^DC^80+^/DC^80+^ | de novo / quiescent | 11 | 23.14±13.64 |  |
|  | progressive | 5 | 0.26±0.16 |  |  | progressive | 4 | 25.16±18.62 |  |
| CTLA-4^+^DC_mat_^80+^/non Lym | de novo / quiescent | 13 | 0.23±0.16 |  | CTLA-4^+^DC_mat_^80+^/DC_mat_^80+^ | de novo / quiescent | 12 | 69.79±19.29 |  |
|  | progressive | 5 | 0.16±0.14 |  |  | progressive | 5 | 75.24±16.58 |  |
| PMN-MDSC/non Lym | de novo / quiescent | 12 | 19.79±13.93 |  | PMN-MDSC/CD11b+CD33^+^ | de novo / quiescent | 12 | 69.17±19.00 |  |
|  | progressive | 5 | 12.38±14.79 |  |  | progressive | 5 | 70.18±16.89 |  |
| M-MDSC/non Lym | de novo / quiescent | 11 | 8.72±6.65 |  | M-MDSC/CD11b+CD33^+^ | de novo / quiescent | 10 | 23.62±9.22 | significant (**) |
|  | progressive | 5 | 2.89±2.63 |  |  | progressive | 5 | 29.83±16.88 |  |
| CTLA-4^+^PMN-MDSC/non Lym | de novo / quiescent | 12 | 1.70±2.02 |  | CTLA-4^+^PMN-MDSC/PMN-MDSC | de novo / quiescent | 13 | 11.55±10.64 |  |
|  | progressive | 5 | 0.90±0.65 |  |  | progressive | 4 | 13.66±13.86 |  |
| CTLA-4^+^M-MDSC/non Lym | de novo / quiescent | 13 | 3.40±3.88 | significant (**) | CTLA-4^+^M-MDSC/PMN-MDSC | de novo / quiescent | 11 | 22.50±9.06 |  |
|  | progressive | 5 | 0.69±0.46 |  |  | progressive | 4 | 25.98±6.09 |  |
| In non Lym-Gate | | | | | In subsets | | | | |
| De novo/quiescent vs progressive |  | N | % Mean  SD | *p* | De novo/quiescent vs progressive |  | N | % Mean  SD | *p* |
| Adaptive cells | | | | | | | | | |
| T/Lym | de novo / quiescent | 12 | 0.06±0.08 |  |  |  |  |  |  |
|  | progressive | 4 | 0.02±0.02 |  |  |  |  |  |  |
| T^4+^/Lym | de novo / quiescent | 12 | 1.48±1.61 |  | T^4+^/T^3+^ | de novo / quiescent | 9 | 22.88±9.32 |  |
|  | progressive | 4 | 1.08±1.10 |  |  | progressive | 4 | 42.03±14.81 |  |
| T^4-^/Lym | de novo / quiescent | 10 | 2.61±2.38 | significant (**) | T^4-^/T^3+^ | de novo / quiescent | 11 | 70.65±16.86 |  |
|  | progressive | 4 | 1.10±0.71 |  |  | progressive | 3 | 64.97±5.98 |  |
| T_prol-early_ /Lym | de novo / quiescent | 12 | 0.44±0.32 | significant (**) | T_prol-early_ /T^3+^ | de novo / quiescent | 11 | 7.24±5.48 |  |
|  | progressive | 4 | 0.17±0.07 |  |  | progressive | 4 | 8.31±6.16 |  |
| T_prol-late_ /Lym | de novo / quiescent | 12 | 0.58±0.43 |  | T_prol-late_ /T^3+^ | de novo / quiescent | 10 | 9.24±9.13 |  |
|  | progressive | 4 | 0.40±0.39 |  |  | progressive | 4 | 13.15±7.86 |  |
| T_nn_ /Lym | de novo / quiescent | 11 | 1.93±1.58 |  | T_nn_ /T^3+^ | de novo / quiescent | 11 | 53.97±17.40 |  |
|  | progressive | 4 | 1.30±1.18 |  |  | progressive | 4 | 56.70±12.85 |  |
| T_em_ /Lym | de novo / quiescent | 11 | 1.86±1.56 |  | T_em_ /T^3+^ | de novo / quiescent | 10 | 54.29±15.99 |  |
|  | progressive | 4 | 1.26±1.15 |  |  | progressive | 4 | 54.95±12.85 |  |
| T_cm_ /Lym | de novo / quiescent | 12 | 0.07±0.07 |  | T_cm_ /T^3+^ | de novo / quiescent | 11 | 1.46±1.56 | Significant (**) |
|  | progressive | 4 | 0.03±0.03 |  |  | progressive | 4 | 1.40±0.51 |  |
| CD28^+^T/Lym | de novo / quiescent | 10 | 2.27±1.38 |  | CD28^+^T/T^3+^ | de novo / quiescent | 11 | 61.56±22.62 |  |
|  | progressive | 4 | 0.73±0.84 |  |  | progressive | 2 | 61.55±22.70 |  |
| CD28^+^T^4+^/Lym | de novo / quiescent | 11 | 1.75±2.06 |  | CD28^+^T^4+^/T^4+^ | de novo / quiescent | 13 | 90.36±10.04 |  |
|  | progressive | 4 | 0.44±0.59 |  |  | progressive | 2 | 85.30±20.79 |  |
| CD28^+^T^4-^/Lym | de novo / quiescent | 10 | 1.24±1.22 |  | CD28^+^T^4-^/T^4-^ | de novo / quiescent | 12 | 44.43±23.48 |  |
|  | progressive | 4 | 0.29±0.29 |  |  | progressive | 3 | 35.67±30.54 |  |
| CD40L^+^T^4+^/Lym | de novo / quiescent | 12 | 0.14±0.18 |  | CD40L^+^T/T^3+^ | de novo / quiescent | 5 | 4.11±5.08 |  |
|  | progressive | 3 | 0.33±0.17 |  |  | progressive | 2 | 7.72±3.79 |  |
| CD40L^+^T^4-^/Lym | de novo / quiescent | 12 | 0.05±0.07 | significant (**) | CD40L^+^T^4+^/T^4+^ | de novo / quiescent | 11 | 6.45±7.58 |  |
|  | progressive | 3 | 0.16±0.19 |  |  | progressive | 2 | 13.65±7.86 |  |
|  |  |  |  |  |  | de novo / quiescent | 12 | 1.42±1.71 |  |
|  |  |  |  |  |  | progressive | 2 | 2.23±1.03 |  |
| T^H1/Gui^/Lym | de novo / quiescent | 12 | 0.83±1.30 | significant (**) | T^H1/Gui^/T^4+^ | de novo / quiescent | 13 | 13.99±8.55 |  |
|  | progressive | 5 | 0.22±0.10 |  |  | progressive | 3 | 9.41±6.15 |  |
| T^H2/Gui^/Lym | de novo / quiescent | 13 | 0.39±0.58 |  | T^H2/Gui^/T^4+^ | de novo / quiescent | 12 | 7.98±3.23 |  |
|  | progressive | 5 | 0.28±0.16 |  |  | progressive | 5 | 6.79±3.87 |  |
| T^H17/Gui^/Lym | de novo / quiescent | 13 | 0.53±0.93 |  | T^H17/Gui^/T^4+^ | de novo / quiescent | 11 | 8.68±6.38 |  |
|  | progressive | 5 | 0.39±0.52 |  |  | progressive | 4 | 6.36±5.96 |  |
| γδ^+^T/Lym | de novo / quiescent | 11 | 5.62±4.62 |  | γδ^+^T/T^3+^ | de novo / quiescent | 11 | 5.62±4.62 |  |
|  | progressive | 3 | 5.10±2.86 |  |  | progressive | 3 | 5.10±2.86 |  |
| γδ^+^T^4+^/Lym | de novo / quiescent | 11 | 0.03±0.03 |  | γδ^+^T^4+^/T^3+^ | de novo / quiescent | 11 | 0.68±0.90 |  |
|  | progressive | 4 | 0.02±0.02 |  |  | progressive | 4 | 1.04±0.47 |  |
| γδ^+^T^4-^/Lym | de novo / quiescent | 11 | 0.43±0.67 | borderline significant (**) | γδ^+^T^4-^/T^3+^ | de novo / quiescent | 11 | 4.94±4.43 | borderline significant (*) |
|  | progressive | 4 | 0.14±0.14 |  |  | progressive | 3 | 4.00±2.38 |  |
| Intß7^+^T/Lym | de novo / quiescent | 11 | 1.74±1.36 |  | Intß7^+^T/T^3+^ | de novo / quiescent | 10 | 29.28±16.25 |  |
|  | progressive | 4 | 0.70±0.62 |  |  | progressive | 4 | 32.74±14.81 |  |
| Intß7^+^T^4+^/Lym | de novo / quiescent | 11 | 0.45±0.69 |  | Intß7^+^T^4+^/T^3+^ | de novo / quiescent | 10 | 9.74±6.07 | borderline significant (*) |
|  | progressive | 4 | 0.16±0.14 |  |  | progressive | 3 | 19.80±3.08 |  |
| Intß7^+^T^4-^/Lym | de novo / quiescent | 11 | 1.29±1.07 |  | Intß7^+^T^4-^/T^3+^ | de novo / quiescent | 8 | 25.22±10.87 |  |
|  | progressive | 4 | 0.33±0.25 |  |  | progressive | 4 | 17.32±7.27 |  |
| CD127^+^T/Lym | de novo / quiescent | 11 | 2.62±2.36 |  | CD127^+^T/T^3+^ | de novo / quiescent | 10 | 57.13±20.55 |  |
|  | progressive | 5 | 1.77±1.50 |  |  | progressive | 3 | 57.13±21.17 |  |
|  |  |  |  |  | T^4+^_reg_ /T^4+^ | de novo / quiescent | 11 | 7.98±4.15 |  |
|  |  |  |  |  |  | progressive | 2 | 7.64±6.88 |  |
| T^4+^_reg_/Lym | de novo / quiescent | 12 | 0.15±0.12 |  | T^4+^_reg_ /T^3+^ | de novo / quiescent | 12 | 2.82±2.05 |  |
|  | progressive | 3 | 0.11±0.09 |  |  | progressive | 3 | 5.54±3.89 |  |
|  |  |  |  |  | T4^-^_reg_ /T^4-^ | de novo / quiescent | 12 | 1.12±1.58 |  |
|  |  |  |  |  |  | progressive | 3 | 0.36±0.23 |  |
| T^4-^_reg_/Lym | de novo / quiescent | 12 | 0.05±0.12 |  | T4^-^_reg_ /T^3+^ | de novo / quiescent | 12 | 0.60±0.74 |  |
|  | progressive | 3 | 0.01±0.01 |  |  | progressive | 3 | 0.20±0.13 |  |
| CTLA-4^+^T/Lym | de novo / quiescent | 1 | 0.20 |  | CTLA-4^+^T/T^3+^ | de novo / quiescent | 1 | 0.92 |  |
|  | progressive | 0^a^ |  |  |  | progressive | 0^a^ |  |  |
| CTLA-4^+^T^4+^/Lym | de novo / quiescent | 13 | 0.02±0.02 |  | CTLA-4^+^T^4+^/T^4+^ | de novo / quiescent | 12 | 0.48±0.31 |  |
|  | progressive | 4 | 0.01±0.01 |  |  | progressive | 4 | 1.28±0.95 |  |
| CTLA-4^+^T^4-^/Lym | de novo / quiescent | 13 | 0.05±0.05 |  | CTLA-4^+^T^4-^/T^4-^ | de novo / quiescent | 13 | 1.08±1.09 |  |
|  | progressive | 4 | 0.02±0.02 |  |  | progressive | 4 | 1.20±0.61 |  |
| CD28^+^CTLA-4^+^T/Lym | de novo / quiescent | 1 | 0.11 |  | CD28^+^CTLA-4^+^T^4-^/T^4-^ | de novo / quiescent | 12 | 0.45±0.34 |  |
|  | progressive | 0^a^ |  |  |  | progressive | 3 | 0.82±0.66 |  |
| CD28^+^CTLA-4^+^T^4+^/Lym | de novo / quiescent | 13 | 0.04±0.10 |  | CD28^+^CTLA-4^+^T^4+^/T^4+^ | de novo / quiescent | 11 | 0.36±0.29 |  |
|  | progressive | 4 | 0.01±0.01 |  |  | progressive | 4 | 0.54±0.41 |  |
| CD28^+^CTLA-4^+^T^4-^/Lym | de novo / quiescent | 13 | 0.03±0.03 |  |  |  |  |  |  |
|  | progressive | 4 | 0.01±0.01 |  |  |  |  |  |  |
| PD-1^+^T/Lym | de novo / quiescent | 13 | 1.32±2.13 |  | PD-1^+^T/T^3+^ | de novo / quiescent | 12 | 21.16±10.71 |  |
|  | progressive | 4 | 0.94±1.09 |  |  | progressive | 3 | 10.96±7.38 |  |
| PD-1^+^T^4+^/Lym | de novo / quiescent | 13 | 0.17±0.19 |  | PD-1^+^T^4+^/T^4+^ | de novo / quiescent | 11 | 13.68±7.14 |  |
|  | progressive | 4 | 0.06±0.05 |  |  | progressive | 3 | 9.38±6.63 |  |
| PD-1^+^T^4-^/Lym | de novo / quiescent | 13 | 1.16±1.94 |  | PD-1^+^T^4-^/T^4-^ | de novo / quiescent | 12 | 84.38±9.53 |  |
|  | progressive | 4 | 0.88±1.04 |  |  | progressive | 3 | 90.63±6.63 |  |
| PD-L1^+^T/Lym | de novo / quiescent | 13 | 0.69±0.96 |  | PD-L1^+^T/T^3+^ | de novo / quiescent | 12 | 11.71±5.69 |  |
|  | progressive | 4 | 0.68±0.86 |  |  | progressive | 3 | 10.67±4.30 |  |
| PD-L1^+^T^4+^/Lym | de novo / quiescent | 13 | 0.42±0.55 |  | PD-L1^+^T^4+^/T^4+^ | de novo / quiescent | 12 | 64.53±19.87 | Significant (**) |
|  | progressive | 4 | 0.40±0.56 |  |  | progressive | 2 | 47.25±2.47 |  |
| PD-L1^+^T^4-^/Lym | de novo / quiescent | 13 | 0.27±0.48 |  | PD-L1^+^T^4-^/T^4-^ | de novo / quiescent | 12 | 35.46±19.87 |  |
|  | progressive | 4 | 0.28±0.31 |  |  | progressive | 1 | 54.50 |  |
| LAG3^+^T/Lym | de novo / quiescent | 2 | 0.11±0.05 |  | LAG3^+^T/T^3+^ | de novo / quiescent | 0^a^ |  |  |
|  | progressive | 0^a^ |  |  |  | progressive | 0^a^ |  |  |
| LAG3^+^T^4+^/Lym | de novo / quiescent | 13 | 0.02±0.02 |  | LAG3^+^T^4+^/T^4+^ | de novo / quiescent | 12 | 0.77±0.52 |  |
|  | progressive | 4 | 0.03±0.01 |  |  | progressive | 3 | 1.06±0.19 |  |
| LAG3^+^T^4-^/Lym | de novo / quiescent | 13 | 0.06±0.09 |  | LAG3^+^T^4-^/T^4-^ | de novo / quiescent | 12 | 0.83±0.59 | Significant (**) |
|  | progressive | 4 | 0.03±0.03 |  |  | progressive | 4 | 1.46±1.02 |  |
| 2B4^+^T/Lym | de novo / quiescent | 8 | 2.95±2.90 |  | 2B4^+^T/T^3+^ | de novo / quiescent | 8 | 37.43±15.67 |  |
|  | progressive | 2 | 0.81±0.47 |  |  | progressive | 2 | 33.30±5.66 |  |
| 2B4^+^T^4+^/Lym | de novo / quiescent | 13 | 0.16±0.33 |  | 2B4^+^T^4+^/T^4+^ | de novo / quiescent | 11 | 1.62±1.25 | Highly significant (***) |
|  | progressive | 4 | 0.04±0.04 |  |  | progressive | 4 | 7.19±11.25 |  |
| 2B4^+^T^4-^/Lym | de novo / quiescent | 11 | 1.14±1.31 | significant (**) | 2B4^+^T^4-^/T^4-^ | de novo / quiescent | 10 | 42.46±20.09 |  |
|  | progressive | 4 | 0.40±0.50 |  |  | progressive | 2 | 46.95±2.90 |  |
| TIM3^+^T/Lym | de novo / quiescent | 10 | 0.36±0.20 | significant (**) | TIM3^+^T/T^3+^ | de novo / quiescent | 9 | 5.22±4.91 |  |
|  | progressive | 2 | 0.21±0.04 |  |  | progressive | 1 | 13.50 |  |
| TIM3^+^T^4+^/Lym | de novo / quiescent | 12 | 0.05±0.04 | significant (**) | TIM3^+^T^4+^/T^4+^ | de novo / quiescent | 11 | 1.65±1.00 |  |
|  | progressive | 5 | 0.01±0.01 |  |  | progressive | 4 | 1.50±0.86 |  |
| TIM3^+^T^4-^/Lym | de novo / quiescent | 12 | 0.26±0.18 | significant (**) | TIM3^+^T^4-^/T^4-^ | de novo / quiescent | 12 | 5.30±5.09 |  |
|  | progressive | 5 | 0.12±0.08 |  |  | progressive | 3 | 9.02±7.71 |  |
| KLRG1^+^T/Lym | de novo / quiescent | 13 | 1.96±2.55 | borderline significant (**) | KLRG1^+^T/T^3+^ | de novo / quiescent | 11 | 34.92±17.36 |  |
|  | progressive | 3 | 0.30±0.40 |  |  | progressive | 2 | 37.00±31.11 |  |
| KLRG1^+^T^4+^/Lym | de novo / quiescent | 13 | 0.23±0.43 |  | KLRG1^+^T^4+^/T^4+^ | de novo / quiescent | 11 | 12.76±11.34 |  |
|  | progressive | 4 | 0.33±0.53 |  |  | progressive | 4 | 13.75±10.44 |  |
| KLRG1^+^T^4-^/Lym | de novo / quiescent | 13 | 1.74±2.32 |  | KLRG1^+^T^4-^/T^4-^ | de novo / quiescent | 12 | 84.21±15.09 |  |
|  | progressive | 4 | 1.90±3.35 |  |  | progressive | 4 | 86.25±10.44 |  |
| TIGIT^+^T/Lym | de novo / quiescent | 11 | 1.36±1.63 |  | TIGIT^+^T/T^3+^ | de novo / quiescent | 11 | 35.64±14.49 |  |
|  | progressive | 4 | 2.34±2.54 |  |  | progressive | 4 | 31.05±8.67 |  |
| TIGIT^+^T^4+^/Lym | de novo / quiescent | 13 | 0.70±0.86 |  | TIGIT^+^T^4+^/T^4+^ | de novo / quiescent | 12 | 64.54±27.58 |  |
|  | progressive | 4 | 0.39±0.42 |  |  | progressive | 3 | 75.40±15.39 |  |
| TIGIT^+^T^4-^/Lym | de novo / quiescent | 13 | 1.77±2.57 |  | TIGIT^+^T^4-^/T^4-^ | de novo / quiescent | 12 | 35.68±28.05 |  |
|  | progressive | 4 | 1.95±2.17 |  |  | progressive | 3 | 24.60±15.39 |  |
| B/Lym | de novo / quiescent | 11 | 0.03±0.04 |  |  |  |  |  |  |
|  | progressive | 4 | 0.07±0.10 |  |  |  |  |  |  |
| B_nai_/Lym | de novo / quiescent | 11 | 2.81±4.03 |  | B_nai_/B | de novo / quiescent | 9 | 63.61±22.72 |  |
|  | progressive | 3 | 7.82±10.92 |  |  | progressive | 3 | 53.30±34.57 |  |
| B_mem(class)_/Lym | de novo / quiescent | 11 | 0.26±0.24 |  | B_mem(class)/_B | de novo / quiescent | 11 | 14.87±11.79 |  |
|  | progressive | 3 | 1.05±0.81 |  |  | progressive | 3 | 19.64±14.07 |  |
| B_mem_/Lym | de novo / quiescent | 11 | 0.11±0.13 |  | B_mem_/B | de novo / quiescent | 11 | 4.08±3.70 |  |
|  | progressive | 4 | 0.35±0.50 |  |  | progressive | 4 | 5.46±6.88 |  |
| B_reg_/Lym | de novo / quiescent | 0^a^ |  |  | B_reg_/B | de novo / quiescent | 11 | 2.21±2.23 | borderline significant (*) |
|  | progressive | 0^a^ |  |  |  | progressive | 4 | 0.66±0.90 |  |
| Innate cells | | | | | | | | | |
| In Lym-Gate | | | | | In subsets | | | | |
| De novo/quiescent vs progressive |  | N | % Mean  SD | *p* | De novo/quiescent vs progressive |  | N | % Mean  SD | *p* |
| NK^56+^/Lym | de novo / quiescent | 12 | 0.05±0.06 |  |  |  |  |  |  |
|  | progressive | 4 | 0.09±0.07 |  |  |  |  |  |  |
| NK^56bri^/Lym | de novo / quiescent | 10 | 1.35±2.62 |  | NK^56bri^/NK^56+^ | de novo / quiescent | 11 | 13.88±13.29 |  |
|  | progressive | 4 | 2.65±3.49 |  |  | progressive | 3 | 18.48±14.79 |  |
| NK^56bri16-^/Lym | de novo / quiescent | 11 | 1.48±3.05 |  | NK^56bri16-^/NK^56+^ | de novo / quiescent | 11 | 3.97±3.43 | significant (**) |
|  | progressive | 4 | 1.93±2.84 |  |  | progressive | 4 | 17.28±15.13 |  |
| NK^56bri16+^/Lym | de novo / quiescent | 11 | 2.25±5.30 |  | NK^56bri16+^/NK^56+^ | de novo / quiescent | 10 | 6.84±5.86 |  |
|  | progressive | 4 | 0.72±0.75 |  |  | progressive | 4 | 8.10±4.72 |  |
| NK^56dim16+^/Lym | de novo / quiescent | 11 | 14.04±26.61 |  | NK^56dim16+^/NK^56+^ | de novo / quiescent | 10 | 71.73±20.74 |  |
|  | progressive | 4 | 4.56±2.81 |  |  | progressive | 4 | 61.18±20.51 |  |
| NK^56bri57-^/Lym | de novo / quiescent | 10 | 1.42±2.67 |  | NK^56bri57-^/NK^56+^ | de novo / quiescent | 11 | 14.37±13.72 |  |
|  | progressive | 4 | 2.57±3.40 |  |  | progressive | 3 | 17.85±14.79 |  |
| NK^56dim57-^/Lym | de novo / quiescent | 9 | 2.97±4.64 |  | NK^56dim57-^/NK^56+^ | de novo / quiescent | 11 | 50.22±17.09 |  |
|  | progressive | 4 | 3.36±2.90 |  |  | progressive | 3 | 43.40±12.40 |  |
| NK^56dim57+^/Lym | de novo / quiescent | 11 | 3.27±5.08 |  | NK^56dim57+^/NK^56+^ | de novo / quiescent | 10 | 26.43±19.68 | highly significant (***) |
|  | progressive | 4 | 2.71±1.82 |  |  | progressive | 3 | 23.13±1.10 |  |
| NK^56dim57-16bri^/Lym | de novo / quiescent | 11 | 5.92±10.04 | borderline significant (*) | NK^56dim57-16bri^/NK^56+^ | de novo / quiescent | 11 | 24.18±13.57 |  |
|  | progressive | 4 | 1.68±1.37 |  |  | progressive | 4 | 24.08±12.35 |  |
| NK^56dim57+16+^/Lym | de novo / quiescent | 11 | 3.26±5.07 |  | NK^56dim57+16+^/NK^56+^ | de novo / quiescent | 10 | 26.36±19.69 |  |
|  | progressive | 4 | 2.70±1.82 |  |  | progressive | 4 | 35.00±24.02 |  |
| CTLA-4^+^NK^56+^/Lym | de novo / quiescent | 12 | 1.30±1.28 |  | CTLA-4^+^NK^56+^/NK^56+^ | de novo / quiescent | 10 | 32.13±13.87 |  |
|  | progressive | 5 | 0.64±0.88 |  |  | progressive | 5 | 35.78±18.49 |  |
| PD-1^+^NK^56+^/Lym | de novo / quiescent | 13 | 0.05±0.08 |  | PD-1^+^NK^56+^/NK^56+^ | de novo / quiescent | 13 | 11.39±9.21 |  |
|  | progressive | 5 | 0.06±0.06 |  |  | progressive | 4 | 19.95±17.68 |  |
| PD-L1^+^NK^56+^/Lym | de novo / quiescent | 12 | 0.06±0.09 |  | PD-L1^+^NK^56+^/NK^56+^ | de novo / quiescent | 12 | 14.68±11.79 | borderline significant (*) |
|  | progressive | 5 | 0.06±0.05 |  |  | progressive | 4 | 23.40±19.23 |  |
| PD-L2^+^NK^56+^/Lym | de novo / quiescent | 13 | 0.05±0.05 |  | PD-L2^+^NK^56+^/NK^56+^ | de novo / quiescent | 12 | 0.81±0.69 |  |
|  | progressive | 4 | 0.07±0.10 |  |  | progressive | 5 | 2.14±1.12 |  |
| CD40L^+^NK^56+^/Lym | de novo / quiescent | 13 | 0.09±0.13 |  | CD40L^+^NK^56+^/NK^56+^ | de novo / quiescent | 12 | 15.98±14.65 |  |
|  | progressive | 5 | 0.10±0.10 |  |  | progressive | 5 | 17.03±15.56 |  |
| LAG3^+^NK^56+^/Lym | de novo / quiescent | 13 | 0.03±0.03 |  | LAG3^+^NK^56+^/NK^56+^ | de novo / quiescent | 12 | 90.38±8.78 |  |
|  | progressive | 4 | 0.06±0.05 |  |  | progressive | 3 | 83.60±14.64 |  |
| 2B4^+^NK^56+^/Lym | de novo / quiescent | 11 | 3.71±2.97 |  | 2B4^+^NK^56+^/NK^56+^ | de novo / quiescent | 10 | 65.92±20.67 |  |
|  | progressive | 4 | 1.27±1.00 |  |  | progressive | 4 | 64.58±15.56 |  |
| TIM3^+^NK^56+^/Lym | de novo / quiescent | 11 | 1.75±1.35 |  |  |  |  |  |  |
|  | progressive | 4 | 1.16±0.89 |  |  |  |  |  |  |
| KLRG1^+^NK^56+^/Lym | de novo / quiescent | 13 | 1.09±1.05 |  | KLRG1^+^NK^56+^/NK^56+^ | de novo / quiescent | 12 | 21.78±12.46 |  |
|  | progressive | 5 | 1.28±1.37 |  |  | progressive | 5 | 26.96±19.90 |  |
| TIGIT^+^NK^56+^/Lym | de novo / quiescent | 12 | 2.03±1.67 |  | TIGIT^+^NK^56+^/NK^56+^ | de novo / quiescent | 10 | 31.43±10.19 | borderline significant (*) |
|  | progressive | 5 | 1.10±1.56 |  |  | progressive | 5 | 29.52±20.81 |  |
| CIK^56+^/Lym | de novo / quiescent | 13 | 0.18±0.21 |  |  |  |  |  |  |
|  | progressive | 5 | 0.81±0.30 |  |  |  |  |  |  |
| CD40L^+^CIK^56+^/Lym | de novo / quiescent | 13 | 0.25±0.30 |  | CD40L^+^CIK^56+^/CIK^56+^ | de novo / quiescent | 12 | 9.90±12.11 |  |
|  | progressive | 5 | 0.17±0.21 |  |  | progressive | 5 | 10.00±11.13 |  |
| MAIT cells /Lym | de novo / quiescent | 12 | 0.01±0.01 | significant (**) |  |  |  |  |  |
|  | progressive | 5 | 0.00±0.00 |  |  |  |  |  |  |
| CTLA-4^+^CIK^56+^/Lym | de novo / quiescent | 13 | 0.02±0.01 | significant (**) | CTLA-4^+^CIK^56+^/CIK^56+^ | de novo / quiescent | 11 | 17.56±12.13 | borderline significant (*) |
|  | progressive | 5 | 0.10±0.03 |  |  | progressive | 5 | 12.75±4.87 |  |
| PD-1^+^CIK^56+^/Lym | de novo / quiescent | 13 | 0.21±0.28 | significant (**) | PD-1^+^CIK^56+^/CIK^56+^ | de novo / quiescent | 12 | 5.50±4.86 |  |
|  | progressive | 5 | 0.03±0.02 |  |  | progressive | 5 | 4.47±2.59 |  |
| PD-L1^+^CIK^56+^/Lym | de novo / quiescent | 13 | 0.32±0.35 | significant (**) | PD-L1^+^CIK^56+^/CIK^56+^ | de novo / quiescent | 10 | 6.95±5.29 | significant (**) |
|  | progressive | 5 | 0.12±0.11 |  |  | progressive | 5 | 16.21±12.44 |  |
| PD-L2^+^CIK^56+^/Lym | de novo / quiescent | 13 | 0.01±0.01 | highly significant (***) | PD-L2^+^CIK^56+^/CIK^56+^ | de novo / quiescent | 12 | 9.08±10.75 |  |
|  | progressive | 5 | 0.16±0.26 |  |  | progressive | 5 | 10.86±9.09 |  |
| KLRG1^+^CIK^56+^/Lym | de novo / quiescent | 13 | 0.10±0.13 | highly significant (***) | KLRG1^+^CIK^56+^/CIK^56+^ | de novo / quiescent | 11 | 62.41±24.06 |  |
|  | progressive | 5 | 0.37±0.68 |  |  | progressive | 4 | 52.33±31.84 |  |
| TIGIT^+^CIK^56+^/Lym | de novo / quiescent | 13 | 0.09±0.16 | significant (**) | TIGIT^+^CIK^56+^/CIK^56+^ | de novo / quiescent | 12 | 34.20±13.43 |  |
|  | progressive | 5 | 0.29±0.48 |  |  | progressive | 2 | 30.95±26.94 |  |
| iNKT/Lym | de novo / quiescent | 12 | 0.21±0.41 |  |  |  |  |  |  |
|  | progressive | 4 | 0.24±0.05 |  |  |  |  |  |  |
| iNKT^3+^/Lym | de novo / quiescent | 12 | 0.10±0.31 |  |  |  |  |  |  |
|  | progressive | 4 | 0.02±0.02 |  |  |  |  |  |  |
| iNKT^161+^/Lym | de novo / quiescent | 12 | 0.10±0.16 |  |  |  |  |  |  |
|  | progressive | 4 | 0.15±0.05 |  |  |  |  |  |  |

# **Supplementary Table 4 — Chronic GvHD: number of organs affected**

| Myeloid cells | | | | | | | | | | |
| --- | --- | --- | --- | --- | --- | --- | --- | --- | --- | --- |
| In non Lym-gate | | | | | In subsets | | | | | |
| Number of organs affected by cGvHD | 1 vs. > 1 | N | % Mean  SD | p | Number of organs affected by cGvHD | 1 vs. > 1 | N | % Mean  SD | | *p* |
| CD14^+^/non Lym | 1 organ | 4 | 3.32±2.17 | borderline significant (*) |  |  |  |  | |  |
|  | more than 1 organ | 10 | 8.49±6.81 |  |  |  |  |  | |  |
| CTLA-4^+^Mo/non Lym | 1 organ | 4 | 0.86±0.55 | borderline significant (*) | CTLA-4^+^Mo/Mo | 1 organ | 3 | 18.50±4.10 | |  |
|  | more than 1 organ | 11 | 1.97±2.31 |  |  | more than 1 organ | 11 | 16.92±5.99 | |  |
| PD-1^+^Mo/non Lym | 1 organ | 3 | 2.00±2.56 | significant (**) | PD-1^+^Mo/Mo | 1 organ | 3 | 20.82±22.67 | | Highly significant (***) |
|  | more than 1 organ | 12 | 0.97±0.98 |  |  | more than 1 organ | 12 | 12.37±7.14 | |  |
| PD-L1^+^Mo/non Lym | 1 organ | 4 | 6.34±2.82 |  | PD-L1^+^Mo/Mo | 1 organ | 3 | 91.90±6.33 | |  |
|  | more than 1 organ | 11 | 4.17±2.46 |  |  | more than 1 organ | 10 | 87.90±11.79 | |  |
| LAG3^+^Mo/non Lym | 1 organ | 4 | 0.11±0.03 |  | LAG3^+^Mo/Mo | 1 organ | 4 | 1.16±0.49 | | borderline significant (*) |
|  | more than 1 organ | 12 | 0.10±0.04 |  |  | more than 1 organ | 12 | 1.72±1.06 | |  |
| 2B4^+^Mo/non Lym | 1 organ | 3 | 7.91±2.43 |  | 2B4^+^Mo/Mo | 1 organ | 3 | 99.57±0.32 | |  |
|  | more than 1 organ | 10 | 5.25±2.13 |  |  | more than 1 organ | 12 | 95.38±8.38 | |  |
| Tim3^+^Mo/non Lym | 1 organ | 3 | 5.86±2.22 |  | Tim3^+^Mo/Mo | 1 organ | 3 | 92.83±3.75 | |  |
|  | more than 1 organ | 10 | 4.52±2.40 |  |  | more than 1 organ | 11 | 88.44±7.39 | |  |
| DC^80+^/non Lym | 1 organ | 4 | 0.78±0.21 | borderline significant (*) |  |  |  |  | |  |
|  | more than 1 organ | 12 | 1.37±0.74 |  |  |  |  |  | |  |
| DC_mat_^80+^/non Lym | 1 organ | 4 | 0.25±0.23 |  | DC_mat_^80+^/DC^80+^ | 1 organ | 3 | 21.17±7.99 | |  |
|  | more than 1 organ | 12 | 0.33±0.22 |  |  | more than 1 organ | 12 | 25.62±12.72 | |  |
| DC_tol_^80+^/non Lym | 1 organ | 4 | 0.27±0.23 |  | DC_tol_^80+^/DC^80+^ | 1 organ | 4 | 20.43±11.82 | |  |
|  | more than 1 organ | 12 | 0.48±0.39 |  |  | more than 1 organ | 8 | 32.64±16.16 | |  |
| CTLA-4^+^DC^80+^/non Lym | 1 organ | 4 | 0.22±0.19 |  | CTLA-4^+^DC^80+^/DC^80+^ | 1 organ | 4 | 31.18±15.62 | |  |
|  | more than 1 organ | 12 | 0.41±0.30 |  |  | more than 1 organ | 9 | 24.01±13.20 | |  |
| CTLA-4^+^DC_mat_^80+^/non Lym | 1 organ | 4 | 0.16±0.16 |  | CTLA-4^+^DC_mat_^80+^/DC_mat_^80+^ | 1 organ | 4 | 58.93±14.87 | |  |
|  | more than 1 organ | 12 | 0.23±0.17 |  |  | more than 1 organ | 12 | 74.68±18.52 | |  |
| PMN-MDSC/non Lym | 1 organ | 3 | 4.67±3.68 | significant (**) | PMN-MDSC/CD11b+CD33^+^ | 1 organ | 4 | 74.70±13.46 | |  |
|  | more than 1 organ | 12 | 20.45±15.26 |  |  | more than 1 organ | 11 | 65.61±20.00 | |  |
| M-MDSC/non Lym | 1 organ | 4 | 3.05±3.03 |  | M-MDSC/CD11b+CD33^+^ | 1 organ | 4 | 25.30±13.46 | |  |
|  | more than 1 organ | 10 | 8.84±7.08 |  |  | more than 1 organ | 9 | 27.17±12.92 | |  |
| CTLA-4^+^PMN-MDSC/non Lym | 1 organ | 3 | 1.01±0.72 |  | CTLA-4^+^PMN-MDSC/PMN-MDSC | 1 organ | 3 | 15.78±10.54 | |  |
|  | more than 1 organ | 12 | 1.60±2.04 |  |  | more than 1 organ | 12 | 12.04±12.05 | |  |
| CTLA-4^+^M-MDSC/non Lym | 1 organ | 4 | 1.11±0.76 | significant (**) | CTLA-4^+^M-MDSC/PMN-MDSC | 1 organ | 2 | 31.50±10.18 | |  |
|  | more than 1 organ | 12 | 3.37±4.13 |  |  | more than 1 organ | 11 | 20.79±7.08 | |  |
| In non Lym-Gate | | | | | In subsets | | | | | |
| Number of organs affected by cGvHD | 1 vs. > 1 | N | % Mean  SD | p | Number of organs affected by cGvHD | 1 vs. > 1 | N | % Mean | SD | *p* |
| Adaptive cells | | | | | | | | | | |
| T/Lym | 1 organ | 4 | 0.04±0.03 |  |  |  |  |  | |  |
|  | more than 1 organ | 10 | 0.04±0.04 |  |  |  |  |  | |  |
| T^4+^/Lym | 1 organ | 4 | 1.16±0.94 |  | T^4+^/T^3+^ | 1 organ | 3 | 34.03±25.78 | | significant (**) |
|  | more than 1 organ | 10 | 1.13±0.91 |  |  | more than 1 organ | 8 | 29.51±9.98 | |  |
| T^4-^/Lym | 1 organ | 4 | 2.37±2.58 |  | T^4-^/T^3+^ | 1 organ | 3 | 65.50±26.57 | | borderline significant (*) |
|  | more than 1 organ | 9 | 2.19±2.18 |  |  | more than 1 organ | 9 | 67.92±12.10 | |  |
| T_prol-early_ /Lym | 1 organ | 4 | 0.20±0.20 |  | T_prol-early_ /T^3+^ | 1 organ | 3 | 7.36±9.83 | | significant (**) |
|  | more than 1 organ | 10 | 0.46±0.34 |  |  | more than 1 organ | 10 | 8.41±4.33 | |  |
| T_prol-late_ /Lym | 1 organ | 4 | 0.58±0.58 |  | T_prol-late_ /T^3+^ | 1 organ | 2 | 12.81±13.85 | |  |
|  | more than 1 organ | 10 | 0.44±0.34 |  |  | more than 1 organ | 10 | 9.30±7.50 | |  |
| T_nn_ /Lym | 1 organ | 4 | 2.13±1.33 |  | T_nn_ /T^3+^ | 1 organ | 4 | 67.45±15.56 | |  |
|  | more than 1 organ | 10 | 1.71±1.61 |  |  | more than 1 organ | 9 | 51.20±15.17 | |  |
| T_em_ /Lym | 1 organ | 4 | 2.07±1.36 |  | T_em_ /T^3+^ | 1 organ | 4 | 64.53±15.43 | |  |
|  | more than 1 organ | 10 | 1.64±1.58 |  |  | more than 1 organ | 8 | 51.96±14.18 | |  |
| T_cm_ /Lym | 1 organ | 4 | 0.06±0.06 |  | T_cm_ /T^3+^ | 1 organ | 3 | 1.52±1.51 | |  |
|  | more than 1 organ | 10 | 0.06±0.07 |  |  | more than 1 organ | 10 | 1.63±1.41 | |  |
| CD28^+^T/Lym | 1 organ | 4 | 1.66±1.13 |  | CD28^+^T/T^3+^ | 1 organ | 2 | 72.25±28.50 | |  |
|  | more than 1 organ | 9 | 1.96±1.65 |  |  | more than 1 organ | 9 | 65.30±19.17 | |  |
| CD28^+^T^4+^/Lym | 1 organ | 4 | 1.06±0.83 |  | CD28^+^T^4+^/T^4+^ | 1 organ | 3 | 89.50±15.18 | |  |
|  | more than 1 organ | 10 | 1.63±2.23 |  |  | more than 1 organ | 10 | 91.44±9.81 | |  |
| CD28^+^T^4-^/Lym | 1 organ | 4 | 0.59±0.49 |  | CD28^+^T^4-^/T^4-^ | 1 organ | 2 | 56.80±24.32 | |  |
|  | more than 1 organ | 8 | 0.74±0.51 |  |  | more than 1 organ | 11 | 43.81±25.02 | |  |
| CD40L^+^T^4+^/Lym | 1 organ | 4 | 0.16±0.20 |  | CD40L^+^T/T^3+^ | 1 organ | 1 | 10.40 | |  |
|  | more than 1 organ | 9 | 0.14±0.18 |  |  | more than 1 organ | 4 | 5.07±5.47 | |  |
| CD40L^+^T^4-^/Lym | 1 organ | 4 | 0.02±0.01 | borderline significant (*) | CD40L^+^T^4+^/T^4+^ | 1 organ | 4 | 10.14±10.77 | |  |
|  | more than 1 organ | 9 | 0.09±0.12 |  |  | more than 1 organ | 7 | 6.81±7.24 | |  |
|  |  |  |  |  | CD40L^+^T^4-^/T^4-^ | 1 organ | 4 | 0.90±0.66 | | highly significant (***) |
|  |  |  |  |  |  | more than 1 organ | 8 | 2.01  ±1.98 | |  |
| T^H1/Gui^/Lym | 1 organ | 4 | 1.28±1.77 |  | T^H1/Gui^/T^4+^ | 1 organ | 4 | 15.69±9.09 | |  |
|  | more than 1 organ | 11 | 0.49±0.91 |  |  | more than 1 organ | 10 | 11.48±7.75 | |  |
| T^H2/Gui^/Lym | 1 organ | 4 | 0.35±0.17 |  | T^H2/Gui^/T^4+^ | 1 organ | 4 | 8.31±1.88 | |  |
|  | more than 1 organ | 12 | 0.37±0.60 |  |  | more than 1 organ | 11 | 7.45±3.97 | |  |
| T^H17/Gui^/Lym | 1 organ | 4 | 0.19±0.16 |  | T^H17/Gui^/T^4+^ | 1 organ | 4 | 8.58±4.76 | |  |
|  | more than 1 organ | 12 | 0.53±0.94 |  |  | more than 1 organ | 9 | 5.47±3.65 | |  |
| γδ^+^T/Lym | 1 organ | 4 | 4.32±5.29 |  | γδ^+^T/T^3+^ | 1 organ | 4 | 4.32±5.29 | |  |
|  | more than 1 organ | 8 | 6.52±3.76 |  |  | more than 1 organ | 8 | 6.52±3.76 | |  |
| γδ^+^T^4+^/Lym | 1 organ | 4 | 0.03±0.04 |  | γδ^+^T^4+^/T^3+^ | 1 organ | 4 | 0.50±0.26 | | borderline significant (*) |
|  | more than 1 organ | 9 | 0.03±0.02 |  |  | more than 1 organ | 9 | 1.07±0.91 | |  |
| γδ^+^T^4-^/Lym | 1 organ | 4 | 0.54±1.03 | significant (**) | γδ^+^T^4-^/T^3+^ | 1 organ | 4 | 3.82±5.24 | |  |
|  | more than 1 organ | 9 | 0.32±0.42 |  |  | more than 1 organ | 8 | 5.42±3.60 | |  |
| Intß7^+^T/Lym | 1 organ | 4 | 0.93±0.54 |  | Intß7^+^T/T^3+^ | 1 organ | 4 | 27.64±17.75 | |  |
|  | more than 1 organ | 9 | 1.72±1.49 |  |  | more than 1 organ | 8 | 36.08±12.74 | |  |
| Intß7^+^T^4+^/Lym | 1 organ | 4 | 0.57±1.07 | significant (**) | Intß7^+^T^4+^/T^3+^ | 1 organ | 4 | 11.62±8.92 | |  |
|  | more than 1 organ | 9 | 0.35±0.41 |  |  | more than 1 organ | 7 | 14.73±5.03 | |  |
| Intß7^+^T^4-^/Lym | 1 organ | 4 | 0.56±0.38 | borderline significant (*) | Intß7^+^T^4-^/T^3+^ | 1 organ | 2 | 26.30±12.02 | |  |
|  | more than 1 organ | 9 | 1.30±1.21 |  |  | more than 1 organ | 8 | 25.13±8.50 | |  |
| CD127^+^T/Lym | 1 organ | 4 | 2.36±2.02 |  | CD127^+^T/T^3+^ | 1 organ | 3 | 61.43±24.46 | |  |
|  | more than 1 organ | 11 | 2.46±2.32 |  |  | more than 1 organ | 8 | 56.41±21.35 | |  |
|  |  |  |  |  | T^4+^_reg_ /T^4+^ | 1 organ | 4 | 11.47±3.95 | |  |
|  |  |  |  |  |  | more than 1 organ | 7 | 6.55±4.02 | |  |
| T^4+^_reg_/Lym | 1 organ | 4 | 0.15±0.07 |  | T^4+^_reg_ /T^3+^ | 1 organ | 4 | 4.70±3.11 | |  |
|  | more than 1 organ | 9 | 0.12±0.10 |  |  | more than 1 organ | 9 | 3.27±2.45 | |  |
|  |  |  |  |  | T^4-^_reg_ /T^4-^ | 1 organ | 4 | 1.36±1.33 | |  |
|  |  |  |  |  |  | more than 1 organ | 9 | 1.00±1.63 | |  |
| T^4-^_reg_/Lym | 1 organ | 4 | 0.11±0.21 | highly significant (***) | T^4-^_reg_ /T^3+^ | 1 organ | 4 | 0.73±0.76 | |  |
|  | more than 1 organ | 9 | 0.02±0.02 |  |  | more than 1 organ | 9 | 0.53±0.71 | |  |
| CTLA-4^+^T/Lym | 1 organ | 0a |  |  | CTLA-4^+^T/T^3+^ | 1 organ | 0^a^ |  | |  |
|  | more than 1 organ | 1 | 0.20 |  |  | more than 1 organ | 1 | 0.92 | |  |
| CTLA-4^+^T^4+^/Lym | 1 organ | 4 | 0.01±0.01 |  | CTLA-4^+^T^4+^/T^4+^ | 1 organ | 4 | 0.51±0.27 | |  |
|  | more than 1 organ | 11 | 0.02±0.02 |  |  | more than 1 organ | 11 | 0.79±0.71 | |  |
| CTLA-4^+^T^4-^/Lym | 1 organ | 4 | 0.03±0.03 |  | CTLA-4^+^T^4-^/T^4-^ | 1 organ | 4 | 0.94±1.07 | |  |
|  | more than 1 organ | 11 | 0.05±0.05 |  |  | more than 1 organ | 11 | 1.35±0.97 | |  |
| CD28^+^CTLA-4^+^T/Lym | 1 organ | 0a |  |  | CD28^+^CTLA-4^+^T^4-^/T^4-^ | 1 organ | 4 | 0.47±0.28 | |  |
|  | more than 1 organ | 1 | 0.11 |  |  | more than 1 organ | 10 | 0.59±0.48 | |  |
| CD28^+^CTLA-4^+^T^4+^/Lym | 1 organ | 4 | 0.09±0.18 | highly significant (***) | CD28^+^CTLA-4^+^T^4+^/T^4+^ | 1 organ | 4 | 0.36±0.41 | |  |
|  | more than 1 organ | 11 | 0.01±0.02 |  |  | more than 1 organ | 9 | 0.50±0.26 | |  |
| CD28^+^CTLA-4^+^T^4-^/Lym | 1 organ | 4 | 0.01±0.01 |  |  |  |  |  | |  |
|  | more than 1 organ | 11 | 0.03±0.03 |  |  |  |  |  | |  |
| PD-1^+^T/Lym | 1 organ | 4 | 0.58±0.31 |  | PD-1^+^T/T^3+^ | 1 organ | 3 | 18.64±14.81 | |  |
|  | more than 1 organ | 11 | 1.54±2.34 |  |  | more than 1 organ | 10 | 17.97±10.84 | |  |
| PD-1^+^T^4+^/Lym | 1 organ | 4 | 0.09±0.07 |  | PD-1^+^T^4+^/T^4+^ | 1 organ | 3 | 13.39±12.05 | |  |
|  | more than 1 organ | 11 | 0.16±0.21 |  |  | more than 1 organ | 9 | 11.68±6.19 | |  |
| PD-1^+^T^4-^/Lym | 1 organ | 4 | 0.49±0.32 |  | PD-1^+^T^4-^/T^4-^ | 1 organ | 3 | 86.60±12.04 | |  |
|  | more than 1 organ | 11 | 1.38±2.14 |  |  | more than 1 organ | 10 | 85.81±9.86 | |  |
| PD-L1^+^T/Lym | 1 organ | 4 | 0.31±0.31 |  | PD-L1^+^T/T^3+^ | 1 organ | 4 | 8.32±3.66 | |  |
|  | more than 1 organ | 11 | 0.87±1.08 |  |  | more than 1 organ | 9 | 12.86±4.50 | |  |
| PD-L1^+^T^4+^/Lym | 1 organ | 4 | 0.14±0.13 | significant (**) | PD-L1^+^T^4+^/T^4+^ | 1 organ | 4 | 57.15±18.95 | |  |
|  | more than 1 organ | 11 | 0.54±0.63 |  |  | more than 1 organ | 8 | 60.33±21.02 | |  |
| PD-L1^+^T^4-^/Lym | 1 organ | 4 | 0.17±0.18 |  | PD-L1^+^T^4-^/T^4-^ | 1 organ | 4 | 42.85±18.95 | |  |
|  | more than 1 organ | 11 | 0.34±0.53 |  |  | more than 1 organ | 7 | 38.05±22.17 | |  |
| LAG3^+^T/Lym | 1 organ | 0a |  |  | LAG3^+^T/T^3+^ | 1 organ | 0^a^ |  | |  |
|  | more than 1 organ | 1 | 0.14 |  |  | more than 1 organ | 0^a^ |  | |  |
| LAG3^+^T^4+^/Lym | 1 organ | 4 | 0.02±0.01 |  | LAG3^+^T^4+^/T^4+^ | 1 organ | 4 | 0.90±0.32 | |  |
|  | more than 1 organ | 11 | 0.03±0.02 |  |  | more than 1 organ | 10 | 0.86±0.53 | |  |
| LAG3^+^T^4-^/Lym | 1 organ | 4 | 0.06±0.09 |  | LAG3^+^T^4-^/T^4-^ | 1 organ | 4 | 0.98±0.34 | | borderline significant (*) |
|  | more than 1 organ | 11 | 0.06±0.08 |  |  | more than 1 organ | 10 | 1.04±0.87 | |  |
| 2B4^+^T/Lym | 1 organ | 2 | 6.08±5.11 | highly significant (***) | 2B4^+^T/T^3+^ | 1 organ | 2 | 52.30±12.59 | |  |
|  | more than 1 organ | 7 | 1.60±1.21 |  |  | more than 1 organ | 6 | 27.33±7.61 | |  |
| 2B4^+^T^4+^/Lym | 1 organ | 4 | 0.05±0.05 |  | 2B4^+^T^4+^/T^4+^ | 1 organ | 3 | 1.31±0.99 | |  |
|  | more than 1 organ | 11 | 0.14±0.35 |  |  | more than 1 organ | 10 | 3.56±7.23 | |  |
| 2B4^+^T^4-^/Lym | 1 organ | 3 | 0.81±1.34 |  | 2B4^+^T^4-^/T^4-^ | 1 organ | 2 | 63.05±20.86 | |  |
|  | more than 1 organ | 11 | 0.90±1.23 |  |  | more than 1 organ | 8 | 34.30±13.73 | |  |
| TIM3^+^T/Lym | 1 organ | 2 | 0.31±0.22 |  | TIM3^+^T/T^3+^ | 1 organ | 2 | 8.14±8.86 | |  |
|  | more than 1 organ | 9 | 0.33±0.20 |  |  | more than 1 organ | 6 | 5.29±4.36 | |  |
| TIM3^+^T^4+^/Lym | 1 organ | 4 | 0.03±0.03 |  | TIM3^+^T^4+^/T^4+^ | 1 organ | 3 | 1.39±0.74 | |  |
|  | more than 1 organ | 12 | 0.04±0.04 |  |  | more than 1 organ | 11 | 1.78±0.95 | |  |
| TIM3^+^T^4-^/Lym | 1 organ | 4 | 0.16±0.15 |  | TIM3^+^T^4-^/T^4-^ | 1 organ | 4 | 7.34±6.38 | |  |
|  | more than 1 organ | 12 | 0.22±0.17 |  |  | more than 1 organ | 9 | 5.23±5.10 | |  |
| KLRG1^+^T/Lym | 1 organ | 3 | 1.14±1.32 |  | KLRG1^+^T/T^3+^ | 1 organ | 4 | 46.60±18.92 | |  |
|  | more than 1 organ | 11 | 1.92±2.81 |  |  | more than 1 organ | 7 | 28.54  ±18.50 | |  |
| KLRG1^+^T^4+^/Lym | 1 organ | 4 | 0.33±0.53 |  | KLRG1^+^T^4+^/T^4+^ | 1 organ | 4 | 6.28±5.73 | | borderline significant (*) |
|  | more than 1 organ | 11 | 0.24±0.47 |  |  | more than 1 organ | 9 | 14.66±10.81 | |  |
| KLRG1^+^T^4-^/Lym | 1 organ | 4 | 2.54±3.08 |  | KLRG1^+^T^4-^/T^4-^ | 1 organ | 4 | 93.73±5.73 | |  |
|  | more than 1 organ | 11 | 1.68±2.55 |  |  | more than 1 organ | 10 | 81.89±14.94 | |  |
| TIGIT^+^T/Lym | 1 organ | 4 | 2.16±1.96 |  | TIGIT^+^T/T^3+^ | 1 organ | 3 | 50.70±15.27 | |  |
|  | more than 1 organ | 9 | 1.47±2.11 |  |  | more than 1 organ | 10 | 29.97±9.85 | |  |
| TIGIT^+^T^4+^/Lym | 1 organ | 4 | 0.84±1.08 |  | TIGIT^+^T^4+^/T^4+^ | 1 organ | 3 | 67.80±25.54 | |  |
|  | more than 1 organ | 11 | 0.61±0.76 |  |  | more than 1 organ | 10 | 64.72±29.01 | |  |
| TIGIT^+^T^4-^/Lym | 1 organ | 4 | 1.31±1.92 |  | TIGIT^+^T^4-^/T^4-^ | 1 organ | 4 | 47.40±36.86 | |  |
|  | more than 1 organ | 11 | 2.16±2.80 |  |  | more than 1 organ | 9 | 29.16±22.92 | |  |
| B/Lym | 1 organ | 4 | 0.08±0.10 |  |  |  |  |  | |  |
|  | more than 1 organ | 9 | 0.04±0.05 |  |  |  |  |  | |  |
| B_nai_/Lym | 1 organ | 4 | 7.00±9.44 |  | B_nai_/B | 1 organ | 3 | 73.93±29.39 | |  |
|  | more than 1 organ | 8 | 3.13±4.27 |  |  | more than 1 organ | 7 | 58.51±26.65 | |  |
| B_mem(class)_/Lym | 1 organ | 4 | 0.38±0.34 |  | B_mem(class)_/B | 1 organ | 4 | 14.15±17.10 | |  |
|  | more than 1 organ | 8 | 0.48±0.64 |  |  | more than 1 organ | 8 | 14.24±10.04 | |  |
| B_mem_/Lym | 1 organ | 4 | 0.14±0.14 |  | B_mem_/B | 1 organ | 4 | 2.91±3.09 | |  |
|  | more than 1 organ | 9 | 0.20±0.35 |  |  | more than 1 organ | 9 | 4.13±4.96 | |  |
|  |  |  |  |  | B_reg_/B | 1 organ | 4 | 2.39±2.95 | |  |
|  |  |  |  |  |  | more than 1 organ | 9 | 1.81±1.87 | |  |
| Innate cells | | | | | | | | | | |
| In Lym-Gate | | | | | In subsets | | | | | |
| Number of organs affected by cGvHD | 1 vs. > 1 | N | % Mean  SD | p | Number of organs affected by cGvHD | 1 vs. > 1 | N | % Mean  SD | | *p* |
| NK^56+^/Lym | 1 organ | 4 | 0.11±0.10 | highly significant (***) |  |  |  |  | |  |
|  | more than 1 organ | 10 | 0.04±0.04 |  |  |  |  |  | |  |
| NK^56bri^/Lym | 1 organ | 4 | 4.98±3.56 | highly significant (***) | NK^56bri^/NK^56+^ | 1 organ | 3 | 13.27±4.48 | | significant (**) |
|  | more than 1 organ | 8 | 0.52±0.90 |  |  | more than 1 organ | 9 | 17.93±15.21 | |  |
| NK^56bri16-^/Lym | 1 organ | 4 | 3.10±2.81 |  | NK^56bri16-^/NK^56+^ | 1 organ | 4 | 12.32±16.62 | | significant (**) |
|  | more than 1 organ | 9 | 1.29±3.15 |  |  | more than 1 organ | 9 | 6.80±6.29 | |  |
| NK^56bri16+^/Lym | 1 organ | 4 | 1.88±1.16 |  | NK^56bri16+^/NK^56+^ | 1 organ | 4 | 9.15±5.17 | |  |
|  | more than 1 organ | 9 | 2.23±5.89 |  |  | more than 1 organ | 8 | 7.46±5.74 | |  |
| NK^56dim16+^/Lym | 1 organ | 4 | 28.75±39.73 | significant (**) | NK^56dim16+^/NK^56+^ | 1 organ | 4 | 68.78±21.65 | |  |
|  | more than 1 organ | 9 | 5.83±11.11 |  |  | more than 1 organ | 8 | 62.85±19.54 | |  |
| NK^56bri57-^/Lym | 1 organ | 4 | 5.09±3.39 | highly significant (***) | NK^56bri57-^/NK^56+^ | 1 organ | 3 | 15.97±7.92 | | borderline significant (*) |
|  | more than 1 organ | 8 | 0.50±0.88 |  |  | more than 1 organ | 9 | 17.48±15.35 | |  |
| NK^56dim57-^/Lym | 1 organ | 3 | 7.45±6.38 | significant (**) | NK^56dim57-^/NK^56+^ | 1 organ | 4 | 48.88±15.45 | |  |
|  | more than 1 organ | 8 | 2.07±2.35 |  |  | more than 1 organ | 8 | 53.81±14.73 | |  |
| NK^56dim57+^/Lym | 1 organ | 3 | 2.97±1.95 |  | NK^56dim57+^/NK^56+^ | 1 organ | 4 | 26.68±14.79 | |  |
|  | more than 1 organ | 10 | 3.38±5.30 |  |  | more than 1 organ | 9 | 25.23±18.88 | |  |
| NK^56dim57-16bri^/Lym | 1 organ | 3 | 5.86±6.18 |  | NK^56dim57-16bri^/NK^56+^ | 1 organ | 3 | 33.57±17.71 | |  |
|  | more than 1 organ | 10 | 5.32±10.25 |  |  | more than 1 organ | 10 | 21.91±12.08 | |  |
| NK^56dim57+16+^/Lym | 1 organ | 3 | 2.95±1.94 |  | NK^56dim57+16+^/NK^56+^ | 1 organ | 4 | 26.58±14.79 | |  |
|  | more than 1 organ | 10 | 3.37±5.29 |  |  | more than 1 organ | 10 | 29.73±22.97 | |  |
| CTLA-4^+^NK^56+^/Lym | 1 organ | 4 | 0.58±0.32 | significant (**) | CTLA-4^+^NK^56+^/NK^56+^ | 1 organ | 4 | 27.68±16.84 | |  |
|  | more than 1 organ | 11 | 1.28±1.37 |  |  | more than 1 organ | 9 | 37.78±15.14 | |  |
| PD-1^+^NK^56+^/Lym | 1 organ | 4 | 0.03±0.02 | borderline significant (*) | PD-1^+^NK^56+^/NK^56+^ | 1 organ | 3 | 3.69±4.96 | |  |
|  | more than 1 organ | 12 | 0.07±0.08 |  |  | more than 1 organ | 12 | 14.07±12.12 | |  |
| PD-L1^+^NK^56+^/Lym | 1 organ | 4 | 0.12±0.16 | significant (**) | PD-L1^+^NK^56+^/NK^56+^ | 1 organ | 4 | 16.76±16.73 | |  |
|  | more than 1 organ | 11 | 0.05±0.04 |  |  | more than 1 organ | 10 | 16.58±14.89 | |  |
| PD-L2^+^NK^56+^/Lym | 1 organ | 4 | 0.01±0.01 | significant (**) | PD-L2^+^NK^56+^/NK^56+^ | 1 organ | 4 | 0.57±0.44 | | borderline significant (*) |
|  | more than 1 organ | 11 | 0.07±0.07 |  |  | more than 1 organ | 11 | 1.53±1.11 | |  |
| CD40L^+^NK^56+^/Lym | 1 organ | 4 | 0.13±0.09 |  | CD40L^+^NK^56+^/NK^56+^ | 1 organ | 3 | 5.76±2.40 | | borderline significant (*) |
|  | more than 1 organ | 12 | 0.10±0.14 |  |  | more than 1 organ | 12 | 19.34±16.14 | |  |
| LAG3^+^NK^56+^/Lym | 1 organ | 4 | 0.05±0.04 |  | LAG3^+^NK^56+^/NK^56+^ | 1 organ | 4 | 86.25±13.87 | |  |
|  | more than 1 organ | 11 | 0.03±0.04 |  |  | more than 1 organ | 9 | 88.92±9.24 | |  |
| 2B4^+^NK^56+^/Lym | 1 organ | 4 | 3.71±3.01 |  | 2B4^+^NK^56+^/NK^56+^ | 1 organ | 3 | 54.27±12.17 | |  |
|  | more than 1 organ | 9 | 2.90±3.00 |  |  | more than 1 organ | 10 | 66.70±19.42 | |  |
| TIM3^+^NK^56+^/Lym | 1 organ | 4 | 1.61±0.90 |  |  |  |  |  | |  |
|  | more than 1 organ | 9 | 1.76±1.48 |  |  |  |  |  | |  |
| KLRG1^+^NK^56+^/Lym | 1 organ | 4 | 0.62±0.59 | significant (**) | KLRG1^+^NK^56+^/NK^56+^ | 1 organ | 4 | 27.00±19.07 | |  |
|  | more than 1 organ | 12 | 1.23±1.17 |  |  | more than 1 organ | 12 | 21.26±13.81 | |  |
| TIGIT^+^NK^56+^/Lym | 1 organ | 4 | 0.94±0.78 | significant (**) | TIGIT^+^NK^56+^/NK^56+^ | 1 organ | 4 | 29.70±13.35 | |  |
|  | more than 1 organ | 11 | 1.98±1.82 |  |  | more than 1 organ | 9 | 30.06±15.72 | |  |
| CIK^56+^/Lym | 1 organ | 4 | 0.39±0.52 |  |  |  |  |  | |  |
|  | more than 1 organ | 12 | 0.37±0.36 |  |  |  |  |  | |  |
| CD40L^+^CIK^56+^/Lym | 1 organ | 4 | 0.09±0.11 | significant (**) | CD40L^+^CIK^56+^/CIK^56+^ | 1 organ | 4 | 5.08±4.72 | |  |
|  | more than 1 organ | 12 | 0.28±0.31 |  |  | more than 1 organ | 11 | 12.63±13.47 | |  |
| MAIT cells /Lym | 1 organ | 4 | 0.00±0.00 | borderline significant (*) |  |  |  |  | |  |
|  | more than 1 organ | 11 | 0.01±0.01 |  |  |  |  |  | |  |
| CTLA-4^+^CIK^56+^/Lym | 1 organ | 4 | 0.04±0.06 |  | CTLA-4^+^CIK^56+^/CIK^56+^ | 1 organ | 4 | 14.27±11.33 | |  |
|  | more than 1 organ | 12 | 0.04±0.04 |  |  | more than 1 organ | 11 | 17.59±10.63 | |  |
| PD-1^+^CIK^56+^/Lym | 1 organ | 4 | 0.13±0.26 |  | PD-1^+^CIK^56+^/CIK^56+^ | 1 organ | 4 | 3.89±2.87 | |  |
|  | more than 1 organ | 12 | 0.11±0.17 |  |  | more than 1 organ | 12 | 5.72±4.81 | |  |
| PD-L1^+^CIK^56+^/Lym | 1 organ | 4 | 0.13±0.25 |  | PD-L1^+^CIK^56+^/CIK^56+^ | 1 organ | 4 | 11.28±10.28 | |  |
|  | more than 1 organ | 12 | 0.32±0.35 |  |  | more than 1 organ | 9 | 10.57±9.88 | |  |
| PD-L2^+^CIK^56+^/Lym | 1 organ | 4 | 0.01±0.01 |  | PD-L2^+^CIK^56+^/CIK^56+^ | 1 organ | 4 | 5.96±9.41 | |  |
|  | more than 1 organ | 12 | 0.08±0.17 |  |  | more than 1 organ | 11 | 11.49±11.11 | |  |
| KLRG1^+^CIK^56+^/Lym | 1 organ | 4 | 0.52±0.74 | highly significant (***) | KLRG1^+^CIK^56+^/CIK^56+^ | 1 organ | 4 | 54.40±20.01 | |  |
|  | more than 1 organ | 12 | 0.07±0.07 |  |  | more than 1 organ | 9 | 58.08±29.87 | |  |
| TIGIT^+^CIK^56+^/Lym | 1 organ | 4 | 0.44±0.54 | highly significant (***) | TIGIT^+^CIK^56+^/CIK^56+^ | 1 organ | 3 | 34.43±11.25 | |  |
|  | more than 1 organ | 12 | 0.06±0.06 |  |  | more than 1 organ | 9 | 33.90±15.81 | |  |
| iNKT/Lym | 1 organ | 4 | 0.14±0.10 |  |  |  |  |  | |  |
|  | more than 1 organ | 10 | 0.28±0.44 |  |  |  |  |  | |  |
| iNKT^3+^/Lym | 1 organ | 4 | 0.00±0.01 |  |  |  |  |  | |  |
|  | more than 1 organ | 10 | 0.12±0.34 |  |  |  |  |  | |  |
| iNKT^161+^/Lym | 1 organ | 4 | 0.07±0.05 |  |  |  |  |  | |  |
|  | more than 1 organ | 10 | 0.14±0.18 |  |  |  |  |  | |  |

# **Supplementary Table 5 — DLI-induced GvHD**

| Myeloid cells | | | | | | | | | |
| --- | --- | --- | --- | --- | --- | --- | --- | --- | --- |
| In non Lym-gate | | | | | In subsets | | | | |
| DLI induced GvHD | Yes/no | N | % Mean  SD | *p* | DLI induced GvHD | Yes/no | N | % Mean  SD | *p* |
| CD14^+^/non Lym | yes, DLI | 6 | 8.16±6.65 |  |  |  |  |  |  |
|  | no DLI | 10 | 5.85±5.60 |  |  |  |  |  |  |
| CTLA-4^+^Mo/non Lym | yes, DLI | 7 | 2.01±2.14 |  | CTLA-4^+^Mo/Mo | yes, DLI | 6 | 14.71±4.30 |  |
|  | no DLI | 10 | 1.31±1.80 |  |  | no DLI | 10 | 20.23±5.85 |  |
| PD-1^+^Mo/non Lym | yes, DLI | 7 | 1.33±1.63 |  | PD-1^+^Mo/Mo | yes, DLI | 7 | 15.68±14.37 |  |
|  | no DLI | 10 | 1.22±1.36 |  |  | no DLI | 10 | 13.60±7.81 |  |
| PD-L1^+^Mo/non Lym | yes, DLI | 7 | 4.90±3.56 | borderline significant (*) | PD-L1^+^Mo/Mo | yes, DLI | 5 | 82.50±14.45 |  |
|  | no DLI | 9 | 4.26±1.96 |  |  | no DLI | 10 | 91.25±8.27 |  |
| LAG3^+^Mo/non Lym | yes, DLI | 7 | 0.11±0.04 |  | LAG3^+^Mo/Mo | yes, DLI | 7 | 1.47±0.66 | significant (**) |
|  | no DLI | 11 | 0.09±0.05 |  |  | no DLI | 11 | 1.63±1.22 |  |
| 2B4^+^Mo/non Lym | yes, DLI | 6 | 6.46±2.31 |  | 2B4^+^Mo/Mo | yes, DLI | 7 | 92.57±10.30 | highly significant (***) |
|  | no DLI | 8 | 4.84±2.77 |  |  | no DLI | 10 | 99.45±0.64 |  |
| Tim3^+^Mo/non Lym | yes, DLI | 6 | 5.94±2.20 |  | Tim3^+^Mo/Mo | yes, DLI | 7 | 87.27±7.43 |  |
|  | no DLI | 8 | 3.54±2.21 |  |  | no DLI | 9 | 92.38±5.61 |  |
| DC^80+^/non Lym | yes, DLI | 7 | 1.14±0.50 | borderline significant (*) |  |  |  |  |  |
|  | no DLI | 10 | 1.35±0.81 |  |  |  |  |  |  |
| DC_mat_^80+^/non Lym | yes, DLI | 7 | 0.25±0.17 |  | DC_mat_^80+^/DC^80+^ | yes, DLI | 7 | 24.15±14.29 |  |
|  | no DLI | 11 | 0.46±0.47 |  |  | no DLI | 10 | 24.59±10.14 |  |
| DC_tol_^80+^/non Lym | yes, DLI | 7 | 0.51±0.34 |  | DC_tol_^80+^/DC^80+^ | yes, DLI | 7 | 33.99±14.60 |  |
|  | no DLI | 11 | 0.40±0.36 |  |  | no DLI | 7 | 22,91±16,82 |  |
| CTLA-4^+^DC^80+^/non Lym | yes, DLI | 7 | 0.24±0.12 | borderline significant (*) | CTLA-4^+^DC^80+^/DC^80+^ | yes, DLI | 6 | 23.03±12.53 |  |
|  | no DLI | 11 | 0.42±0.32 |  |  | no DLI | 9 | 24.11±16.29 |  |
| CTLA-4^+^DC_mat_^80+^/non Lym | yes, DLI | 7 | 0.16±0.09 |  | CTLA-4^+^DC_mat_^80+^/DC_mat_^80+^ | yes, DLI | 7 | 64.44±16.28 |  |
|  | no DLI | 11 | 0.25±0.18 |  |  | no DLI | 10 | 76.26±18.65 |  |
| PMN-MDSC/non Lym | yes, DLI | 6 | 13.80±17.22 |  | PMN-MDSC/CD11b+CD33^+^ | yes, DLI | 6 | 65.50±22.08 |  |
|  | no DLI | 11 | 19.68±12.60 |  |  | no DLI | 11 | 71.63±15.91 |  |
| M-MDSC/non Lym | yes, DLI | 6 | 8.54±6.77 |  | M-MDSC/CD11b+CD33^+^ | yes, DLI | 5 | 26.74±12.55 |  |
|  | no DLI | 10 | 5.91±6.07 |  |  | no DLI | 10 | 25.16±12.44 |  |
| CTLA-4^+^PMN-MDSC/non Lym | yes, DLI | 6 | 1.08±0.40 |  | CTLA-4^+^PMN-MDSC/PMN-MDSC | yes, DLI | 7 | 12.47±7.80 |  |
|  | no DLI | 11 | 1.68±2.16 |  |  | no DLI | 10 | 11.75±13.25 |  |
| CTLA-4^+^M-MDSC/non Lym | yes, DLI | 7 | 2.46±2.52 |  | CTLA-4^+^M-MDSC/PMN-MDSC | yes, DLI | 6 | 18.27±5.45 |  |
|  | no DLI | 11 | 2.76±4.12 |  |  | no DLI | 9 | 26.87±8.31 |  |
| In non Lym-Gate | | | | | In subsets | | | | |
| DLI induced GvHD | Yes/no | N | % Mean  SD | *p* | DLI induced GvHD | Yes/no | N | % Mean  SD | *p* |
| Adaptive cells | | | | | | | | | |
| T/Lym | yes, DLI | 6 | 0.05±0.03 |  |  |  |  |  |  |
|  | no DLI | 10 | 0.06±0.09 |  |  |  |  |  |  |
| T^4+^/Lym | yes, DLI | 6 | 1.15±0.81 |  | T^4+^/T^3+^ | yes, DLI | 6 | 25.00±10.89 |  |
|  | no DLI | 10 | 1.52±1.79 |  |  | no DLI | 7 | 32.00±16.42 |  |
| T^4-^/Lym | yes, DLI | 6 | 3.84±2.32 | highly significant (***) | T^4-^/T^3+^ | yes, DLI | 6 | 75.00±10.89 |  |
|  | no DLI | 8 | 0.93±0.64 |  |  | no DLI | 8 | 65.25±17.20 |  |
| T_prol-early_ /Lym | yes, DLI | 6 | 0.45±0.32 |  | T_prol-early_ /T^3+^ | yes, DLI | 5 | 10.65±6.24 |  |
|  | no DLI | 10 | 0.32±0.30 |  |  | no DLI | 10 | 5.96±4.58 |  |
| T_prol-late_ /Lym | yes, DLI | 6 | 0.53±0.32 |  | T_prol-late_ /T^3+^ | yes, DLI | 5 | 12.14±8.15 |  |
|  | no DLI | 10 | 0.54±0.48 |  |  | no DLI | 9 | 9.37±9.28 |  |
| T_nn_ /Lym | yes, DLI | 6 | 2.22±0.99 |  | T_nn_ /T^3+^ | yes, DLI | 6 | 48.63±11.27 |  |
|  | no DLI | 9 | 1.45±1.71 |  |  | no DLI | 9 | 58.74±17.82 |  |
| T_em_ /Lym | yes, DLI | 6 | 2.13±0.99 |  | T_em_ /T^3+^ | yes, DLI | 5 | 49.32±6.71 |  |
|  | no DLI | 9 | 1.41±1.68 |  |  | no DLI | 9 | 57.34±17.36 |  |
| T_cm_ /Lym | yes, DLI | 6 | 0.09±0.09 | significant (**) | T_cm_ /T^3+^ | yes, DLI | 5 | 1.66±1.82 |  |
|  | no DLI | 10 | 0.04±0.03 |  |  | no DLI | 10 | 1.34±1.13 |  |
| CD28^+^T/Lym | yes, DLI | 5 | 2.31±0.81 |  | CD28^+^T/T^3+^ | yes, DLI | 4 | 64.23±13.19 |  |
|  | no DLI | 9 | 1.56±1.65 |  |  | no DLI | 9 | 60.38±25.17 |  |
| CD28^+^T^4+^/Lym | yes, DLI | 5 | 1.22±0.75 |  | CD28^+^T^4+^/T^4+^ | yes, DLI | 6 | 91.60±10.69 |  |
|  | no DLI | 10 | 1.49±2.26 |  |  | no DLI | 9 | 88.41±11.61 |  |
| CD28^+^T^4-^/Lym | yes, DLI | 5 | 1.08±0.25 |  | CD28^+^T^4-^/T^4-^ | yes, DLI | 5 | 43.80±19.15 |  |
|  | no DLI | 9 | 0.90±1.41 |  |  | no DLI | 10 | 42.12±27.16 |  |
| CD40L^+^T^4+^/Lym | yes, DLI | 5 | 0.16±0.19 |  | CD40L^+^T/T^3+^ | yes, DLI | 2 | 7.24±7.87 |  |
|  | no DLI | 10 | 0.19±0.20 |  |  | no DLI | 5 | 4.30±3.94 |  |
| CD40L^+^T^4-^/Lym | yes, DLI | 5 | 0.05±0.06 |  | CD40L^+^T^4+^/T^4+^ | yes, DLI | 4 | 12.23±9.96 |  |
|  | no DLI | 10 | 0.08±0.12 |  |  | no DLI | 9 | 5.48±6.12 |  |
|  |  |  |  |  | CD40L^+^T^4-^/T^4-^ | yes, DLI | 5 | 1.96±1.94 |  |
|  |  |  |  |  |  | no DLI | 9 | 1.30±1.49 |  |
| T^H1/Gui^/Lym | yes, DLI | 6 | 0.81±1.50 |  | T^H1/Gui^/T^4+^ | yes, DLI | 6 | 11.90±7.87 |  |
|  | no DLI | 11 | 0.56±0.92 |  |  | no DLI | 10 | 13.87±8.69 |  |
| T^H2/Gui^/Lym | yes, DLI | 7 | 0.25±0.19 |  | T^H2/Gui^/T^4+^ | yes, DLI | 7 | 7.70±2.36 |  |
|  | no DLI | 11 | 0.43±0.62 |  |  | no DLI | 10 | 7.58±4.02 |  |
| T^H17/Gui^/Lym | yes, DLI | 7 | 0.20±0.27 | significant (**) | T^H17/Gui^/T^4+^ | yes, DLI | 7 | 5.56±3.19 |  |
|  | no DLI | 11 | 0.67±1.01 |  |  | no DLI | 8 | 10.26±7.43 |  |
| γδ^+^T/Lym | yes, DLI | 6 | 6.53±4.85 |  | γδ^+^T/T^3+^ | yes, DLI | 6 | 6.53±4.85 |  |
|  | no DLI | 8 | 4.75±3.81 |  |  | no DLI | 8 | 4.75±3.81 |  |
| γδ^+^T^4+^/Lym | yes, DLI | 6 | 0.04±0.04 | significant (**) | γδ^+^T^4+^/T^3+^ | yes, DLI | 6 | 0.87±0.95 |  |
|  | no DLI | 9 | 0.02±0.02 |  |  | no DLI | 9 | 0.72±0.76 |  |
| γδ^+^T^4-^/Lym | yes, DLI | 6 | 0.53±0.79 |  | γδ^+^T^4-^/T^3+^ | yes, DLI | 6 | 5.66±4.41 |  |
|  | no DLI | 9 | 0.23±0.42 |  |  | no DLI | 8 | 4.05±3.85 |  |
| Intß7^+^T/Lym | yes, DLI | 6 | 1.83±1.27 |  | Intß7^+^T/T^3+^ | yes, DLI | 5 | 34.05±17.16 |  |
|  | no DLI | 9 | 1.22±1.29 |  |  | no DLI | 9 | 28.16±14.91 |  |
| Intß7^+^T^4+^/Lym | yes, DLI | 6 | 0.56±0.82 |  | Intß7^+^T^4+^/T^3+^ | yes, DLI | 5 | 11.72±6.56 |  |
|  | no DLI | 9 | 0.25±0.42 |  |  | no DLI | 8 | 12.27±7.67 |  |
| Intß7^+^T^4-^/Lym | yes, DLI | 6 | 1.37±0.86 |  | Intß7^+^T^4-^/T^3+^ | yes, DLI | 4 | 30.95±4.25 | borderline significant (*) |
|  | no DLI | 9 | 0.81±1.09 |  |  | no DLI | 8 | 18.41±9.84 |  |
| CD127^+^T/Lym | yes, DLI | 7 | 3.11±2.50 |  | CD127^+^T/T^3+^ | yes, DLI | 4 | 54.20±28.02 | significant (*) |
|  | no DLI | 9 | 1.77±1.69 |  |  | no DLI | 9 | 58.43±16.93 |  |
|  |  |  |  |  | T^4+^_reg_ /T^4+^ | yes, DLI | 5 | 10.77±3.92 |  |
|  |  |  |  |  |  | no DLI | 8 | 6.15±3.64 |  |
| T^4+^_reg_/Lym | yes, DLI | 6 | 0.18±0.08 |  | T^4+^_reg_ /T^3+^ | yes, DLI | 6 | 3.14±1.38 | significant (*) |
|  | no DLI | 9 | 0.12±0.13 |  |  | no DLI | 9 | 3.51±3.25 |  |
|  |  |  |  |  | T^4-^_reg_ /T^4-^ | yes, DLI | 6 | 0.66±0.74 |  |
|  |  |  |  |  |  | no DLI | 9 | 1.18±1.77 |  |
| T^4-^_reg_/Lym | yes, DLI | 6 | 0.08±0.17 | significant (**) | T^4-^_reg_ /T^3+^ | yes, DLI | 6 | 0.53±0.65 |  |
|  | no DLI | 9 | 0.01±0.01 |  |  | no DLI | 9 | 0.51±0.73 |  |
| CTLA-4^+^T/Lym | yes, DLI | 0^a^ |  |  | CTLA-4^+^T/T^3+^ | yes, DLI | 0^a^ |  |  |
|  | no DLI | 1 | 0.20 |  |  | no DLI | 1 | 0.92 |  |
| CTLA-4^+^T^4+^/Lym | yes, DLI | 6 | 0.01±0.01 |  | CTLA-4^+^T^4+^/T^4+^ | yes, DLI | 6 | 0.59±0.20 | significant (*) |
|  | no DLI | 11 | 0.02±0.02 |  |  | no DLI | 10 | 0.74±0.77 |  |
| CTLA-4^+^T^4-^/Lym | yes, DLI | 6 | 0.05±0.04 |  | CTLA-4^+^T^4-^/T^4-^ | yes, DLI | 6 | 0.88±0.86 |  |
|  | no DLI | 11 | 0.04±0.05 |  |  | no DLI | 11 | 1.23±1.07 |  |
| CD28^+^CTLA-4^+^T/Lym | yes, DLI | 0^a^ |  |  | CD28^+^CTLA-4^+^T^4-^/T^4-^ | yes, DLI | 6 | 0.55±0.32 |  |
|  | no DLI | 1 | 0.11 |  |  | no DLI | 9 | 0.51±0.49 |  |
| CD28^+^CTLA-4^+^T^4+^/Lym | yes, DLI | 6 | 0.07±0.14 | significant (**) | CD28^+^CTLA-4^+^T^4+^/T^4+^ | yes, DLI | 6 | 0.37±0.26 |  |
|  | no DLI | 11 | 0.01±0.02 |  |  | no DLI | 9 | 0.43±0.36 |  |
| CD28^+^CTLA-4^+^T^4-^/Lym | yes, DLI | 6 | 0.02±0.01 |  | CD28^+^CTLA-4^+^T^4-^/T^4-^ |  |  |  |  |
|  | no DLI | 11 | 0.02±0.04 |  |  |  |  |  |  |
| PD-1^+^T/Lym | yes, DLI | 6 | 0.57±0.59 |  | PD-1^+^T/T^3+^ | yes, DLI | 5 | 24.77±12.82 |  |
|  | no DLI | 11 | 1.59±2.29 |  |  | no DLI | 10 | 16.30±8.97 |  |
| PD-1^+^T^4+^/Lym | yes, DLI | 6 | 0.11±0.11 |  | PD-1^+^T^4+^/T^4+^ | yes, DLI | 5 | 15.78±9.73 | significant (*) |
|  | no DLI | 11 | 0.16±0.20 |  |  | no DLI | 9 | 11.08±4.90 |  |
| PD-1^+^T^4-^/Lym | yes, DLI | 6 | 0.46±0.50 | borderline significant (*) | PD-1^+^T^4-^/T^4-^ | yes, DLI | 5 | 84.22±9.73 |  |
|  | no DLI | 11 | 1.44±2.09 |  |  | no DLI | 10 | 86.34±9.35 |  |
| PD-L1^+^T/Lym | yes, DLI | 6 | 0.80±1.38 |  | PD-L1^+^T/T^3+^ | yes, DLI | 6 | 12.59±5.72 |  |
|  | no DLI | 11 | 0.62±0.60 |  |  | no DLI | 9 | 10.77±5.25 |  |
| PD-L1^+^T^4+^/Lym | yes, DLI | 6 | 0.38±0.68 |  | PD-L1^+^T^4+^/T^4+^ | yes, DLI | 5 | 54.98±17.69 |  |
|  | no DLI | 11 | 0.43±0.47 |  |  | no DLI | 9 | 66.00±20.06 |  |
| PD-L1^+^T^4-^/Lym | yes, DLI | 6 | 0.42±0.70 | significant (**) | PD-L1^+^T^4-^/T^4-^ | yes, DLI | 5 | 45.02±17.69 |  |
|  | no DLI | 11 | 0.19±0.20 |  |  | no DLI | 8 | 31.87±20.34 |  |
| LAG3^+^T/Lym | yes, DLI | 0^a^ |  |  | LAG3^+^T/T^3+^ | yes, DLI | 0^a^ |  |  |
|  | no DLI | 2 | 0.11±0.05 |  |  | no DLI | 0^a^ |  |  |
| LAG3^+^T^4+^/Lym | yes, DLI | 7 | 0.02±0.02 |  | LAG3^+^T^4+^/T^4+^ | yes, DLI | 7 | 0.81±0.48 |  |
|  | no DLI | 10 | 0.02±0.02 |  |  | no DLI | 8 | 0.85±0.52 |  |
| LAG3^+^T^4-^/Lym | yes, DLI | 7 | 0.06±0.07 |  | LAG3^+^T^4-^/T^4-^ | yes, DLI | 7 | 0.87±0.68 |  |
|  | no DLI | 10 | 0.05±0.09 |  |  | no DLI | 9 | 1.08±0.80 |  |
| 2B4^+^T/Lym | yes, DLI | 5 | 3.58±3.47 | significant (**) | 2B4^+^T/T^3+^ | yes, DLI | 5 | 35.32±17.81 |  |
|  | no DLI | 5 | 1.46±1.34 |  |  | no DLI | 5 | 37.88±11.10 |  |
| 2B4^+^T^4+^/Lym | yes, DLI | 6 | 0.25±0.47 |  | 2B4^+^T^4+^/T^4+^ | yes, DLI | 4 | 1.73±0.52 |  |
|  | no DLI | 11 | 0.06±0.11 |  |  | no DLI | 11 | 3.61±6.89 |  |
| 2B4^+^T^4-^/Lym | yes, DLI | 5 | 1.37±1.24 |  | 2B4^+^T^4-^/T^4-^ | yes, DLI | 6 | 38.80±24.04 |  |
|  | no DLI | 10 | 0.73±1.16 |  |  | no DLI | 6 | 47.62±10.52 |  |
| TIM3^+^T/Lym | yes, DLI | 5 | 0.33±0.20 |  | TIM3^+^T/T^3+^ | yes, DLI | 5 | 5.94±5.07 |  |
|  | no DLI | 7 | 0.34±0.19 |  |  | no DLI | 5 | 6.15±6.16 |  |
| TIM3^+^T^4+^/Lym | yes, DLI | 7 | 0.04±0.05 |  | TIM3^+^T^4+^/T^4+^ | yes, DLI | 5 | 2.29±0.74 |  |
|  | no DLI | 10 | 0.03±0.03 |  |  | no DLI | 10 | 1.27±0.85 |  |
| TIM3^+^T^4-^/Lym | yes, DLI | 7 | 0.20±0.17 |  | TIM3^+^T^4-^/T^4-^ | yes, DLI | 6 | 6.18±5.49 |  |
|  | no DLI | 10 | 0.23±0.17 |  |  | no DLI | 9 | 5.95±5.98 |  |
| KLRG1^+^T/Lym | yes, DLI | 6 | 1.04±1.79 | borderline significant (*) | KLRG1^+^T/T^3+^ | yes, DLI | 4 | 34.98±21.28 |  |
|  | no DLI | 10 | 2.02±2.70 |  |  | no DLI | 9 | 35.36±18.13 |  |
| KLRG1^+^T^4+^/Lym | yes, DLI | 6 | 0.10±0.14 | borderline significant (*) | KLRG1^+^T^4+^/T^4+^ | yes, DLI | 5 | 10.57±11.44 |  |
|  | no DLI | 11 | 0.34±0.53 |  |  | no DLI | 10 | 14.25±10.78 |  |
| KLRG1^+^T^4-^/Lym | yes, DLI | 6 | 0.94±1.67 | significant (**) | KLRG1^+^T^4-^/T^4-^ | yes, DLI | 5 | 89.42±11.43 |  |
|  | no DLI | 11 | 2.23±2.79 |  |  | no DLI | 11 | 82.58±14.70 |  |
| TIGIT^+^T/Lym | yes, DLI | 5 | 0.44±0.40 |  | TIGIT^+^T/T^3+^ | yes, DLI | 4 | 37.70±22.07 |  |
|  | no DLI | 10 | 2.21±2.05 |  |  | no DLI | 11 | 33.22±9.32 |  |
| TIGIT^+^T^4+^/Lym | yes, DLI | 6 | 0.37±0.65 |  | TIGIT^+^T^4+^/T^4+^ | yes, DLI | 6 | 66.55±27.89 |  |
|  | no DLI | 11 | 0.77±0.83 |  |  | no DLI | 9 | 66.81±25.57 |  |
| TIGIT^+^T^4-^/Lym | yes, DLI | 6 | 1.32±2.44 |  | TIGIT^+^T^4-^/T^4-^ | yes, DLI | 6 | 33.46±27.88 |  |
|  | no DLI | 11 | 2.09±2.48 |  |  | no DLI | 9 | 33.47±26.31 |  |
| B/Lym | yes, DLI | 6 | 0.05±0.05 |  |  |  |  |  |  |
|  | no DLI | 9 | 0.04±0.07 |  |  |  |  |  |  |
| B_nai_/Lym | yes, DLI | 6 | 4.65±4.80 |  | B_nai_/B | yes, DLI | 6 | 65.35±26.51 |  |
|  | no DLI | 8 | 3.31±6.96 |  |  | no DLI | 6 | 56.72±24.58 |  |
| B_mem(class)_/Lym | yes, DLI | 6 | 0.33±0.23 |  | B_mem(class)/_B | yes, DLI | 6 | 11.56±13.87 |  |
|  | no DLI | 8 | 0.50±0.65 |  |  | no DLI | 8 | 19.14±9.86 |  |
| B_mem_/Lym | yes, DLI | 6 | 0.15±0.14 |  | B_mem_/B | yes, DLI | 6 | 4.07±3.14 |  |
|  | no DLI | 9 | 0.19±0.35 |  |  | no DLI | 9 | 4.70±5.41 |  |
| B_reg_/Lym | yes, DLI | 0^a^ |  |  | B_reg_/B | yes, DLI | 6 | 2.00±1.97 |  |
|  | no DLI | 0^a^ |  |  |  | no DLI | 9 | 1.66±2.22 |  |
| Innate cells | | | | | | | | | |
| In Lym-Gate | | | | | In subsets | | | | |
| DLI induced GvHD | Yes/no | N | % Mean  SD | *p* | DLI induced GvHD | Yes/no | N | % Mean  SD | *p* |
| NK^56+^/Lym | yes, DLI | 6 | 0.06±0.08 |  |  |  |  |  |  |
|  | no DLI | 10 | 0.06±0.05 |  |  |  |  |  |  |
| NK^56bri^/Lym | yes, DLI | 6 | 2.08±3.28 |  | NK^56bri^/NK^56+^ | yes, DLI | 6 | 8.65±4.87 | significant (**) |
|  | no DLI | 8 | 1.46±2.63 |  |  | no DLI | 8 | 19.53±15.72 |  |
| NK^56bri16-^/Lym | yes, DLI | 6 | 1.09±1.90 |  | NK^56bri16-^/NK^56+^ | yes, DLI | 6 | 3.66±2.69 | borderline significant (*) |
|  | no DLI | 9 | 1.94±3.49 |  |  | no DLI | 9 | 10.09±11.93 |  |
| NK^56bri16+^/Lym | yes, DLI | 6 | 0.99±1.40 |  | NK^56bri16+^/NK^56+^ | yes, DLI | 6 | 4.99±2.64 | borderline significant (*) |
|  | no DLI | 9 | 2.41±5.84 |  |  | no DLI | 8 | 8.86±6.48 |  |
| NK^56dim16+^/Lym | yes, DLI | 6 | 18.49±34.49 | borderline significant (*) | NK^56dim16+^/NK^56+^ | yes, DLI | 5 | 77.72±10.72 | significant (**) |
|  | no DLI | 9 | 6.86±10.81 |  |  | no DLI | 9 | 63.71±23.35 |  |
| NK^56bri57-^/Lym | yes, DLI | 6 | 2.13±3.34 |  | NK^56bri57-^/NK^56+^ | yes, DLI | 6 | 8.48±4.81 | significant (**) |
|  | no DLI | 8 | 1.46±2.55 |  |  | no DLI | 8 | 20.09±15.91 |  |
| NK^56dim57-^/Lym | yes, DLI | 5 | 4.49±5.96 |  | NK^56dim57-^/NK^56+^ | yes, DLI | 6 | 54.68±17.95 |  |
|  | no DLI | 8 | 2.22±2.38 |  |  | no DLI | 8 | 44.31±13.94 |  |
| NK^56dim57+^/Lym | yes, DLI | 5 | 1.56±1.68 |  | NK^56dim57+^/NK^56+^ | yes, DLI | 6 | 36.49±19.25 | significant (**) |
|  | no DLI | 10 | 3.90±5.16 |  |  | no DLI | 7 | 16.40±7.71 |  |
| NK^56dim57-16bri^/Lym | yes, DLI | 5 | 2.97±5.61 |  | NK^56dim57-16bri^/NK^56+^ | yes, DLI | 5 | 23.24±15.29 |  |
|  | no DLI | 10 | 5.70±10.08 |  |  | no DLI | 10 | 24.61±12.28 |  |
| NK^56dim57+16+^/Lym | yes, DLI | 5 | 1.56±1.67 |  | NK^56dim57+16+^/NK^56+^ | yes, DLI | 6 | 36.40±19.28 |  |
|  | no DLI | 10 | 3.88±5.16 |  |  | no DLI | 8 | 23.15±20.60 |  |
| CTLA-4^+^NK^56+^/Lym | yes, DLI | 7 | 1.48±1.48 |  | CTLA-4^+^NK^56+^/NK^56+^ | yes, DLI | 5 | 29.56±11.62 |  |
|  | no DLI | 10 | 0.84±0.93 |  |  | no DLI | 10 | 35.24±16.65 |  |
| PD-1^+^NK^56+^/Lym | yes, DLI | 7 | 0.05±0.05 |  | PD-1^+^NK^56+^/NK^56+^ | yes, DLI | 7 | 8.22±7.78 |  |
|  | no DLI | 11 | 0.06±0.08 |  |  | no DLI | 10 | 17.03±12.90 |  |
| PD-L1^+^NK^56+^/Lym | yes, DLI | 7 | 0.06±0.04 |  | PD-L1^+^NK^56+^/NK^56+^ | yes, DLI | 6 | 13.51±12.81 |  |
|  | no DLI | 10 | 0.06±0.10 |  |  | no DLI | 10 | 18.88±14.68 |  |
| PD-L2^+^NK^56+^/Lym | yes, DLI | 7 | 0.08±0.09 | significant (**) | PD-L2^+^NK^56+^/NK^56+^ | yes, DLI | 6 | 1.15±0.98 |  |
|  | no DLI | 10 | 0.03±0.03 |  |  | no DLI | 11 | 1.23±1.07 |  |
| CD40L^+^NK^56+^/Lym | yes, DLI | 7 | 0.14±0.17 | borderline significant (*) | CD40L^+^NK^56+^/NK^56+^ | yes, DLI | 6 | 21.61±17.55 |  |
|  | no DLI | 11 | 0.06±0.08 |  |  | no DLI | 11 | 13.39±12.37 |  |
| LAG3^+^NK^56+^/Lym | yes, DLI | 7 | 0.03±0.03 |  | LAG3^+^NK^56+^/NK^56+^ | yes, DLI | 6 | 89.87±7.66 |  |
|  | no DLI | 10 | 0.04±0.04 |  |  | no DLI | 9 | 88.46±11.66 |  |
| 2B4^+^NK^56+^/Lym | yes, DLI | 7 | 2.84±1.15 | significant (**) | 2B4^+^NK^56+^/NK^56+^ | yes, DLI | 6 | 59.15±17.38 |  |
|  | no DLI | 8 | 3.26±3.79 |  |  | no DLI | 8 | 70.33±19.42 |  |
| TIM3^+^NK^56+^/Lym | yes, DLI | 7 | 1.65±0.53 | borderline significant (*) |  |  |  |  |  |
|  | no DLI | 8 | 1.54±1.69 |  |  |  |  |  |  |
| KLRG1^+^NK^56+^/Lym | yes, DLI | 7 | 1.34±1.17 |  | KLRG1^+^NK^56+^/NK^56+^ | yes, DLI | 7 | 21.66±13.54 |  |
|  | no DLI | 11 | 1.02±1.10 |  |  | no DLI | 10 | 24.44±15.83 |  |
| TIGIT^+^NK^56+^/Lym | yes, DLI | 7 | 2.44±1.77 |  | TIGIT^+^NK^56+^/NK^56+^ | yes, DLI | 6 | 30.33±9.03 |  |
|  | no DLI | 10 | 1.27±1.45 |  |  | no DLI | 9 | 31.10±16.84 |  |
| CIK^56+^/Lym | yes, DLI | 7 | 0.23±0.22 | significant (**) |  |  |  |  |  |
|  | no DLI | 11 | 0.43±0.43 |  |  |  |  |  |  |
| CD40L^+^CIK^56+^/Lym | yes, DLI | 7 | 0.36±0.36 | highly significant (***) | CD40L^+^CIK^56+^/CIK^56+^ | yes, DLI | 6 | 8.99±7.80 |  |
|  | no DLI | 11 | 0.15±0.16 |  |  | no DLI | 11 | 10.45±13.41 |  |
| MAIT cells /Lym | yes, DLI | 6 | 0.00±0.00 |  |  |  |  |  |  |
|  | no DLI | 11 | 0.01±0.01 |  |  |  |  |  |  |
| CTLA-4^+^CIK^56+^/Lym | yes, DLI | 7 | 0.03±0.03 |  | CTLA-4^+^CIK^56+^/CIK^56+^ | yes, DLI | 7 | 22.36±13.05 | significant (**) |
|  | no DLI | 11 | 0.04±0.05 |  |  | no DLI | 9 | 11.15±4.02 |  |
| PD-1^+^CIK^56+^/Lym | yes, DLI | 7 | 0.18±0.27 |  | PD-1^+^CIK^56+^/CIK^56+^ | yes, DLI | 7 | 6.80±4.86 |  |
|  | no DLI | 11 | 0.15±0.25 |  |  | no DLI | 10 | 4.08±3.63 |  |
| PD-L1^+^CIK^56+^/Lym | yes, DLI | 7 | 0.24±0.38 |  | PD-L1^+^CIK^56+^/CIK^56+^ | yes, DLI | 6 | 5.85±3.25 | significant (**) |
|  | no DLI | 11 | 0.28±0.27 |  |  | no DLI | 9 | 12.82±10.78 |  |
| PD-L2^+^CIK^56+^/Lym | yes, DLI | 7 | 0.02±0.01 |  | PD-L2^+^CIK^56+^/CIK^56+^ | yes, DLI | 6 | 14.84±12.62 |  |
|  | no DLI | 11 | 0.08±0.18 |  |  | no DLI | 11 | 6.75±7.49 |  |
| KLRG1^+^CIK^56+^/Lym | yes, DLI | 7 | 0.04±0.05 | borderline significant (*) | KLRG1^+^CIK^56+^/CIK^56+^ | yes, DLI | 5 | 50.46±22.39 |  |
|  | no DLI | 11 | 0.26±0.46 |  |  | no DLI | 10 | 64.35±26.83 |  |
| TIGIT^+^CIK^56+^/Lym | yes, DLI | 7 | 0.05±0.06 | borderline significant (*) | TIGIT^+^CIK^56+^/CIK^56+^ | yes, DLI | 5 | 27.95±15.17 |  |
|  | no DLI | 11 | 0.21±0.35 |  |  | no DLI | 9 | 36.94±13.91 |  |
| iNKT/Lym | yes, DLI | 6 | 0.10±0.06 |  |  |  |  |  |  |
|  | no DLI | 10 | 0.29±0.43 |  |  |  |  |  |  |
| iNKT^3+^/Lym | yes, DLI | 6 | 0.01±0.01 |  |  |  |  |  |  |
|  | no DLI | 10 | 0.12±0.34 |  |  |  |  |  |  |
| iNKT^161+^/Lym | yes, DLI | 6 | 0.05±0.03 |  |  |  |  |  |  |
|  | no DLI | 10 | 0.14±0.17 |  |  |  |  |  |  |

# **Supplementary Table 6 — Overlap a/cGvHD**

| Myeloid cells | | | | | | | | | |
| --- | --- | --- | --- | --- | --- | --- | --- | --- | --- |
| In non Lym-Gate | | | | | In subsets | | | | |
| Overlap cGvHD | Yes/no | N | % Mean  SD | *p* | Overlap cGvHD | Yes/no | N | % Mean  SD | *p* |
| CD14^+^/non Lym | yes, overlap | 5 | 7.17±8.48 | borderline significant (*) |  |  |  |  |  |
|  | no overlap | 11 | 6.50±4.83 |  |  |  |  |  |  |
| CTLA-4^+^Mo/non Lym | yes, overlap | 5 | 0.87±0.97 |  | CTLA-4^+^Mo/Mo | yes, overlap | 5 | 17.39±8.09 |  |
|  | no overlap | 12 | 1.90±2.16 |  |  | no overlap | 11 | 18.51±4.98 |  |
| PD-1^+^Mo/non Lym | yes, overlap | 4 | 0.67±0.22 | significant (**) | PD-1^+^Mo/Mo | yes, overlap | 4 | 16.70±9.08 |  |
|  | no overlap | 13 | 1.44±1.60 |  |  | no overlap | 13 | 13.77±11.30 |  |
| PD-L1^+^Mo/non Lym | yes, overlap | 5 | 3.46±1.52 |  | PD-L1^+^Mo/Mo | yes, overlap | 3 | 87.87±11.55 |  |
|  | no overlap | 11 | 5.03±3.01 |  |  | no overlap | 12 | 88.45±11.43 |  |
| LAG3^+^Mo/non Lym | yes, overlap | 5 | 0.09±0.05 |  | LAG3^+^Mo/Mo | yes, overlap | 5 | 1.72±1.32 |  |
|  | no overlap | 13 | 0.10±0.04 |  |  | no overlap | 13 | 1.51±0.93 |  |
| 2B4^+^Mo/non Lym | yes, overlap | 4 | 4.62±0.48 | significant (**) | 2B4^+^Mo/Mo | yes, overlap | 4 | 98.53±1.97 |  |
|  | no overlap | 10 | 5.90±3.05 |  |  | no overlap | 13 | 96.03±8.19 |  |
| Tim3^+^Mo/non Lym | yes, overlap | 4 | 4.26±0.64 | borderline significant (*) | Tim3^+^Mo/Mo | yes, overlap | 5 | 91.28±4.46 |  |
|  | no overlap | 10 | 4.69±2.91 |  |  | no overlap | 11 | 89.63±7.73 |  |
| DC^80+^/non Lym | yes, overlap | 5 | 0.99±0.49 |  |  |  |  |  |  |
|  | no overlap | 12 | 1.38±0.75 |  |  |  |  |  |  |
| DC_mat_^80+^/non Lym | yes, overlap | 5 | 0.29±0.18 |  | DC_mat_^80+^/DC^80+^ | yes, overlap | 4 | 26.75±15.93 |  |
|  | no overlap | 13 | 0.42±0.44 |  |  | no overlap | 13 | 23.69±10.65 |  |
| DC_tol_^80+^/non Lym | yes, overlap | 5 | 0.43±0.54 | borderline significant (*) | DC_tol_^80+^/DC^80+^ | yes, overlap | 3 | 18.20±13.57 |  |
|  | no overlap | 13 | 0.45±0.27 |  |  | no overlap | 11 | 31.24±16.24 |  |
| CTLA-4^+^DC^80+^/non Lym | yes, overlap | 5 | 0.32±0.10 |  | CTLA-4^+^DC^80+^/DC^80+^ | yes, overlap | 3 | 27.90±16.98 |  |
|  | no overlap | 13 | 0.36±0.32 |  |  | no overlap | 12 | 22.62±14.36 |  |
| CTLA-4^+^DC_mat_^80+^/non Lym | yes, overlap | 5 | 0.21±0.12 |  | CTLA-4^+^DC_mat_^80+^/DC_mat_^80+^ | yes, overlap | 5 | 74.60±11.85 |  |
|  | no overlap | 13 | 0.21±0.17 |  |  | no overlap | 12 | 70.06±20.59 |  |
| PMN-MDSC/non Lym | yes, overlap | 5 | 15.21±19.91 |  | PMN-MDSC/CD11b+CD33^+^ | yes, overlap | 5 | 63.10±25.19 |  |
|  | no overlap | 12 | 18.61±11.98 |  |  | no overlap | 12 | 72.12±14.42 |  |
| M-MDSC/non Lym | yes, overlap | 5 | 7.18±8.88 | borderline significant (*) | M-MDSC/CD11b+CD33^+^ | yes, overlap | 4 | 27.81±17.13 |  |
|  | no overlap | 11 | 6.77±5.21 |  |  | no overlap | 11 | 24.92±10.62 |  |
| CTLA-4^+^PMN-MDSC/non Lym | yes, overlap | 5 | 0.83±0.34 |  | CTLA-4^+^PMN-MDSC/PMN-MDSC | yes, overlap | 4 | 13.93±14.46 |  |
|  | no overlap | 12 | 1.73±2.04 |  |  | no overlap | 13 | 11.46±10.42 |  |
| CTLA-4^+^M-MDSC/non Lym | yes, overlap | 5 | 1.03±1.08 | significant (**) | CTLA-4^+^M-MDSC/PMN-MDSC | yes, overlap | 4 | 21.39±11.37 |  |
|  | no overlap | 13 | 3.27±3.93 |  |  | no overlap | 11 | 24.17±7.46 |  |
| In non Lym-Gate | | | | | In subsets | | | | |
| Overlap cGvHD | Yes/no | N | % Mean  SD | *p* | Overlap cGvHD | Yes/no | N | % Mean  SD | *p* |
| Adaptive cells | | | | | | | | | |
| T/Lym | yes, overlap | 5 | 0.02±0.02 |  |  |  |  |  |  |
|  | no overlap | 11 | 0.07±0.08 |  |  |  |  |  |  |
| T^4+^/Lym | yes, overlap | 5 | 1.00±0.96 |  | T^4+^/T^3+^ | yes, overlap | 5 | 38.66±13.82 |  |
|  | no overlap | 11 | 1.55±1.67 |  |  | no overlap | 8 | 22.59±10.76 |  |
| T^4-^/Lym | yes, overlap | 5 | 1.28±0.76 | highly significant (***) | T^4-^/T^3+^ | yes, overlap | 4 | 67.43±2.77 | significant (**) |
|  | no overlap | 9 | 2.67±2.51 |  |  | no overlap | 10 | 70.23±18.08 |  |
| T_prol-early_ /Lym | yes, overlap | 5 | 0.25±0.16 | borderline significant (*) | T_prol-early_ /T^3+^ | yes, overlap | 5 | 9.86±4.91 |  |
|  | no overlap | 11 | 0.42±0.34 |  |  | no overlap | 10 | 6.36±5.59 |  |
| T_prol-late_ /Lym | yes, overlap | 5 | 0.37±0.37 |  | T_prol-late_ /T^3+^ | yes, overlap | 5 | 11.90±6.19 | borderline significant (*) |
|  | no overlap | 11 | 0.61±0.43 |  |  | no overlap | 9 | 9.50±10.04 |  |
| T_nn_ /Lym | yes, overlap | 5 | 1.40±1.04 |  | T_nn_ /T^3+^ | yes, overlap | 5 | 58.64±8.99 |  |
|  | no overlap | 10 | 1.94±1.66 |  |  | no overlap | 10 | 52.73±18.57 |  |
| T_em_ /Lym | yes, overlap | 5 | 1.36±1.03 |  | T_em_ /T^3+^ | yes, overlap | 5 | 56.66±8.87 |  |
|  | no overlap | 10 | 1.87±1.64 |  |  | no overlap | 9 | 53.27±17.49 |  |
| T_cm_ /Lym | yes, overlap | 5 | 0.04±0.04 |  | T_cm_ /T^3+^ | yes, overlap | 5 | 1.69±1.49 |  |
|  | no overlap | 11 | 0.07±0.07 |  |  | no overlap | 10 | 1.32±1.33 |  |
| CD28^+^T/Lym | yes, overlap | 5 | 1.21±1.38 |  | CD28^+^T/T^3+^ | yes, overlap | 3 | 69.37±13.66 |  |
|  | no overlap | 9 | 2.17±1.39 |  |  | no overlap | 10 | 59.22±23.62 |  |
| CD28^+^T^4+^/Lym | yes, overlap | 5 | 0.62±0.78 |  | CD28^+^T^4+^/T^4+^ | yes, overlap | 3 | 97.63±2.10 | significant (**) |
|  | no overlap | 10 | 1.79±2.15 |  |  | no overlap | 12 | 87.70±11.44 |  |
| CD28^+^T^4-^/Lym | yes, overlap | 5 | 0.57±0.61 |  | CD28^+^T^4-^/T^4-^ | yes, overlap | 4 | 44.80±28.18 |  |
|  | no overlap | 9 | 1.19±1.31 |  |  | no overlap | 11 | 41.91±23.89 |  |
| CD40L^+^T^4+^/Lym | yes, overlap | 3 | 0.45±0.03 |  | CD40L^+^T/T^3+^ | yes, overlap | 2 | 11.60±1.70 |  |
|  | no overlap | 12 | 0.11±0.15 |  |  | no overlap | 5 | 2.55±2.03 |  |
| CD40L^+^T^4-^/Lym | yes, overlap | 3 | 0.19±0.18 | significant (**) | CD40L^+^T^4+^/T^4+^ | yes, overlap | 1 | 19.20 |  |
|  | no overlap | 12 | 0.04±0.06 |  |  | no overlap | 12 | 6.59±7.24 |  |
|  |  |  |  |  | CD40L^+^T^4-^/T^4-^ | yes, overlap | 2 | 3.13±2.30 |  |
|  |  |  |  |  |  | no overlap | 12 | 1.27±1.45 |  |
| T^H1/Gui^/Lym | yes, overlap | 4 | 0.17±0.14 | borderline significant (*) | T^H1/Gui^/T^4+^ | yes, overlap | 4 | 9.53±4.31 | significant (**) |
|  | no overlap | 13 | 0.80±1.25 |  |  | no overlap | 12 | 14.33±8.94 |  |
| T^H2/Gui^/Lym | yes, overlap | 5 | 0.25±0.18 |  | T^H2/Gui^/T^4+^ | yes, overlap | 5 | 8.49±3.85 |  |
|  | no overlap | 13 | 0.40±0.58 |  |  | no overlap | 12 | 7.27±3.23 |  |
| T^H17/Gui^/Lym | yes, overlap | 5 | 0.47±0.56 |  | T^H17/Gui^/T^4+^ | yes, overlap | 5 | 8.34±4.09 |  |
|  | no overlap | 13 | 0.50±0.93 |  |  | no overlap | 10 | 7.93±7.16 |  |
| γδ^+^T/Lym | yes, overlap | 4 | 6.41±5.07 |  | γδ^+^T/T^3+^ | yes, overlap | 4 | 6.41±5.07 |  |
|  | no overlap | 10 | 5.15±4.06 |  |  | no overlap | 10 | 5.15±4.06 |  |
| γδ^+^T^4+^/Lym | yes, overlap | 5 | 0.03±0.02 |  | γδ^+^T^4+^/T^3+^ | yes, overlap | 5 | 1.28±0.84 |  |
|  | no overlap | 10 | 0.02±0.03 |  |  | no overlap | 10 | 0.53±0.70 |  |
| γδ^+^T^4-^/Lym | yes, overlap | 5 | 0.15±0.14 | borderline significant (*) | γδ^+^T^4-^/T^3+^ | yes, overlap | 4 | 5.02±4.18 |  |
|  | no overlap | 10 | 0.45±0.70 |  |  | no overlap | 10 | 4.63±4.18 |  |
| Intß7^+^T/Lym | yes, overlap | 5 | 1.01±0.81 |  | Intß7^+^T/T^3+^ | yes, overlap | 4 | 37.21±17.02 |  |
|  | no overlap | 10 | 1.69±1.44 |  |  | no overlap | 10 | 27.49±14.64 |  |
| Intß7^+^T^4+^/Lym | yes, overlap | 5 | 0.17±0.15 |  | Intß7^+^T^4+^/T^3+^ | yes, overlap | 4 | 19.13±3.07 | borderline significant (*) |
|  | no overlap | 10 | 0.47±0.72 | borderline significant (*) |  | no overlap | 9 | 8.92±5.79 |  |
| Intß7^+^T^4-^/Lym | yes, overlap | 5 | 0.64±0.57 |  | Intß7^+^T^4-^/T^3+^ | yes, overlap | 4 | 22.19±10.49 |  |
|  | no overlap | 10 | 1.23±1.15 |  |  | no overlap | 8 | 22.79±10.83 |  |
| CD127^+^T/Lym | yes, overlap | 5 | 1.19±1.07 |  | CD127^+^T/T^3+^ | yes, overlap | 4 | 55.95±28.01 | significant (**) |
|  | no overlap | 11 | 2.89±2.30 |  |  | no overlap | 9 | 57.66±17.10 |  |
|  |  |  |  |  | T^4+^_reg_ /T^4+^ | yes, overlap | 3 | 11.87±2.12 |  |
|  |  |  |  |  |  | no overlap | 10 | 6.75±4.09 |  |
| T^4+^_reg_/Lym | yes, overlap | 4 | 0.13±0.07 |  | T^4+^_reg_ /T^3+^ | yes, overlap | 4 | 5.91±2.42 |  |
|  | no overlap | 11 | 0.15±0.13 |  |  | no overlap | 11 | 2.44±2.04 |  |
|  |  |  |  |  | T^4-^_reg_ /T^4-^ | yes, overlap | 4 | 0.33±0.31 | borderline significant (*) |
|  |  |  |  |  |  | no overlap | 11 | 1.20±1.62 |  |
| T^4-^_reg_/Lym | yes, overlap | 4 | 0.00±0.00 |  | T^4-^_reg_ /T^3+^ | yes, overlap | 4 | 0.20±0.19 |  |
|  | no overlap | 11 | 0.05±0.12 |  |  | no overlap | 11 | 0.63±0.76 |  |
| CTLA-4^+^T/Lym | yes, overlap | 0^a^ |  |  | CTLA-4^+^T/T^3+^ | yes, overlap | 0^a^ |  | significant (**) |
|  | no overlap | 1 | 0.20 |  |  | no overlap | 1 | 0.92 |  |
| CTLA-4^+^T^4+^/Lym | yes, overlap | 5 | 0.01±0.01 |  | CTLA-4^+^T^4+^/T^4+^ | yes, overlap | 5 | 1.07±0.92 | significant (**) |
|  | no overlap | 12 | 0.02±0.02 |  |  | no overlap | 11 | 0.50±0.34 |  |
| CTLA-4^+^T^4-^/Lym | yes, overlap | 5 | 0.01±0.01 | significant (**) | CTLA-4^+^T^4-^/T^4-^ | yes, overlap | 5 | 0.80±0.44 | significant (**) |
|  | no overlap | 12 | 0.05±0.05 |  |  | no overlap | 12 | 1.24±1.13 |  |
| CD28^+^CTLA-4^+^T/Lym | yes, overlap | 0^a^ |  |  | CD28^+^CTLA-4^+^T^4-^/T^4-^ | yes, overlap | 4 | 0.79±0.63 |  |
|  | no overlap | 1 | 0.11 |  |  | no overlap | 11 | 0.43±0.30 |  |
| CD28^+^CTLA-4^+^T^4+^/Lym | yes, overlap | 5 | 0.01±0.01 |  | CD28^+^CTLA-4^+^T^4+^/T^4+^ | yes, overlap | 5 | 0.45±0.35 |  |
|  | no overlap | 12 | 0.04±0.10 |  |  | no overlap | 10 | 0.38±0.32 |  |
| CD28^+^CTLA-4^+^T^4-^/Lym | yes, overlap | 5 | 0.01±0.01 | borderline significant (*) | CD28^+^CTLA-4^+^T^4-^/T^4-^ |  |  |  |  |
|  | no overlap | 12 | 0.03±0.03 |  |  |  |  |  |  |
| PD-1^+^T/Lym | yes, overlap | 5 | 1.18±0.89 |  | PD-1^+^T/T^3+^ | yes, overlap | 5 | 18.82±12.21 |  |
|  | no overlap | 12 | 1.26±2.24 |  |  | no overlap | 10 | 19.28±10.66 |  |
| PD-1^+^T^4+^/Lym | yes, overlap | 5 | 0.11±0.11 |  | PD-1^+^T^4+^/T^4+^ | yes, overlap | 5 | 8.87±5.55 |  |
|  | no overlap | 12 | 0.16±0.20 |  |  | no overlap | 9 | 14.92±7.07 |  |
| PD-1^+^T^4-^/Lym | yes, overlap | 5 | 1.08±0.83 |  | PD-1^+^T^4-^/T^4-^ | yes, overlap | 5 | 91.14±5.56 |  |
|  | no overlap | 12 | 1.10±2.05 |  |  | no overlap | 10 | 82.88±9.60 |  |
| PD-L1^+^T/Lym | yes, overlap | 5 | 1.30±1.44 | highly significant (***) | PD-L1^+^T/T^3+^ | yes, overlap | 5 | 11.58±3.46 |  |
|  | no overlap | 12 | 0.43±0.46 |  |  | no overlap | 10 | 11.46±6.22 |  |
| PD-L1^+^T^4+^/Lym | yes, overlap | 5 | 0.68±0.76 | significant (**) | PD-L1^+^T^4+^/T^4+^ | yes, overlap | 3 | 47.63±1.87 | significant (**) |
|  | no overlap | 12 | 0.31±0.40 |  |  | no overlap | 11 | 66.00±20.14 |  |
| PD-L1^+^T^4-^/Lym | yes, overlap | 5 | 0.62±0.71 | highly significant (***) | PD-L1^+^T^4-^/T^4-^ | yes, overlap | 2 | 53.05±2.05 | borderline significant (*) |
|  | no overlap | 12 | 0.12±0.13 |  |  | no overlap | 11 | 34.00±20.15 |  |
| LAG3^+^T/Lym | yes, overlap | 0^a^ |  |  | LAG3^+^T/T^3+^ | yes, overlap | 0^a^ |  |  |
|  | no overlap | 2 | 0.11±0.05 |  |  | no overlap | 0^a^ |  |  |
| LAG3^+^T^4+^/Lym | yes, overlap | 4 | 0.03±0.02 |  | LAG3^+^T^4+^/T^4+^ | yes, overlap | 3 | 0.75±0.69 |  |
|  | no overlap | 13 | 0.02±0.02 |  |  | no overlap | 12 | 0.85±0.46 |  |
| LAG3^+^T^4-^/Lym | yes, overlap | 4 | 0.03±0.04 |  | LAG3^+^T^4-^/T^4-^ | yes, overlap | 4 | 1.19±1.04 |  |
|  | no overlap | 13 | 0.06±0.09 |  |  | no overlap | 12 | 0.92±0.65 |  |
| 2B4^+^T/Lym | yes, overlap | 2 | 1.08±0.85 |  | 2B4^+^T/T^3+^ | yes, overlap | 2 | 34.95±3.32 | borderline significant (*) |
|  | no overlap | 8 | 2.88±2.94 |  |  | no overlap | 8 | 37.01±15.86 |  |
| 2B4^+^T^4+^/Lym | yes, overlap | 5 | 0.03±0.03 |  | 2B4^+^T^4+^/T^4+^ | yes, overlap | 5 | 5.88±10.15 | significant (**) |
|  | no overlap | 12 | 0.17±0.34 |  |  | no overlap | 10 | 1.73±1.37 |  |
| 2B4^+^T^4-^/Lym | yes, overlap | 5 | 0.44±0.67 | significant (**) | 2B4^+^T^4-^/T^4-^ | yes, overlap | 3 | 32.60±21.13 |  |
|  | no overlap | 10 | 1.20±1.33 |  |  | no overlap | 9 | 46.74±17.08 |  |
| TIM3^+^T/Lym | yes, overlap | 3 | 0.21±0.03 | highly significant (***) | TIM3^+^T/T^3+^ | yes, overlap | 2 | 10.17±4.71 |  |
|  | no overlap | 9 | 0.38±0.20 |  |  | no overlap | 8 | 5.01±5.21 |  |
| TIM3^+^T^4+^/Lym | yes, overlap | 5 | 0.01±0.01 | significant (**) | TIM3^+^T^4+^/T^4+^ | yes, overlap | 5 | 1.92±1.13 |  |
|  | no overlap | 12 | 0.05±0.04 |  |  | no overlap | 10 | 1.46±0.84 |  |
| TIM3^+^T^4-^/Lym | yes, overlap | 5 | 0.14±0.07 | highly significant (***) | TIM3^+^T^4-^/T^4-^ | yes, overlap | 4 | 8.50±6.58 |  |
|  | no overlap | 12 | 0.25±0.19 |  |  | no overlap | 11 | 5.15±5.24 |  |
| KLRG1^+^T/Lym | yes, overlap | 4 | 1.40±2.18 |  | KLRG1^+^T/T^3+^ | yes, overlap | 3 | 29.67±25.40 |  |
|  | no overlap | 12 | 1.74±2.53 |  |  | no overlap | 10 | 36.91±16.93 |  |
| KLRG1^+^T^4+^/Lym | yes, overlap | 5 | 0.34±0.46 |  | KLRG1^+^T^4+^/T^4+^ | yes, overlap | 5 | 14.51±6.87 | significant (**) |
|  | no overlap | 12 | 0.22±0.45 |  |  | no overlap | 10 | 12.28±12.51 |  |
| KLRG1^+^T^4-^/Lym | yes, overlap | 5 | 2.39±3.08 |  | KLRG1^+^T^4-^/T^4-^ | yes, overlap | 5 | 85.48±6.86 | significant (**) |
|  | no overlap | 12 | 1.52±2.29 |  |  | no overlap | 11 | 84.37±16.28 |  |
| TIGIT^+^T/Lym | yes, overlap | 4 | 2.43±2.44 |  | TIGIT^+^T/T^3+^ | yes, overlap | 5 | 28.04±9.71 |  |
|  | no overlap | 11 | 1.33±1.66 |  |  | no overlap | 10 | 37.60±13.77 |  |
| TIGIT^+^T^4+^/Lym | yes, overlap | 5 | 0.65±0.68 |  | TIGIT^+^T^4+^/T^4+^ | yes, overlap | 5 | 80.14±14.01 | significant (**) |
|  | no overlap | 12 | 0.62±0.84 |  |  | no overlap | 10 | 59.99±27.86 |  |
| TIGIT^+^T^4-^/Lym | yes, overlap | 5 | 2.88±2.61 |  | TIGIT^+^T^4-^/T^4-^ | yes, overlap | 5 | 19.87±13.99 | significant (**) |
|  | no overlap | 12 | 1.37±2.30 |  |  | no overlap | 10 | 40.27±28.39 |  |
| B/Lym | yes, overlap | 5 | 0.05±0.10 |  |  |  |  |  |  |
|  | no overlap | 10 | 0.04±0.04 |  |  |  |  |  |  |
| B_nai_/Lym | yes, overlap | 4 | 5.32±9.99 | significant (**) | B_nai_/B | yes, overlap | 4 | 50.30±30.46 |  |
|  | no overlap | 10 | 3.31±4.07 |  |  | no overlap | 8 | 66.40±21.66 |  |
| B_mem(class)_/Lym | yes, overlap | 4 | 0.35±0.32 |  | B_mem(class)/_B | yes, overlap | 4 | 11.95±10.54 |  |
|  | no overlap | 10 | 0.46±0.58 |  |  | no overlap | 10 | 17.47±12.56 |  |
| B_mem_/Lym | yes, overlap | 5 | 0.08±0.14 |  | B_mem_/B | yes, overlap | 5 | 3.26±3.14 |  |
|  | no overlap | 10 | 0.22±0.33 |  |  | no overlap | 10 | 5.04±5.11 |  |
| B_reg_/Lym | yes, overlap | 0^a^ |  |  | B_reg_/B | yes, overlap | 5 | 2.12±2.19 |  |
|  | no overlap | 0^a^ |  |  |  | no overlap | 10 | 1.63±2.09 |  |
| Innate cells | | | | | | | | | |
| In Lym-Gate | | | | | In subsets | | | | |
| Overlap cGvHD | Yes/no | N | % Mean  SD | p | Overlap cGvHD | Yes/no | N | % Mean  SD | p |
| NK^56+^/Lym | yes, overlap | 5 | 0.08±0.06 |  |  |  |  |  |  |
|  | no overlap | 11 | 0.05±0.06 |  |  |  |  |  |  |
| NK^56bri^/Lym | yes, overlap | 5 | 2.25±3.16 |  | NK^56bri^/NK^56+^ | yes, overlap | 4 | 17.26±11.51 |  |
|  | no overlap | 9 | 1.43±2.77 |  |  | no overlap | 10 | 13.91±14.24 |  |
| NK^56bri16-^/Lym | yes, overlap | 5 | 1.58±2.59 |  | NK^56bri16-^/NK^56+^ | yes, overlap | 5 | 14.45±14.55 | significant (**) |
|  | no overlap | 10 | 1.61±3.18 |  |  | no overlap | 10 | 4.05±3.61 |  |
| NK^56bri16+^/Lym | yes, overlap | 5 | 0.67±0.65 |  | NK^56bri16+^/NK^56+^ | yes, overlap | 5 | 8.57±2.75 |  |
|  | no overlap | 10 | 2.43±5.55 |  |  | no overlap | 9 | 6.44±6.48 |  |
| NK^56dim16+^/Lym | yes, overlap | 5 | 3.22±2.88 | borderline significant (*) | NK^56dim16+^/NK^56+^ | yes, overlap | 4 | 55.83±13.34 |  |
|  | no overlap | 10 | 15.66±27.53 |  |  | no overlap | 10 | 73.87±21.05 |  |
| NK^56bri57-^/Lym | yes, overlap | 5 | 2.19±3.06 |  | NK^56bri57-^/NK^56+^ | yes, overlap | 4 | 16.96±11.10 |  |
|  | no overlap | 9 | 1.50±2.82 |  |  | no overlap | 10 | 14.37±14.76 |  |
| NK^56dim57-^/Lym | yes, overlap | 5 | 3.63±2.63 |  | NK^56dim57-^/NK^56+^ | yes, overlap | 5 | 51.82±19.88 |  |
|  | no overlap | 8 | 2.76±4.89 |  |  | no overlap | 9 | 47.06±14.52 |  |
| NK^56dim57+^/Lym | yes, overlap | 5 | 1.69±1.52 |  | NK^56dim57+^/NK^56+^ | yes, overlap | 5 | 24.67±15.04 |  |
|  | no overlap | 10 | 3.84±5.21 |  |  | no overlap | 8 | 26.30±19.28 |  |
| NK^56dim57-16bri^/Lym | yes, overlap | 5 | 1.36±1.36 | significant (**) | NK^56dim57-16bri^/NK^56+^ | yes, overlap | 5 | 20.82±12.91 |  |
|  | no overlap | 10 | 6.51±10.38 |  |  | no overlap | 10 | 25.82±13.12 |  |
| NK^56dim57+16+^/Lym | yes, overlap | 5 | 1.68±1.51 |  | NK^56dim57+16+^/NK^56+^ | yes, overlap | 5 | 24.56±15.10 | borderline significant (*) |
|  | no overlap | 10 | 3.82±5.21 |  |  | no overlap | 9 | 31.20±23.39 |  |
| CTLA-4^+^NK^56+^/Lym | yes, overlap | 5 | 0.65±0.88 |  | CTLA-4^+^NK^56+^/NK^56+^ | yes, overlap | 4 | 42.98±13.84 |  |
|  | no overlap | 12 | 1.29±1.28 |  |  | no overlap | 11 | 29.85±14.36 |  |
| PD-1^+^NK^56+^/Lym | yes, overlap | 5 | 0.01±0.01 | significant (**) | PD-1^+^NK^56+^/NK^56+^ | yes, overlap | 4 | 19.69±18.06 |  |
|  | no overlap | 13 | 0.07±0.08 |  |  | no overlap | 13 | 11.47±9.11 |  |
| PD-L1^+^NK^56+^/Lym | yes, overlap | 5 | 0.04±0.04 |  | PD-L1^+^NK^56+^/NK^56+^ | yes, overlap | 3 | 33.67±11.65 |  |
|  | no overlap | 12 | 0.07±0.09 |  |  | no overlap | 13 | 12.99±11.38 |  |
| PD-L2^+^NK^56+^/Lym | yes, overlap | 4 | 0.06±0.09 |  | PD-L2^+^NK^56+^/NK^56+^ | yes, overlap | 4 | 1.85±0.88 |  |
|  | no overlap | 13 | 0.05±0.06 |  |  | no overlap | 13 | 1.00±1.00 |  |
| CD40L^+^NK^56+^/Lym | yes, overlap | 5 | 0.11±0.10 |  | CD40L^+^NK^56+^/NK^56+^ | yes, overlap | 5 | 16.75±13.39 |  |
|  | no overlap | 13 | 0.09±0.13 |  |  | no overlap | 12 | 16.10±15.42 |  |
| LAG3^+^NK^56+^/Lym | yes, overlap | 4 | 0.04±0.06 | borderline significant (*) | LAG3^+^NK^56+^/NK^56+^ | yes, overlap | 2 | 71.60±6.93 |  |
|  | no overlap | 13 | 0.03±0.03 |  |  | no overlap | 13 | 91.70±7.30 |  |
| 2B4^+^NK^56+^/Lym | yes, overlap | 5 | 1.11±0.62 | borderline significant (*) | 2B4^+^NK^56+^/NK^56+^ | yes, overlap | 4 | 55.38±15.16 |  |
|  | no overlap | 10 | 4.04±2.96 |  |  | no overlap | 10 | 69.60±19.18 |  |
| TIM3^+^NK^56+^/Lym | yes, overlap | 5 | 1.04±0.61 |  |  |  |  |  |  |
|  | no overlap | 10 | 1.86±1.41 |  |  |  |  |  |  |
| KLRG1^+^NK^56+^/Lym | yes, overlap | 5 | 1.07±1.20 |  | KLRG1^+^NK^56+^/NK^56+^ | yes, overlap | 5 | 25.76±19.91 |  |
|  | no overlap | 13 | 1.17±1.12 |  |  | no overlap | 12 | 22.28±12.65 |  |
| TIGIT^+^NK^56+^/Lym | yes, overlap | 5 | 1.35±2.01 |  | TIGIT^+^NK^56+^/NK^56+^ | yes, overlap | 4 | 35.03±18.71 |  |
|  | no overlap | 12 | 1.92±1.54 |  |  | no overlap | 11 | 29.26±12.37 |  |
| CIK^56+^/Lym | yes, overlap | 5 | 0.65±0.38 |  |  |  |  |  |  |
|  | no overlap | 13 | 0.24±0.31 |  |  |  |  |  |  |
| CD40L^+^CIK^56+^/Lym | yes, overlap | 5 | 0.25±0.34 |  | CD40L^+^CIK^56+^/CIK^56+^ | yes, overlap | 5 | 15.11±10.87 |  |
|  | no overlap | 13 | 0.22±0.26 |  |  | no overlap | 12 | 7.78±11.47 |  |
| MAIT cells /Lym | yes, overlap | 4 | 0.00±0.00 | significant (**) |  |  |  |  |  |
|  | no overlap | 13 | 0.01±0.01 |  |  |  |  |  |  |
| CTLA-4^+^CIK^56+^/Lym | yes, overlap | 5 | 0.08±0.05 | borderline significant (*) | CTLA-4^+^CIK^56+^/CIK^56+^ | yes, overlap | 5 | 13.28±5.06 | borderline significant (*) |
|  | no overlap | 13 | 0.03±0.03 |  |  | no overlap | 11 | 17.32±12.20 |  |
| PD-1^+^CIK^56+^/Lym | yes, overlap | 5 | 0.14±0.26 |  | PD-1^+^CIK^56+^/CIK^56+^ | yes, overlap | 5 | 5.85±3.32 |  |
|  | no overlap | 13 | 0.17±0.26 |  |  | no overlap | 12 | 4.93±4.70 |  |
| PD-L1^+^CIK^56+^/Lym | yes, overlap | 5 | 0.31±0.41 |  | PD-L1^+^CIK^56+^/CIK^56+^ | yes, overlap | 4 | 18.05±13.36 | significant (**) |
|  | no overlap | 13 | 0.25±0.28 |  |  | no overlap | 11 | 7.12±5.20 |  |
| PD-L2^+^CIK^56+^/Lym | yes, overlap | 5 | 0.16±0.26 | highly significant (***) | PD-L2^+^CIK^56+^/CIK^56+^ | yes, overlap | 5 | 12.15±8.63 |  |
|  | no overlap | 13 | 0.01±0.02 |  |  | no overlap | 12 | 8.55±10.74 |  |
| KLRG1^+^CIK^56+^/Lym | yes, overlap | 5 | 0.35±0.69 | highly significant (***) | KLRG1^+^CIK^56+^/CIK^56+^ | yes, overlap | 3 | 36.53±31.38 |  |
|  | no overlap | 13 | 0.10±0.13 |  |  | no overlap | 12 | 65.52±21.62 |  |
| TIGIT^+^CIK^56+^/Lym | yes, overlap | 5 | 0.26±0.49 | significant (**) | TIGIT^+^CIK^56+^/CIK^56+^ | yes, overlap | 3 | 14.89±8.04 |  |
|  | no overlap | 13 | 0.10±0.17 |  |  | no overlap | 11 | 38.87±11.14 |  |
| iNKT/Lym | yes, overlap | 5 | 0.23±0.05 |  |  |  |  |  |  |
|  | no overlap | 11 | 0.22±0.43 |  |  |  |  |  |  |
| iNKT^3+^/Lym | yes, overlap | 5 | 0.02±0.02 |  |  |  |  |  |  |
|  | no overlap | 11 | 0.11±0.33 |  |  |  |  |  |  |
| iNKT^161+^/Lym | yes, overlap | 5 | 0.13±0.06 |  |  |  |  |  |  |
|  | no overlap | 11 | 0,.0±0,.7 |  |  |  |  |  |  |

# **Supplementary Table 7 — NoGvHD at baseline → developed GvHD**

| Myeloid Cells | | | | | | | | | |
| --- | --- | --- | --- | --- | --- | --- | --- | --- | --- |
| In non Lym-Gate | | | | | In subsets | | | | |
| GvHD after baseline /no GvHD | yes/no | N | % Mean  SD | *p* | GvHD after baseline /no GvHD | yes/no | N | % Mean  SD | *p* |
| CD14^+^/non Lym | yes | 4 | 6.20±2.81 |  |  |  |  |  |  |
|  | no | 14 | 8.54±3.89 |  |  |  |  |  |  |
| CTLA-4^+^Mo/non Lym | yes | 4 | 0.96±0.80 |  | CTLA-4^+^Mo/Mo | yes | 4 | 35.83±18.36 |  |
|  | no | 14 | 1.49±1.42 |  |  | no | 14 | 37.17±17.64 |  |
| PD-1^+^Mo/non Lym | yes | 4 | 0.90±0.43 |  | PD-1^+^Mo/Mo | yes | 4 | 15.68±2.42 |  |
|  | no | 14 | 1.15±1.06 |  |  | no | 15 | 15.70±10.99 |  |
| PD-L1^+^Mo/non Lym | yes | 4 | 5.70±2.76 |  | PD-L1^+^Mo/Mo | yes | 4 | 98.60±1.00 |  |
|  | no | 14 | 7.85±4.67 |  |  | no | 14 | 97.55±3.30 |  |
| LAG3^+^Mo/non Lym | yes | 4 | 0.22±0.16 | borderline significant (*) | LAG3^+^Mo/Mo | yes | 4 | 4.02±1.75 |  |
|  | no | 15 | 0.14±0.07 |  |  | no | 15 | 1.89±1.33 |  |
| 2B4^+^Mo/non Lym | yes | 4 | 6.27±3.12 |  | 2B4^+^Mo/Mo | yes | 4 | 99.78±0.15 |  |
|  | no | 14 | 8.50±3.98 |  |  | no | 15 | 99.63±0.71 |  |
| Tim3^+^Mo/non Lym | yes | 4 | 5.44±2.64 |  | Tim3^+^Mo/Mo | yes | 4 | 88.03±9.69 |  |
|  | no | 14 | 7.09±3.53 |  |  | no | 15 | 83.77±9.00 |  |
| DC^80+^/non Lym | yes | 4 | 2.03±1.06 |  |  |  |  |  |  |
|  | no | 15 | 0.91±0.53 |  |  |  |  |  |  |
| DC_mat_^80+^/non Lym | yes | 4 | 0.36±0.17 |  | DC_mat_^80+^/DC^80+^ | yes | 4 | 30.03±16.30 |  |
|  | no | 15 | 0.33±0.20 |  |  | no | 13 | 31.98±10.53 |  |
| DC_tol_^80+^/non Lym | yes | 4 | 0.79±0.74 | significant (**) | DC_tol_^80+^/DC^80+^ | yes | 2 | 60.90±2.12 | borderline significant (*) |
|  | no | 15 | 0.44±0.32 |  |  | no | 15 | 47.24±14.20 |  |
| CTLA-4^+^DC^80+^/non Lym | yes | 4 | 0.54±0.31 |  | CTLA-4^+^DC^80+^/DC^80+^ | yes | 3 | 25.07±19.11 | significant (**) |
|  | no | 15 | 0.33±0.19 |  |  | no | 14 | 36.46±8.35 |  |
| CTLA-4^+^DC_mat_^80+^/non Lym | yes | 4 | 0.26±0.05 |  | CTLA-4^+^DC_mat_^80+^/DC_mat_^80+^ | yes | 3 | 90.13±5.66 |  |
|  | no | 15 | 0.26±0.16 |  |  | no | 15 | 80.72±10.36 |  |
| PMN-MDSC/non Lym | yes | 4 | 18.40±2.44 | borderline significant (*) | PMN-MDSC/CD11b+CD33^+^ | yes | 4 | 73.45±10.39 |  |
|  | no | 14 | 18.01±9.55 |  |  | no | 13 | 70.61±7.93 |  |
| M-MDSC/non Lym | yes | 4 | 6.86±3.10 |  | M-MDSC/CD11b+CD33^+^ | yes | 4 | 26.55±10.39 |  |
|  | no | 14 | 8.94±4.05 |  |  | no | 14 | 31.86±11.98 |  |
| CTLA-4^+^PMN-MDSC/non Lym | yes | 4 | 4.47±1.35 |  | CTLA-4^+^PMN-MDSC/PMN-MDSC | yes | 4 | 18.66±10.65 |  |
|  | no | 15 | 2.51±1.06 |  |  | no | 14 | 16.70±9.96 |  |
| CTLA-4^+^M-MDSC/non Lym | yes | 4 | 1.63±0.97 |  | CTLA-4^+^M-MDSC/PMN-MDSC | yes | 4 | 19.60±7.29 |  |
|  | no | 14 | 1.88±1.59 |  |  | no | 12 | 17.39±7.82 |  |
| Adaptive Cells | | | | | | | | | |
| In Lym-Gate | | | | | In subsets | | | | |
| GvHD after baseline /no GvHD | yes/no | N | % Mean  SD | *p* | GvHD after baseline /no GvHD | yes/no | N | % Mean  SD | *p* |
| T/Lym | yes | 4 | 8.52±3.20 | significant (**) |  |  |  |  |  |
|  | no | 14 | 12.01±7.84 |  |  |  |  |  |  |
| T^4+^/Lym | yes | 4 | 4.94±3.51 |  | T^4+^/T^3+^ | yes | 4 | 23.98±20.22 |  |
|  | no | 15 | 3.61±2.24 |  |  | no | 13 | 33.82±16.24 |  |
| T^4-/^Lym | yes | 4 | 17.22±7.83 |  | T^4-^/T^3+^ | yes | 4 | 76.03±20.22 |  |
|  | no | 14 | 7.14±6.47 |  |  | no | 13 | 69.14±17.20 |  |
| T_prol-early_ /Lym | yes | 4 | 2.82±3.27 | highly significant (***) | T_prol-early_ /T^3+^ | yes | 3 | 5.96±1.45 |  |
|  | no | 15 | 0.64±0.54 |  |  | no | 14 | 3.66±2.17 |  |
| T_prol-late_ /Lym | yes | 4 | 2.07±2.49 | highly significant (***) | T_prol-late_ /T^3+^ | yes | 3 | 4.73±2.38 |  |
|  | no | 15 | 0.98±0.59 |  |  | no | 12 | 5.39±3.50 |  |
| T_nn_ /Lym | yes | 3 | 8.30±2.88 |  | T_nn_ /T^3+^ | yes | 3 | 66.27±14.10 |  |
|  | no | 14 | 6.45±5.24 |  |  | no | 13 | 66.58±9.89 |  |
| T_em_ /Lym | yes | 4 | 99.10±1.09 | borderline significant (*) | T_em_ /T^3+^ | yes | 3 | 65.63±14.47 |  |
|  | no | 12 | 83.03±31.39 |  |  | no | 14 | 65.53±15.58 |  |
| T_cm_ /Lym | yes | 4 | 0.89±1.08 |  | T_cm_ /T^3+^ | yes | 4 | 0.49±0.69 |  |
|  | no | 14 | 2.00±2.47 |  |  | no | 15 | 1.77±1.47 |  |
| CD28^+^T/Lym | yes | 4 | 8.74±5.19 |  | CD28^+^T/T^3+^ | yes | 2 | 67.90±20.93 |  |
|  | no | 14 | 8.05±5.96 |  |  | no | 13 | 75.55±18.88 |  |
| CD28^+^T^4+^/Lym | yes | 4 | 4.31±2.91 |  | CD28^+^T^4+^/T^4+^ | yes | 4 | 91.08±8.30 |  |
|  | no | 15 | 4.33±2.64 |  |  | no | 15 | 95.53±5.45 |  |
| CD28^+^T^4-^/Lym | yes | 4 | 4.54±4.04 |  | CD28^+^T^4-^/T^4-^ | yes | 3 | 42.87±21.25 |  |
|  | no | 14 | 4.06±4.07 |  |  | no | 14 | 59.37±22.39 |  |
| CD40L^+^T^4+^/Lym | yes | 4 | 0.13±0.05 |  | CD40L^+^T/T^3+^ | yes | 4 | 1.26±0.36 | borderline significant (*) |
|  | no | 15 | 0.23±0.29 |  |  | no | 14 | 2.54±2.47 |  |
| CD40L^+^T^4-^/Lym | yes | 4 | 0.16±0.13 |  | CD40L^+^T^4+^/T^4+^ | yes | 4 | 3.09±1.21 | borderline significant (*) |
|  | no | 15 | 0.12±0.16 |  |  | no | 14 | 3.94±3.00 |  |
|  |  |  |  |  | CD40L^+^T^4-^/T^4-^ | yes | 4 | 0.84±0.42 |  |
|  |  |  |  |  |  | no | 13 | 1.31±1.51 |  |
| T^H1/Gui^/Lym | yes | 4 | 1.15±0.68 |  | T^H1/Gui^/T^4+^ | yes | 2 | 8.42±0.36 | borderline significant (*) |
|  | no | 15 | 0.84±0.53 |  |  | no | 14 | 10.41±3.57 |  |
| T^H2/Gui^/Lym | yes | 4 | 0.47±0.56 |  | T^H2/Gui^/T^4+^ | yes | 4 | 4.14±3.39 |  |
|  | no | 15 | 0.72±0.48 |  |  | no | 13 | 8.73±3.45 |  |
| T^H17/Gui^/Lym | yes | 4 | 1.27±1.27 |  | T^H17/Gui^/T^4+^ | yes | 4 | 14.93±3.85 |  |
|  | no | 15 | 1.22±0.92 |  |  | no | 13 | 13.36±4.14 |  |
| γδ^+^T/Lym | yes | 4 | 0.15±0.08 |  | γδ^+^T/T^3+^ | yes | 3 | 4.26±5.70 | highly significant (***) |
|  | no | 15 | 0.42±0.45 |  |  | no | 14 | 2.40±1.55 |  |
| γδ^+^T^4+^/Lym | yes | 4 | 0.05±0.02 |  | γδ^+^T^4+^/T^3+^ | yes | 4 | 1.49±1.72 | borderline significant (*) |
|  | no | 15 | 0.10±0.11 |  |  | no | 14 | 0.80±0.73 |  |
| γδ^+^T^4-^/Lym | yes | 4 | 0.21±0.20 |  | γδ^+^T^4-^/T^3+^ | yes | 4 | 1.15±1.25 |  |
|  | no | 14 | 0.33±0.46 |  |  | no | 15 | 3.10±5.27 |  |
| Intß7^+^T/Lym | yes | 4 | 5.52±3.91 |  | Intß7^+^T/T^3+^ | yes | 4 | 41.34±19.42 |  |
|  | no | 15 | 6.96±4.27 |  |  | no | 15 | 58.55±17.61 |  |
| Intß7^+^T^4+^/Lym | yes | 4 | 2.10±1.02 |  | Intß7^+^T^4+^/T^3+^ | yes | 4 | 18.36±19.67 |  |
|  | no | 13 | 1.32±0.87 |  |  | no | 12 | 19.77±14.16 |  |
| Intß7^+^T^4-^/Lym | yes | 3 | 5.03±3.74 |  | Intß7^+^T^4-^/T^3+^ | yes | 4 | 13.93±7.15 | borderline significant (*) |
|  | no | 13 | 3.84±3.29 |  |  | no | 12 | 37.81±17.50 |  |
| CD127^+^T/Lym | yes | 3 | 6.16±3.10 |  | CD127^+^T/T^3+^ | yes | 4 | 27.78±13.51 |  |
|  | no | 15 | 7.43±4.99 |  |  | no | 15 | 51.60±14.44 |  |
|  |  |  |  | highly significant (***) | T^4+^_reg_ /T^4+^ | yes | 4 | 8.41±3.35 |  |
|  |  |  |  |  |  | no | 14 | 9.22±3.66 |  |
| T^4+^_reg_/Lym | yes | 4 | 0.48±0.28 |  | T^4+^_reg_ /T^3+^ | yes | 2 | 0.43±0.03 |  |
|  | no | 15 | 0.49±0.30 |  |  | no | 15 | 0.65±1.00 |  |
|  |  |  |  |  | T^4-^_reg_ /T^4-^ | yes | 4 | 1.98±1.39 |  |
|  |  |  |  |  |  | no | 15 | 3.98±2.80 |  |
| T^4-^_reg_/Lym | yes | 4 | 0.75±0.96 |  | T^4-^_reg_ /T^3+^ | yes | 2 | 0.28±0.13 |  |
|  | no | 15 | 0.02±0.02 |  |  | no | 13 | 0.19±0.20 |  |
| CTLA-4^+^T/Lym | yes | 4 | 0.26±0.19 | highly significant (***) | CTLA-4^+^T/T^3+^ | yes | 4 | 1.26±0.63 |  |
|  | no | 15 | 0.15±0.12 |  |  | no | 15 | 1.12±0.95 |  |
| CTLA-4^+^T^4+^/Lym | yes | 4 | 0.11±0.09 |  | CTLA-4^+^T^4+^/T^4+^ | yes | 4 | 2.00±1.31 | highly significant (***) |
|  | no | 15 | 0.02±0.01 |  |  | no | 15 | 0.60±0.43 |  |
| CTLA-4^+^T^4-^/Lym | yes | 4 | 0.11±0.07 | significant (*) | CTLA-4^+^T^4-^/T^4-^ | yes | 4 | 0.70±0.11 | borderline significant (*) |
|  | no | 15 | 0.11±0.09 |  |  | no | 15 | 1.09±0.66 |  |
| CD28^+^CTLA-4^+^T/Lym | yes | 2 | 0.32±0.16 | highly significant (***) | CD28^+^CTLA-4^+^T^4-^/T^4-^ | yes | 4 | 1.96±1.26 | significant (**) |
|  | no | 3 | 0.27±0.06 |  |  | no | 15 | 0.63±0.60 |  |
| CD28^+^CTLA-4^+^T^4+^/Lym | yes | 4 | 0.11±0.09 |  | CD28^+^CTLA-4^+^T^4+^/T^4+^ | yes | 4 | 0.42±0.33 |  |
|  | no | 15 | 0.02±0.01 |  |  | no | 15 | 0.92±0.74 |  |
| CD28^+^CTLA-4^+^T^4-^/Lym | yes | 4 | 0.07±0.08 |  | CD28^+^CTLA-4^+^T^4-^/T^4-^ |  |  |  |  |
|  | no | 15 | 0.09±0.11 |  |  |  |  |  |  |
| PD-1^+^T/Lym | yes | 4 | 3.97±3.46 | significant (*) | PD-1^+^T/T^3+^ | yes | 3 | 17.80±10.12 |  |
|  | no | 15 | 1.87±3.29 |  |  | no | 14 | 9.74±5.86 |  |
| PD-1^+^T^4+^/Lym | yes | 4 | 0.70±0.43 | significant (*) | PD-1^+^T^4+^/T^4+^ | yes | 3 | 15.33±5.82 |  |
|  | no | 15 | 0.20±0.16 |  |  | no | 14 | 17.57±9.89 |  |
| PD-1^+^T^4-^/Lym | yes | 4 | 3.27±3.14 | highly significant (***) | PD-1^+^T^4-^/T^4-^ | yes | 4 | 76.73±16.58 |  |
|  | no | 14 | 0.87±0.65 |  |  | no | 15 | 80.84±11.34 |  |
| PD-L1^+^T/Lym | yes | 4 | 1.46±0.13 | borderline significant (*) | PD-L1^+^T/T^3+^ | yes | 3 | 7.15±3.68 |  |
|  | no | 15 | 0.62±0.37 |  |  | no | 15 | 6.10±5.10 |  |
| PD-L1^+^T^4+^/Lym | yes | 4 | 0.86±0.37 |  | PD-L1^+^T^4+^/T^4+^ | yes | 3 | 67.33±14.68 |  |
|  | no | 15 | 0.46±0.31 |  |  | no | 15 | 73.38±18.20 |  |
| PD-L1^+^T^4-^/Lym | yes | 4 | 0.60±0.27 | borderline significant (*) | PD-L1^+^T^4-^/T^4-^ | yes | 4 | 42.23±22.57 |  |
|  | no | 15 | 0.16±0.15 |  |  | no | 12 | 32.43±15.25 |  |
| LAG3^+^T/Lym | yes | 4 | 0.12±0.07 |  | LAG3^+^T/T^3+^ | yes | 4 | 0.56±0.22 |  |
|  | no | 15 | 0.07±0.05 |  |  | no | 15 | 0.59±0.50 |  |
| LAG3^+^T^4+^/Lym | yes | 4 | 0.08±0.05 |  | LAG3^+^T^4+^/T^4+^ | yes | 3 | 1.52±0.43 |  |
|  | no | 15 | 0.05±0.03 |  |  | no | 14 | 0.99±0.50 |  |
| LAG3^+^T^4-^/Lym | yes | 4 | 0.12±0.12 |  | LAG3^+^T^4-^/T^4-^ | yes | 4 | 0.65±0.23 |  |
|  | no | 15 | 0.07±0.06 |  |  | no | 14 | 0.70±0.35 |  |
| 2B4^+^T/Lym | yes | 4 | 6.73±5.10 |  | 2B4^+^T/T^3+^ | yes | 4 | 31.84±23.32 |  |
|  | no | 14 | 2.70±3.48 |  |  | no | 15 | 17.93±13.64 |  |
| 2B4^+^T^4+^/Lym | yes | 4 | 0.16±0.07 | borderline significant (*) | 2B4^+^T^4+^/T^4+^ | yes | 3 | 2.91±1.01 |  |
|  | no | 15 | 0.07±0.03 |  |  | no | 14 | 1.46±0.72 |  |
| 2B4^+^T^4-^/Lym | yes | 4 | 6.83±5.28 |  | 2B4^+^T^4-^/T^4-^ | yes | 3 | 48.87±15.82 |  |
|  | no | 14 | 2.67±3.51 |  |  | no | 13 | 29.23±13.46 |  |
| TIM3^+^T/Lym | yes | 4 | 0.85±1.10 | significant (**) | TIM3^+^T/T^3+^ | yes | 4 | 3.64±3.09 |  |
|  | no | 14 | 0.54±0.46 |  |  | no | 14 | 3.64±1.61 |  |
| TIM3^+^T^4+^/Lym | yes | 4 | 0.06±0.04 |  | TIM3^+^T^4+^/T^4+^ | yes | 4 | 1.28±0.49 |  |
|  | no | 15 | 0.06±0.04 |  |  | no | 15 | 1.43±0.76 |  |
| TIM3^+^T^4-^/Lym | yes | 4 | 0.80±1.11 | significant (**) | TIM3^+^T^4-^/T^4-^ | yes | 4 | 4.47±3.87 |  |
|  | no | 15 | 0.45±0.45 |  |  | no | 14 | 5.38±3.20 |  |
| KLRG1^+^T/Lym | yes | 4 | 8.58±6.66 |  | KLRG1^+^T/T^3+^ | yes | 3 | 48.60±9.13 |  |
|  | no | 15 | 6.09±5.56 |  |  | no | 14 | 40.52±14.09 |  |
| KLRG1^+^T^4+^/Lym | yes | 4 | 0.31±0.55 |  | KLRG1^+^T^4+^/T^4+^ | yes | 4 | 3.46±3.45 |  |
|  | no | 15 | 0.64±0.51 |  |  | no | 14 | 11.80±7.18 |  |
| KLRG1^+^T^4-^/Lym | yes | 4 | 8.26±6.24 |  | KLRG1^+^T^4-^/T^4-^ | yes | 4 | 96.55±3.43 |  |
|  | no | 15 | 5.45±5.20 |  |  | no | 14 | 88.20±7.18 |  |
| TIGIT^+^T/Lym | yes | 4 | 10.45±5.21 |  | TIGIT^+^T/T^3+^ | yes | 4 | 49.15±14.00 |  |
|  | no | 15 | 7.35±7.76 |  |  | no | 15 | 44.65±16.36 |  |
| TIGIT^+^T^4+^/Lym | yes | 4 | 1.69±0.97 |  | TIGIT^+^T^4+^/T^4+^ | yes | 4 | 17.76±13.94 |  |
|  | no | 15 | 1.23±0.87 |  |  | no | 13 | 22.94±13.64 |  |
| TIGIT^+^T^4-^/Lym | yes | 4 | 8.78±4.98 |  | TIGIT^+^T^4-^/T^4-^ | yes | 4 | 17.76±13.94 |  |
|  | no | 14 | 4.98±5.99 |  |  | no | 13 | 22.94±13.64 |  |
| B/Lym | yes | 4 | 0.04±0.03 |  |  |  |  |  |  |
|  | no | 15 | 0.10±0.20 |  |  |  |  |  |  |
| B_nai_/Lym | yes | 4 | 3.53±2.69 |  | B_nai_/B | yes | 4 | 81.98±9.68 |  |
|  | no | 14 | 3.82±3.43 |  |  | no | 15 | 75.52±19.81 |  |
| B_mem(class)_/Lym | yes | 4 | 0.43±0.50 |  | B_mem(class)/_B | yes | 4 | 8.18±4.49 |  |
|  | no | 15 | 0.31±0.26 |  |  | no | 15 | 11.18±14.48 |  |
| B_mem_/Lym | yes | 4 | 0.06±0.04 |  | B_mem_/B | yes | 4 | 1.50±0.89 |  |
|  | no | 15 | 0.09±0.06 |  |  | no | 15 | 2.45±2.23 |  |
| B_reg_/Lym | yes | 4 | 0.32±0.22 |  | B_reg_/B | yes | 4 | 7.14±4.82 |  |
|  | no | 15 | 0.23±0.16 |  |  | no | 15 | 5.80±4.05 |  |
| Innate Cells | | | | | | | | | |
| In Lym-Gate | | | | | In subsets | | | | |
| GvHD after baseline /no GvHD | yes/no | N | % Mean  SD | *p* | GvHD after baseline /no GvHD | yes/no | N | % Mean  SD | *p* |
| NK^56+^/Lym | yes | 4 | 0.41±0.73 |  |  |  |  |  |  |
|  | no | 15 | 0.45±1.18 |  |  |  |  |  |  |
| NK^56bri^/Lym | yes | 4 | 0.61±0.44 |  | NK^56bri^/NK^56+^ | yes | 4 | 15.72±7.48 |  |
|  | no | 13 | 1.79±1.88 |  |  | no | 13 | 16.46±9.64 |  |
| NK^56bri16-^/Lym | yes | 4 | 0.31±0.19 |  | NK^56bri16-^/NK^56+^ | yes | 4 | 5.97±4.98 |  |
|  | no | 13 | 0.53±0.39 |  |  | no | 13 | 8.86±7.47 |  |
| NK^56bri16+^/Lym | yes | 4 | 0.66±0.46 |  | NK^56bri16+^/NK^56+^ | yes | 4 | 9.75±2.83 |  |
|  | no | 13 | 1.08±1.29 |  |  | no | 13 | 10.61±8.37 |  |
| NK^56dim16+^/Lym | yes | 4 | 5.17±3.71 |  | NK^56dim16+^/NK^56+^ | yes | 4 | 75.70±10.39 |  |
|  | no | 14 | 7.39±4.10 |  |  | no | 12 | 78.74±9.89 |  |
| NK^56bri57-^/Lym | yes | 4 | 0.97±0.46 |  | NK^56bri57-^/NK^56+^ | yes | 4 | 16.35±9.28 |  |
|  | no | 13 | 1.91±2.09 |  |  | no | 11 | 13.61±5.85 |  |
| NK^56dim57-^/Lym | yes | 4 | 3.66±3.26 |  | NK^56dim57-^/NK^56+^ | yes | 4 | 49.48±14.39 |  |
|  | no | 13 | 3.45±1.72 |  |  | no | 14 | 36.79±9.61 |  |
| NK^56dim57+^/Lym | yes | 4 | 2.07±1.06 |  | NK^56dim57+^/NK^56+^ | yes | 4 | 33.80±14.77 |  |
|  | no | 14 | 4.41±3.34 |  |  | no | 11 | 42.46±12.27 |  |
| NK^56dim57-16bri^/Lym | yes | 4 | 2.63±2.45 |  | NK^56dim57-16bri^/NK^56+^ | yes | 3 | 31.00±5.96 |  |
|  | no | 14 | 4.19±5.17 |  |  | no | 13 | 31.22±7.84 |  |
| NK^56dim57+16+^/Lym | yes | 4 | 2.03±1.01 |  | NK^56dim57+16+^/NK^56+^ | yes | 4 | 33.33±14.96 |  |
|  | no | 13 | 4.34±3.45 |  |  | no | 12 | 47.08±13.69 |  |
| CTLA-4^+^NK^56+^/Lym | yes | 4 | 0.43±0.27 |  | CTLA-4^+^NK^56+^/NK^56+^ | yes | 4 | 7.49±7.69 |  |
|  | no | 15 | 1.07±0.60 |  |  | no | 14 | 14.13±8.64 |  |
| PD-1^+^NK^56+^/Lym | yes | 4 | 0.09±0.07 |  | PD-1^+^NK^56+^/NK^56+^ | yes | 4 | 1.06±0.24 |  |
|  | no | 15 | 0.09±0.04 |  |  | no | 15 | 1.07±0.80 |  |
| PD-L1^+^NK^56+^/Lym | yes | 4 | 0.10±0.09 |  | PD-L1^+^NK^56+^/NK^56+^ | yes | 4 | 0.99±0.35 |  |
|  | no | 15 | 0.18±0.17 |  |  | no | 13 | 1.74±1.13 |  |
| PD-L2^+^NK^56+^/Lym | yes | 4 | 0.03±0.01 | borderline significant (*) | PD-L2^+^NK^56+^/NK^56+^ | yes | 4 | 0.99±0.35 |  |
|  | no | 15 | 0.04±0.03 |  |  | no | 13 | 1.74±1.13 |  |
| CD40L^+^NK^56+^/Lym | yes | 4 | 0.09±0.05 |  | CD40L^+^NK^56+^/NK^56+^ | yes | 4 | 2.06±1.66 |  |
|  | no | 15 | 0.16±0.15 |  |  | no | 15 | 2.26±1.91 |  |
| LAG3^+^NK^56+^/Lym | yes | 4 | 0.04±0.01 |  | LAG3^+^NK^56+^/NK^56+^ | yes | 4 | 1.15±1.18 |  |
|  | no | 15 | 0.04±0.02 |  |  | no | 15 | 0.87±1.01 |  |
| 2B4^+^NK^56+^/Lym | yes | 3 | 8.50±4.09 |  | 2B4^+^NK^56+^/NK^56+^ | yes | 4 | 93.58±6.03 | borderline significant (*) |
|  | no | 14 | 7.33±3.65 |  |  | no | 15 | 95.31±2.86 |  |
| TIM3^+^NK^56+^/Lym | yes | 3 | 4.80±2.64 |  |  |  |  |  |  |
|  | no | 14 | 4.95±2.53 |  |  |  |  |  |  |
| KLRG1^+^NK^56+^/Lym | yes | 4 | 1.27±0.71 |  | KLRG1^+^NK^56+^/NK^56+^ | yes | 4 | 22.04±10.25 |  |
|  | no | 14 | 2.64±2.95 |  |  | no | 13 | 24.28±10.87 |  |
| TIGIT^+^NK^56+^/Lym | yes | 4 | 4.32±2.61 |  | TIGIT^+^NK^56+^/NK^56+^ | yes | 4 | 65.45±8.44 |  |
|  | no | 14 | 4.31±2.47 |  |  | no | 14 | 58.31±12.09 |  |
| CIK^56+^/Lym | yes | 4 | 0.60±0.63 |  |  |  |  |  |  |
|  | no | 12 | 1.09±1.02 |  |  |  |  |  |  |
| CD40L^+^CIK^56+^/Lym | yes | 4 | 0.07±0.05 |  | CD40L^+^CIK^56+^/CIK^56+^ | yes | 3 | 10.23±8.81 |  |
|  | no | 15 | 0.07±0.06 |  |  | no | 13 | 14.91±11.17 |  |
| MAIT cells /Lym | yes | 4 | 0.06±0.06 |  |  |  |  |  |  |
|  | no | 15 | 0.49±1.80 |  |  |  |  |  |  |
| CTLA-4^+^CIK^56+^/Lym | yes | 4 | 0.07±0.02 |  | CTLA-4^+^CIK^56+^/CIK^56+^ | yes | 3 | 13.76±11.47 |  |
|  | no | 15 | 0.09±0.10 |  |  | no | 12 | 15.91±14.18 |  |
| PD-1^+^CIK^56+^/Lym | yes | 4 | 0.08±.,01 |  | PD-1^+^CIK^56+^/CIK^56+^ | yes | 3 | 12.99±7.55 |  |
|  | no | 15 | 0.06±0.04 |  |  | no | 12 | 10.33±10.56 |  |
| PD-L1^+^CIK^56+^/Lym | yes | 4 | 0.09±0.07 |  | PD-L1^+^CIK^56+^/CIK^56+^ | yes | 3 | 11.19±2.88 |  |
|  | no | 15 | 0.11±0.12 |  |  | no | 13 | 20.37±20.71 |  |
| PD-L2^+^CIK^56+^/Lym | yes | 4 | 0.03±0.02 |  | PD-L2^+^CIK^56+^/CIK^56+^ | yes | 4 | 9.28±7.81 |  |
|  | no | 15 | 0.04±0.04 |  |  | no | 13 | 11.81±14.72 |  |
| KLRG1^+^CIK^56+^/Lym | yes | 4 | 0.31±0.37 |  | KLRG1^+^CIK^56+^/CIK^56+^ | yes | 2 | 72.10±2.55 |  |
|  | no | 15 | 0.75±1.20 |  |  | no | 14 | 78.00±18.85 |  |
| TIGIT^+^CIK^56+^/Lym | yes | 4 | 0.16±0.17 |  | TIGIT^+^CIK^56+^/CIK^56+^ | yes | 3 | 38.10±21.48 |  |
|  | no | 15 | 0.24±0.32 |  |  | no | 11 | 40.71±17.14 |  |
| iNKT/Lym | yes | 4 | 0.42±0.72 | highly significant (***) |  |  |  |  |  |
|  | no | 15 | 0.01±0.02 |  |  |  |  |  |  |
| iNKT^3+^/Lym | yes | 4 | 0.29±0.54 | highly significant (***) | iNKT3^+^/6B11 | yes | 4 | 0.77±0.14 |  |
|  | no | 15 | 0.00±0.00 |  |  | no | 15 | 0.55±0.23 |  |
| iNKT^161+^/Lym | yes | 4 | 0.19±0.28 | highly significant (***) | iNKT^161+^/6B11 | yes | 4 | 0.17±0.13 |  |
|  | no | 15 | 0.02±0.09 |  |  | no | 15 | 0.30±0.18 |  |

# **Supplementary Table 8 — NoGvHD at baseline → developed relapse**

| Myeloid Cells | | | | | | | | | |
| --- | --- | --- | --- | --- | --- | --- | --- | --- | --- |
| In non Lym-Gate | | | | | In subsets | | | | |
| No GvHD/ Relapse after baseline | yes/no | N | % Mean  SD | *p* | No GvHD/ Relapse after baseline | yes/no | N | % Mean  SD | *p* |
| CD14^+^/non Lym | yes | 2 | 4.45±1.79 |  |  |  |  |  |  |
|  | no | 16 | 8.47±3.70 |  |  |  |  |  |  |
| CTLA-4^+^Mo/non Lym | yes | 2 | 0.52±0.28 |  | CTLA-4^+^Mo/Mo | yes | 2 | 35.40±16.97 |  |
|  | no | 16 | 1.48±1.35 |  |  | no | 16 | 37.06±17.84 |  |
| PD-1^+^Mo/non Lym | yes | 2 | 0.88±0.54 |  | PD-1^+^Mo/Mo | yes | 2 | 11.28±8.51 |  |
|  | no | 16 | 1.12±1.00 |  |  | no | 17 | 16.21±9.98 |  |
| PD-L1^+^Mo/non Lym | yes | 2 | 8.08±1.30 |  | PD-L1^+^Mo/Mo | yes | 2 | 97.75±0.21 |  |
|  | no | 16 | 7.29±4.61 |  |  | no | 16 | 97.79±3.14 |  |
| LAG3^+^Mo/non Lym | yes | 2 | 0.30±0.20 | Significant (**) | LAG3^+^Mo/Mo | yes | 2 | 3.91±3.03 | borderline significant (*) |
|  | no | 17 | 0.14±0.07 |  |  | no | 17 | 2.15±1.45 |  |
| 2B4^+^Mo/non Lym | yes | 2 | 7.34±1.00 |  | 2B4^+^Mo/Mo | yes | 2 | 99.65±0.07 |  |
|  | no | 16 | 8.08±4.07 |  |  | no | 17 | 99.66±0.67 |  |
| Tim3^+^Mo/non Lym | yes | 2 | 6.34±0.00 |  | Tim3^+^Mo/Mo | yes | 2 | 86.90±11.88 |  |
|  | no | 16 | 6.77±3.57 |  |  | no | 17 | 84.40±9.08 |  |
| DC^80+^/non Lym | yes | 2 | 1.73±1.33 |  |  |  |  |  |  |
|  | no | 17 | 1.08±0.74 |  |  |  |  |  |  |
| DC_mat_^80+^/non Lym | yes | 2 | 0.29±0.05 |  | DC_mat_^80+^/DC^80+^ | yes | 2 | 23.20±15.84 |  |
|  | no | 17 | 0.34±0.20 |  |  | no | 15 | 32.63±11.12 |  |
| DC_tol_^80+^/non Lym | yes | 2 | 0.43±0.04 |  | DC_tol_^80+^/DC^80+^ | yes | 1 | 31.10 |  |
|  | no | 17 | 0.52±0.46 |  |  | no | 16 | 49.96±13.72 |  |
| CTLA-4^+^DC^80+^/non Lym | yes | 2 | 0.34±0.09 |  | CTLA-4^+^DC^80+^/DC^80+^ | yes | 2 | 25.95±15.49 |  |
|  | no | 17 | 0.38±0.24 |  |  | no | 15 | 35.59±10.53 |  |
| CTLA-4^+^DC_mat_^80+^/non Lym | yes | 2 | 0.24±0.10 |  | CTLA-4^+^DC_mat_^80+^/DC_mat_^80+^ | yes | 2 | 92.25±5.87 |  |
|  | no | 17 | 0.26±0.15 |  |  | no | 16 | 81.04±10.10 |  |
| PMN-MDSC/non Lym | yes | 2 | 16.65±4.03 |  | PMN-MDSC/CD11b+CD33^+^ | yes | 2 | 77.45±2.90 |  |
|  | no | 16 | 18.28±8.88 |  |  | no | 15 | 70.45±8.49 |  |
| M-MDSC/non Lym | yes | 2 | 8.20±2.55 |  | M-MDSC/CD11b+CD33^+^ | yes | 2 | 22.55±2.90 |  |
|  | no | 16 | 8.51±4.08 |  |  | no | 16 | 31.70±11.89 |  |
| CTLA-4^+^PMN-MDSC/non Lym | yes | 2 | 3.89±2.72 | borderline significant (*) | CTLA-4^+^PMN-MDSC/PMN-MDSC | yes | 2 | 17.67±17.02 |  |
|  | no | 17 | 2.81±1.22 |  |  | no | 16 | 17.07±9.49 |  |
| CTLA-4^+^M-MDSC/non Lym | yes | 2 | 1.35±0.08 |  | CTLA-4^+^M-MDSC/PMN-MDSC | yes | 2 | 16.75±4.88 |  |
|  | no | 16 | 1.89±1.53 |  |  | no | 14 | 18.11±7.94 |  |
| Adaptive Cells | | | | | | | | | |
| In Lym-Gate | | | | | In subsets | | | | |
| No GvHD/ Relapse after baseline | yes/no | N | % Mean  SD | *p* | No GvHD/ Relapse after baseline | yes/no | N | % Mean  SD | *p* |
| T/Lym | yes | 2 | 12.74±8.10 |  |  |  |  |  |  |
|  | no | 16 | 11.05±7.29 |  |  |  |  |  |  |
| T^4+^/Lym | yes | 2 | 3.62±1.07 |  | T^4+^/T^3+^ | yes | 2 | 28.95±18.31 |  |
|  | no | 17 | 3.92±2.64 |  |  | no | 15 | 31.84±17.60 |  |
| T^4-/^Lym | yes | 2 | 13.49±13.46 |  | T^4-^/T^3+^ | yes | 2 | 71.05±18.31 |  |
|  | no | 16 | 8.86±7.42 |  |  | no | 15 | 70.72±18.10 |  |
| T_prol-early_ /Lym | yes | 2 | 3.95±5.30 | highly significant (***) | T_prol-early_ /T^3+^ | yes | 1 | 2.86 |  |
|  | no | 17 | 0.76±0.55 |  |  | no | 16 | 4.14±2.26 |  |
| T_prol-late_ /Lym | yes | 2 | 3.07±3.88 | highly significant (***) | T_prol-late_ /T^3+^ | yes | 1 | 4.47 |  |
|  | no | 17 | 0.99±0.53 |  |  | no | 14 | 5.31±3.36 |  |
| T_nn_ /Lym | yes | 1 | 3.33 |  | T_nn_ /T^3+^ | yes | 2 | 65.55±23.69 | significant (**) |
|  | no | 16 | 6.99±4.96 |  |  | no | 14 | 66.66±8.81 |  |
| T_em_ /Lym | yes | 2 | 98.90±1.41 |  | T_em_ /T^3+^ | yes | 2 | 65.00±24.32 |  |
|  | no | 14 | 85.35±29.47 |  |  | no | 15 | 65.62±14.59 |  |
| T_cm_ /Lym | yes | 2 | 1.10±1.37 |  | T_cm_ /T^3+^ | yes | 2 | 0.56±0.64 |  |
|  | no | 16 | 1.83±2.36 |  |  | no | 17 | 1.62±1.46 |  |
| CD28^+^T/Lym | yes | 2 | 10.17±6.13 |  | CD28^+^T/T^3+^ | yes | 2 | 56.10±4.24 |  |
|  | no | 16 | 7.96±5.76 |  |  | no | 13 | 77.37±18.29 |  |
| CD28^+^T^4+^/Lym | yes | 2 | 4.27±0.18 | borderline significant (*) | CD28^+^T^4+^/T^4+^ | yes | 2 | 92.05±5.87 |  |
|  | no | 17 | 4.33±2.77 |  |  | no | 17 | 94.89±6.31 |  |
| CD28^+^T^4-^/Lym | yes | 2 | 5.96±6.43 |  | CD28^+^T^4-^/T^4-^ | yes | 2 | 37.35±11.95 |  |
|  | no | 16 | 3.95±3.80 |  |  | no | 15 | 59.01±22.55 |  |
| CD40L^+^T^4+^/Lym | yes | 2 | 0.27±0.17 |  | CD40L^+^T/T^3+^ | yes | 2 | 2.37±1.82 |  |
|  | no | 17 | 0.20±0.27 |  |  | no | 16 | 2.24±2.33 |  |
| CD40L^+^T^4-^/Lym | yes | 2 | 0.09±0.11 |  | CD40L^+^T^4+^/T^4+^ | yes | 2 | 5.98±2.76 |  |
|  | no | 17 | 0.13±0.15 |  |  | no | 16 | 3.48±2.65 |  |
|  |  |  |  |  | CD40L^+^T^4-^/T^4-^ | yes | 2 | 0.50±0.38 |  |
|  |  |  |  |  |  | no | 15 | 1.29±1.40 |  |
| T^H1/Gui^/Lym | yes | 2 | 1.54±0.06 | borderline significant (*) | T^H1/Gui^/T^4+^ | yes | 0^a^ |  |  |
|  | no | 17 | 0.83±0.55 |  |  | no | 16 | 10.16±3.39 |  |
| T^H2/Gui^/Lym | yes | 2 | 0.31±0.20 |  | T^H2/Gui^/T^4+^ | yes | 2 | 4.66±2.82 |  |
|  | no | 17 | 0.71±0.50 |  |  | no | 15 | 8.05±3.91 |  |
| T^H17/Gui^/Lym | yes | 2 | 0.79±0.29 |  | T^H17/Gui^/T^4+^ | yes | 2 | 12.09±3.80 |  |
|  | no | 17 | 1.28±1.00 |  |  | no | 15 | 13.95±4.12 |  |
| γδ^+^T/Lym | yes | 2 | 0.45±0.29 |  | γδ^+^T/T^3+^ | yes | 2 | 1.66±1.34 |  |
|  | no | 17 | 0.35±0.43 |  |  | no | 15 | 2.87±2.68 |  |
| γδ^+^T^4+^/Lym | yes | 2 | 0.02±0.02 |  | γδ^+^T^4+^/T^3+^ | yes | 2 | 0.53±0,08 |  |
|  | no | 17 | 0.10±0.11 |  |  | no | 16 | 1.01±1.06 |  |
| γδ^+^T^4-^/Lym | yes | 2 | 0.14±0.19 |  | γδ^+^T^4-^/T^3+^ | yes | 2 | 12.21±13.00 | highly significant (***) |
|  | no | 16 | 0.32±0.44 |  |  | no | 17 | 1.57±1.47 |  |
| Intß7^+^T/Lym | yes | 2 | 5.27±1.00 | significant (**) | Intß7^+^T/T^3+^ | yes | 2 | 68.75±13.51 |  |
|  | no | 17 | 6.82±4.35 |  |  | no | 17 | 53.30±19.04 |  |
| Intß7^+^T^4+^/Lym | yes | 2 | 2.08±0.97 |  | Intß7^+^T^4+^/T^3+^ | yes | 2 | 38.05±13.79 |  |
|  | no | 15 | 1.43±0.94 |  |  | no | 14 | 16.75±13.56 |  |
| Intß7^+^T^4-^/Lym | yes | 1 | 1.84 |  | Intß7^+^T^4-^/T^3+^ | yes | 2 | 31.45±26.23 |  |
|  | no | 15 | 4.21±3.34 |  |  | no | 14 | 31.89±18.70 |  |
| CD127^+^T/Lym | yes | 1 | 6.51 |  | CD127^+^T/T^3+^ | yes | 2 | 37.65±19.30 |  |
|  | no | 17 | 7.26±4.82 |  |  | no | 17 | 47.64±17.16 |  |
|  |  |  |  |  | T^4+^_reg_ /T^4+^ | yes | 2 | 11.80±2.12 |  |
|  |  |  |  |  |  | no | 16 | 8.69±3.54 |  |
| T^4+^_reg_/Lym | yes | 2 | 0.56±0.13 |  | T^4+^_reg_ /T^3+^ | yes | 1 | 1.00 |  |
|  | no | 17 | 0.48±0.30 |  |  | no | 16 | 0.60±0.96 |  |
|  |  |  |  |  | T^4-^_reg_ /T^4-^ | yes | 2 | 3.49±2.05 |  |
|  |  |  |  |  |  | no | 17 | 3.57±2.79 |  |
| T^4-^_reg_/Lym | yes | 2 | 1.09±1.46 | highly significant (***) | T^4-^_reg_ /T^3+^ | yes | 1 | 0.52 |  |
|  | no | 17 | 0.07±0.17 |  |  | no | 14 | 0.18±0.18 |  |
| CTLA-4^+^T/Lym | yes | 2 | 0.28±0.35 | highly significant (***) | CTLA-4^+^T/T^3+^ | yes | 2 | 1.13±1.15 |  |
|  | no | 17 | 0.16±0.11 |  |  | no | 17 | 1.15±0.89 |  |
| CTLA-4^+^T^4+^/Lym | yes | 2 | 0.09±0.10 | borderline significant (*) | CTLA-4^+^T^4+^/T^4+^ | yes | 2 | 1.96±2.33 | highly significant (***) |
|  | no | 17 | 0.03±0.05 |  |  | no | 17 | 0.77±0.61 |  |
| CTLA-4^+^T^4-^/Lym | yes | 2 | 0.10±0.13 |  | CTLA-4^+^T^4-^/T^4-^ | yes | 2 | 0.53±0.45 |  |
|  | no | 17 | 0.11±0.09 |  |  | no | 17 | 1.06±0.60 |  |
| CD28^+^CTLA-4^+^T/Lym | yes | 1 | 0.43 |  | CD28^+^CTLA-4^+^T^4-^/T^4-^ | yes | 2 | 1.79±2.25 | highly significant (***) |
|  | no | 4 | 0.26±0.06 |  |  | no | 17 | 0.81±0.73 |  |
| CD28^+^CTLA-4^+^T^4+^/Lym | yes | 2 | 0.08±0.10 |  | CD28^+^CTLA-4^+^T^4+^/T^4+^ | yes | 2 | 0.56±0.42 |  |
|  | no | 17 | 0.04±0.05 |  |  | no | 17 | 0.84±0.73 |  |
| CD28^+^CTLA-4^+^T^4-^/Lym | yes | 2 | 0.10±0.13 |  | CD28^+^CTLA-4^+^T^4-^/T^4-^ |  |  |  |  |
|  | no | 17 | 0.08±0.10 |  |  |  |  |  |  |
| PD-1^+^T/Lym | yes | 2 | 4.93±5.83 |  | PD-1^+^T/T^3+^ | yes | 1 | 6.44 |  |
|  | no | 17 | 2.00±3.08 |  |  | no | 16 | 11.45±7.25 |  |
| PD-1^+^T^4+^/Lym | yes | 2 | 0.72±0.77 | highly significant (***) | PD-1^+^T^4+^/T^4+^ | yes | 2 | 17.15±4.45 |  |
|  | no | 17 | 0.26±0.21 |  |  | no | 15 | 17.18±9.75 |  |
| PD-1^+^T^4-^/Lym | yes | 2 | 4.22±5.05 | highly significant (***) | PD-1^+^T^4-^/T^4-^ | yes | 2 | 82.85±4.45 |  |
|  | no | 16 | 1.05±0.80 |  |  | no | 17 | 79.64±12.84 |  |
| PD-L1^+^T/Lym | yes | 2 | 0.90±0.52 |  | PD-L1^+^T/T^3+^ | yes | 2 | 4.61±0.57 | borderline significant (*) |
|  | no | 17 | 0.79±0.49 |  |  | no | 16 | 6.48±5.08 |  |
| PD-L1^+^T^4+^/Lym | yes | 2 | 0.42±0.07 | borderline significant (*) | PD-L1^+^T^4+^/T^4+^ | yes | 1 | 88.80 |  |
|  | no | 17 | 0.56±0.37 |  |  | no | 17 | 71.41±17.45 |  |
| PD-L1^+^T^4-^/Lym | yes | 2 | 0.48±0.59 |  | PD-L1^+^T^4-^/T^4-^ | yes | 2 | 41.05±42.21 | highly significant (***) |
|  | no | 17 | 0.23±0.20 |  |  | no | 14 | 34.00±13.88 |  |
| LAG3^+^T/Lym | yes | 2 | 0.13±0.12 | highly significant (***) | LAG3^+^T/T^3+^ | yes | 2 | 0.44±0.15 |  |
|  | no | 17 | 0.08±0.04 |  |  | no | 17 | 0.60±0.47 |  |
| LAG3^+^T^4+^/Lym | yes | 2 | 0.09±0.04 |  | LAG3^+^T^4+^/T^4+^ | yes | 1 | 0.98 |  |
|  | no | 17 | 0.05±0.03 |  |  | no | 16 | 1.09±0.54 |  |
| LAG3^+^T^4-^/Lym | yes | 2 | 0.17±0.19 | significant (**) | LAG3^+^T^4-^/T^4-^ | yes | 2 | 0.64±0.25 |  |
|  | no | 17 | 0.07±0.06 |  |  | no | 16 | 0.69±0.34 |  |
| 2B4^+^T/Lym | yes | 2 | 4.10±3.68 |  | 2B4^+^T/T^3+^ | yes | 2 | 19.05±7.99 |  |
|  | no | 16 | 3.53±4.26 |  |  | no | 17 | 21.07±17.28 |  |
| 2B4^+^T^4+^/Lym | yes | 2 | 0.12±0.11 |  | 2B4^+^T^4+^/T^4+^ | yes | 2 | 2.44±2.22 | highly significant (***) |
|  | no | 17 | 0.08±0.05 |  |  | no | 15 | 1.62±0.75 |  |
| 2B4^+^T^4-^/Lym | yes | 2 | 4.43±3.95 |  | 2B4^+^T^4-^/T^4-^ | yes | 2 | 30.10±3.54 |  |
|  | no | 16 | 3.49±4.32 |  |  | no | 14 | 33.31±16.60 |  |
| TIM3^+^T/Lym | yes | 2 | 0.33±0.08 |  | TIM3^+^T/T^3+^ | yes | 2 | 2.00±0.90 |  |
|  | no | 16 | 0.64±0.66 |  |  | no | 16 | 3.84±1.92 |  |
| TIM3^+^T^4+^/Lym | yes | 2 | 0.04±0.00 |  | TIM3^+^T^4+^/T^4+^ | yes | 2 | 0.90±0.03 |  |
|  | no | 17 | 0.06±0.04 |  |  | no | 17 | 1.46±0.72 |  |
| TIM3^+^T^4-^/Lym | yes | 2 | 0.26±0.12 |  | TIM3^+^T^4-^/T^4-^ | yes | 2 | 2.40±1.34 | borderline significant (*) |
|  | no | 17 | 0.55±0.65 |  |  | no | 16 | 5.53±3.28 |  |
| KLRG1^+^T/Lym | yes | 2 | 7.20±4.53 |  | KLRG1^+^T/T^3+^ | yes | 2 | 41.35±4.17 |  |
|  | no | 17 | 6.55±5.94 |  |  | no | 15 | 42.03±14.37 |  |
| KLRG1^+^T^4+^/Lym | yes | 2 | 0.38±0.49 |  | KLRG1^+^T^4+^/T^4+^ | yes | 2 | 9.11±12.43 |  |
|  | no | 17 | 0.60±0.53 |  |  | no | 16 | 10.06±7.14 |  |
| KLRG1^+^T^4-^/Lym | yes | 2 | 6.79±4.96 |  | KLRG1^+^T^4-^/T^4-^ | yes | 2 | 90.90±12.45 |  |
|  | no | 17 | 5.95±5.56 |  |  | no | 16 | 89.95±7.15 |  |
| TIGIT^+^T/Lym | yes | 2 | 10.93±8.44 |  | TIGIT^+^T/T^3+^ | yes | 2 | 50.90±11.03 |  |
|  | no | 17 | 7.66±7.35 |  |  | no | 17 | 44.98±16.23 |  |
| TIGIT^+^T^4+^/Lym | yes | 2 | 1.91±0.37 |  | TIGIT^+^T^4+^/T^4+^ | yes | 2 | 22.90±14.28 |  |
|  | no | 17 | 1.26±0.91 |  |  | no | 15 | 21.57±13.86 |  |
| TIGIT^+^T^4-^/Lym | yes | 2 | 9.06±8.12 |  | TIGIT^+^T^4-^/T^4-^ | yes | 2 | 22.90±14.28 |  |
|  | no | 16 | 5.42±5.75 |  |  | no | 15 | 21.57±13.86 |  |
| B/Lym | yes | 2 | 0.10±0.01 |  |  |  |  |  |  |
|  | no | 17 | 0.08±0.19 |  |  |  |  |  |  |
| B_nai_/Lym | yes | 2 | 8.11±0.96 |  | B_nai_/B | yes | 2 | 82.80±3.11 |  |
|  | no | 16 | 3.21±2.96 |  |  | no | 17 | 76.18±19.07 |  |
| B_mem(class)_/Lym | yes | 2 | 1.00±0.22 |  | B_mem(class)_/B | yes | 2 | 10.27±3.02 |  |
|  | no | 17 | 0.25±0.21 |  |  | no | 17 | 10.58±13.73 |  |
| B_mem_/Lym | yes | 2 | 0.13±0.06 |  | B_mem_/B | yes | 2 | 1.25±0.51 |  |
|  | no | 17 | 0.08±0.05 |  |  | no | 17 | 2.37±2.12 |  |
| B_reg_/Lym | yes | 2 | 0.51±0.06 |  | B_reg_/B | yes | 2 | 4.60±1.62 |  |
|  | no | 17 | 0.22±0.15 |  |  | no | 17 | 6.26±4.31 |  |
| Innate Cells | | | | | | | | | |
| In Lym-Gate | | | | | In subsets | | | | |
| No GvHD/ Relapse after baseline | yes/no | N | % Mean  SD | *p* | No GvHD/ Relapse after baseline | nes/no | N | % Mean  SD | *p* |
| NK^56+^/Lym | yes | 2 | 0.77±1.03 |  |  |  |  |  |  |
|  | no | 17 | 0.40±1.11 |  |  |  |  |  |  |
| NK^56bri^/Lym | yes | 2 | 0.50±0.51 |  | NK^56bri^/NK^56+^ | yes | 2 | 14.92±4.40 |  |
|  | no | 15 | 1.64±1.78 |  |  | no | 15 | 16.47±9.49 |  |
| NK^56bri16-^/Lym | yes | 2 | 0.33±0.09 |  | NK^56bri16-^/NK^56+^ | yes | 2 | 5.04±4.39 |  |
|  | no | 15 | 0.50±0.38 |  |  | no | 15 | 8.60±7.21 |  |
| NK^56bri16+^/Lym | yes | 2 | 0.90±0.60 |  | NK^56bri16+^/NK^56+^ | yes | 2 | 9.89±0.01 |  |
|  | no | 15 | 0.99±1.21 |  |  | no | 15 | 10.48±7.86 |  |
| NK^56dim16+^/Lym | yes | 2 | 7.20±4.96 |  | NK^56dim16+^/NK^56+^ | yes | 2 | 79.10±1.98 | borderline significant (*) |
|  | no | 16 | 6.86±4.09 |  |  | no | 14 | 77.82±10.45 |  |
| NK^56bri57-^/Lym | yes | 2 | 1.13±0.38 |  | NK^56bri57-^/NK^56+^ | yes | 2 | 14.30±5.37 |  |
|  | no | 15 | 1.77±1.98 |  |  | no | 13 | 14.35±7.04 |  |
| NK^56dim57-^/Lym | yes | 2 | 4.99±4.88 | highly significant (***) | NK^56dim57-^/NK^56+^ | yes | 2 | 47.90±21.92 |  |
|  | no | 15 | 3.30±1.66 |  |  | no | 16 | 38.57±10.60 |  |
| NK^56dim57+^/Lym | yes | 2 | 2.90±0.81 |  | NK^56dim57+^/NK^56+^ | yes | 2 | 37.60±16.26 |  |
|  | no | 16 | 4.02±3.29 |  |  | no | 13 | 40.55±13.23 |  |
| NK^56dim57-16bri^/Lym | yes | 2 | 3.70±3.62 |  | NK^56dim57-16bri^/NK^56+^ | yes | 1 | 24.00 |  |
|  | no | 16 | 3.86±4.89 |  |  | no | 15 | 31.66±7.34 |  |
| NK^56dim57+16+^/Lym | yes | 2 | 2.84±0.73 |  | NK^56dim57+16+^/NK^56+^ | yes | 2 | 37.00±16.69 |  |
|  | no | 15 | 3.92±3.37 |  |  | no | 14 | 44.59±14.99 |  |
| CTLA-4^+^NK^56+^/Lym | yes | 2 | 0.61±0.47 |  | CTLA-4^+^NK^56+^/NK^56+^ | yes | 2 | 14.21±17.53 | borderline significant (*) |
|  | no | 17 | 0.97±0.62 |  |  | no | 16 | 12.46±8.05 |  |
| PD-1^+^NK^56+^/Lym | yes | 2 | 0.11±0.12 | highly significant (***) | PD-1^+^NK^56+^/NK^56+^ | yes | 2 | 1.01±0.33 |  |
|  | no | 17 | 0.09±0.04 |  |  | no | 17 | 1.07±0.75 |  |
| PD-L1^+^NK^56+^/Lym | yes | 2 | 0.15±0.11 |  | PD-L1^+^NK^56+^/NK^56+^ | yes | 2 | 1.70±0.42 |  |
|  | no | 17 | 0.16±0.16 |  |  | no | 15 | 1.54±1.11 |  |
| PD-L2^+^NK^56+^/Lym | yes | 2 | 0.03±0.02 |  | PD-L2^+^NK^56+^/NK^56+^ | yes | 2 | 1.70±0.42 |  |
|  | no | 17 | 0.04±0.03 |  |  | no | 15 | 1.54±1.11 |  |
| CD40L^+^NK^56+^/Lym | yes | 2 | 0.07±0.05 |  | CD40L^+^NK^56+^/NK^56+^ | yes | 2 | 1.89±2.35 |  |
|  | no | 17 | 0.15±0.14 |  |  | no | 17 | 2.25±1.83 |  |
| LAG3^+^NK^56+^/Lym | yes | 2 | 0.06±0.01 |  | LAG3^+^NK^56+^/NK^56+^ | yes | 2 | 1.75±1.65 |  |
|  | no | 17 | 0.04±0.02 |  |  | no | 17 | 0.83±0.95 |  |
| 2B4^+^NK^56+^/Lym | yes | 2 | 4.41±0.35 |  | 2B4^+^NK^56+^/NK^56+^ | yes | 2 | 87.75±4.31 |  |
|  | no | 15 | 7.96±3.66 |  |  | no | 17 | 95.79±2.49 |  |
| TIM3^+^NK^56+^/Lym | yes | 2 | 2.06±0.45 |  | TIM3^+^NK^56+^/NK^56+^ | yes | 1 | 38.00 |  |
|  | no | 15 | 5.31±2.36 |  |  | no | 1 | 55.00 |  |
| KLRG1^+^NK^56+^/Lym | yes | 2 | 1.25±0.09 |  | KLRG1^+^NK^56+^/NK^56+^ | yes | 2 | 23.64±21.16 | borderline significant (*) |
|  | no | 16 | 2.47±2.80 |  |  | no | 15 | 23.77±9.64 |  |
| TIGIT^+^NK^56+^/Lym | yes | 2 | 5.15±4.29 |  | TIGIT^+^NK^56+^/NK^56+^ | yes | 2 | 60.40±10.61 |  |
|  | no | 16 | 4.20±2.31 |  |  | no | 16 | 59.84±12.00 |  |
| CIK^56+^/Lym | yes | 2 | 0.40±0.31 |  |  |  |  |  |  |
|  | no | 14 | 1.04±0.98 |  |  |  |  |  |  |
| CD40L^+^CIK^56+^/Lym | yes | 2 | 0.07±0.07 |  | CD40L^+^CIK^56+^/CIK^56+^ | yes | 2 | 13.24±14.65 |  |
|  | no | 17 | 0.07±0.06 |  |  | no | 14 | 14.15±10.71 |  |
| MAIT cells /Lym | yes | 2 | 3.51±4.94 | highly significant (***) |  |  |  |  |  |
|  | no | 17 | 0.03±0.04 |  |  |  |  |  |  |
| CTLA-4^+^CIK^56+^/Lym | yes | 2 | 0.03±0.02 |  | CTLA-4^+^CIK^56+^/CIK^56+^ | yes | 2 | 6.89±0.30 | significant (**) |
|  | no | 17 | 0.09±0.09 |  |  | no | 13 | 16.80±13.89 |  |
| PD-1^+^CIK^56+^/Lym | yes | 2 | 0.05±0.05 |  | PD-1^+^CIK^56+^/CIK^56+^ | yes | 2 | 8.23±4.49 |  |
|  | no | 17 | 0.06±0.04 |  |  | no | 13 | 11.27±10.49 |  |
| PD-L1^+^CIK^56+^/Lym | yes | 2 | 0.04±0.02 |  | PD-L1^+^CIK^56+^/CIK^56+^ | yes | 2 | 9.53±2.22 |  |
|  | no | 17 | 0.11±0.11 |  |  | no | 14 | 19.95±19.95 |  |
| PD-L2^+^CIK^56+^/Lym | yes | 2 | 0.02±0.01 |  | PD-L2^+^CIK^56+^/CIK^56+^ | yes | 2 | 6.35±2.80 |  |
|  | no | 17 | 0.04±0.04 |  |  | no | 15 | 11.86±13.99 |  |
| LAG3^+^CIK^56+^/Lym | yes | 1 | 0.15 |  | LAG3^+^CIK^56+^/CIK^56+^ | yes | 0^a^ | 4.00 |  |
|  | no | 1 | 0.00 |  |  | no | 1 |  |  |
| 2B4^+^CIK^56+^/Lym | yes | 0^a^ |  |  | 2B4^+^CIK^56+^/CIK^56+^ | yes | 0^a^ | 83.00±8.68 |  |
|  | no | 0^a^ |  |  |  | no | 4 |  |  |
| TIM3^+^CIK^56+^/Lym | yes | 0^a^ |  |  | TIM3^+^CIK^56+^/CIK^56+^ | yes | 0^a^ | 43.00±18.38 |  |
|  | no | 0^a^ |  |  |  | no | 2 |  |  |
| KLRG1^+^CIK^56+^/Lym | yes | 2 | 0.19±0.09 |  | KLRG1^+^CIK^56+^/CIK^56+^ | yes | 1 | 68.50 |  |
|  | no | 17 | 0.71±1.14 |  |  | no | 15 | 77.85±18.14 |  |
| TIGIT^+^CIK^56+^/Lym | yes | 2 | 0.09±0.04 |  | TIGIT^+^CIK^56+^/CIK^56+^ | yes | 1 | 65.70 |  |
|  | no | 17 | 0.24±0.30 |  |  | no | 13 | 38.18±16.27 |  |
| iNKT/Lym | yes | 2 | 0.79±1.01 | highly significant (***) |  |  |  |  |  |
|  | no | 17 | 0.01±0.02 |  |  |  |  |  |  |
| iNKT^3+^/Lym | yes | 2 | 0.56±0.76 | highly significant (***) | iNKT^3+^/6B11 | yes | 2 | 0.65±0.18 |  |
|  | no | 17 | 0.00±0.01 |  |  | no | 17 | 0.59±0.24 |  |
| iNKT^161+^/Lym | yes | 2 | 0.30±0.42 | highly significant (***) | iNKT^161+^/6B11 | yes | 2 | 0.26±0.12 |  |
|  | no | 17 | 0.03±0.08 |  |  | no | 17 | 0.27±0.19 |  |

**Supplementary Table 9. Detailed patient and transplant characteristics per cohort.**

Per-patient clinical and transplant data underlying the summary in Table 1 of the main manuscript.

**Supplementary Table 9a. Patients with chronic GvHD (cGvHD).**

| Sample number | Sex, Age [years] | Initial Diagnosis | Donor type | Conditioning | GvHD prophylaxis | Day of SCT | Immune suppression after SCT | Date blood sample | For cGvHD: de novo/quiescent or progressive? | Affected organs | Immunosuppressive medication |
| --- | --- | --- | --- | --- | --- | --- | --- | --- | --- | --- | --- |
| 1689 | M, 67 | AML | MUD | RIC | ATG | 21.09.2022 |  | 15.01.2024 | Progressive | skin | Prednisolone, Ruxolitinib |
| 1734 | M, 59 | AML | MMUD | RIC | ATG 30 | 16.07.2021 | Tacrolimus, MMF | 22.10.2025 | de novo | Liver, Skin | Prednisolone, Ciclosporin, Ruxolitinib |
| 1743 | F, 71 | AML | MUD | RIC | ATG 30 | 18.01.2023 | Tacrolimus, MMF | 20.01.2025 | quiescent | Lung | MMF |
| 1752 | F, 72 | AML | MUD, 10/10 | RIC | ATG 30 | 16.04.2024 | Ciclosporin, MMF | 26.06.2025 | de novo, atypic | Pleura-/ pericardial effusion | Prednisolone 40mg |
| 1751 | M, 79 | AML | MRD |  |  | 28.03.2017 |  | 02.06.2025 | quiescent | polyserositis (Pleura, pericard, Ascites), eyes, Myocarditis | Ruxolitinib 2x10 |
| 1744 | M, 31 | AML | Haplo | RIC | PTCy 80 | 13.06.2024 | Tacrolimus, MMF | 21.01.2025 | quiescent | Skin | yes, prednisolone 30mg, Tacrolimus/Posaconazole |
| 1742 | M, 21 | AML | Haplo | RIC | PTCy 80 | 14.08.2024 | Tacrolimus, MMF | 13.12.2024 | quiescent | Skin, Liver | no |
| 1701 | F, 69 | AML | MUD | MAC | ATG 30 | 20.04.2022 | Ciclosporin, MMF | 15.04.2024 | de novo | Muscles/Joints |  |
| 1709 | M, 60 | AML | MUD | RIC | ATG 30 | 19.10.2023 | Tacrolimus, MMF | 16.05.2025 | quiescent | Lower GI, Skin | Prednisolone 5, CSA 200/Posaconazole |
| 1698 | M, 70 | MDS | MMUD | RIC | PTCy 80 | 18.08.2023 | Tacrolimus, MMF | 03.04.2025 | quiescent | Skin | Prednisolone 5mg, Tacrolimus/Posaconazole 2,5mg, Ruxolitinib 20mg |
| 1745 | F, 36 | ALL | Haplo | RIC | PTCy 100 | 18.08.2021 | Tacrolimus, MMF | 28.01.2025 | progressive | Lung, eyes, mouth, vagina | Prednisolone, Ruxolitinib |
| 1753 | F, 55 | ALL | MUD, 10/10 | MAC | ATG 30 | 29.12.2022 | Ciclosporin, MMF | 04.07.2025 | de novo | Mouth, eyes, upper GI, Lung, Liver | Methylprednisolon 24mg, Tacrolimus 1mg/d, Montelukast, Trimbow |
| 1739 | F, 18 | ALL | MUD, 10/10 | MAC | ATG 30 | 27.12.2022 | Ciclosporin, MMF | 11.11.2024 | de novo | Skin, Lung, Eyes, Genitals, Mouth, GI | Ruxolitinib 20mg, Montelukast, Everolimus 3mg, Hydrokortison |
| 1746 | M, 66 | MDS | MUD | RIC | ATG 30 | 05.06.2024 | Ciclosporin, MMF | 04.03.2025 | de novo chronic | Mouth, skin | Methylprednisolone, Everolimus, Ruxolitinib |

**Supplementary Table 9b. Patients with overlap acute/chronic GvHD (overlap a/cGvHD).**

| Sample number | Sex, Age [years] | Initial Diagnosis | Donor type | Conditioning | GvHD prophylaxis | Day of SCT | Immune suppression after SCT | Date blood sample | For cGvHD: de novo/quiescent or progressive? | Affected organs | Immunosuppressive medication |
| --- | --- | --- | --- | --- | --- | --- | --- | --- | --- | --- | --- |
| 1754 | F, 22 | AML | MUD | RIC | ATG | 17.03.2025 | Ciclosporin, MMF | 28.07.2025 | progressive | Lower GI tract | Prednisolone 0,7mg/kg, Ciclosporin in standard dosis |
| 1747 | M, 46 | AML | MMUD, 9/10 | MAC | ATG 60 | 03.06.2024 | Ciclosporin, MMF | 18.03.2025 | progressive / Overlap | Skin, upper GI, Joints, Mouth | Prednisolone, Tacrolimus/Posaconazole, Ruxolitinib, MMF |
| 1748 | M, 56 | MDS | Haplo | RIC | PTCy 100 | 26.07.2024 | Tacrolimus, MMF | 28.03.2025 | progressive | Skin | Prednisolone, Tacrolimus/Posaconazole, Ruxolitinib |
| 1750 | M, 68 | MDS | MUD | RIC | ATG 30 | 17.10.2023 | Ciclosporin, MMF | 05.05.2025 | de novo overlap cGvHD | Skin; GI, Mouth, genitals, eyes | Ruxolitinib 20mg |

**Supplementary Table 9c. Patients with acute GvHD (aGvHD).**

| Sample number | Sex, Age [years] | Initial Diagnosis | Donor type | Conditioning | GvHD prophylaxis | Day of SCT | Immune suppression after SCT | Date blood sample | For cGvHD: de novo/quiescent or progressive? | Affected organs | Immunosuppressive medication |
| --- | --- | --- | --- | --- | --- | --- | --- | --- | --- | --- | --- |
| 1749 | M, 33 | AML | MMUD, 9/10 | RIC | ATG 60 | 29.10.2024 | Ciclosporin, MMF | 22.04.2025 | de novo acute | lower GI | Prednisolone, Tacrolimus, Ruxolitinib |
| 1708 | F, 74 | AML | Haplo | RIC | PTCy 100 | 10.01.2024 | Tacrolimus, MMF | 26.04.2024 |  | Lower GI tract | no |
| 1741 | M, 66 | AML | Haplo | RIC | PTCy 80 | 16.10.2024 | Tacrolimus, MMF | 28.11.2024 |  | Skin | no |
| 1712 | M, 53 | MDS | MMUD, 9/10 | RIC | ATG 60 | 29.02.2024 | Ciclosporin, MMF | 21.05.2024 | de novo acute | Skin, lower GI | Methylprednisolone, Everolimus, Ruxolitinib |
| 1740 | M, 74 | MDS | MUD | RIC | PTCy 100 | 27.08.2024 | Tacrolimus, MMF | 25.11.2024 |  | skin | Prednisolone 0,75mg/kg, Tacrolimus/Posaconazole |
| 1715 | M, 73 | primary Myelofibrosis | MUD, 10/10 | RIC | ATG 30 | 02.01.2024 | Ciclosporin, MMF | 12.06.2024 | de novo acute | Upper + lower GI | Methylprednisolon 2mg/Kg, Tacrolimus/posa |

**Supplementary Table 9d. Patients with without GvHD (NoGvHD).**

| Sample number | Sex, Age [years] | Initial Diagnosis | Donor type | Conditioning | GvHD prophylaxis | Day of SCT | Immune suppression after SCT | Date blood sample | Affected organs | Immunosuppressive medication |
| --- | --- | --- | --- | --- | --- | --- | --- | --- | --- | --- |
| 1728 | F, 61 | AML | MUD | RIC | PTCy 100 | 15.03.2023 | Tacrolimus, MMF | 25.09.2024 | na | no IS |
| 1719 | M, 62 | AML | MUD | RIC | ATG 60 | 28.03.2024 | Ciclosporin, MMF | 04.07.2024 | na | no IS |
| 1722 | F, 20 | AML | MRD | RIC | ATG 30 | 05.04.2024 | Ciclosporin, MMF | 12.09.2024 | na | no IS |
| 1725 | F, 76 | AML | MUD | RIC | ATG 60 | 31.03.2023 | Tacrolimus, MMF | 17.09.2024 | na | no IS |
| 1727 | M, 77 | AML | MUD | RIC | ATG 60 | 17.11.2022 | Ciclosporin, MMF | 24.09.2024 | na | no IS |
| 1729 | M, 71 | AML | MUD | RIC | ATG 60 | 01.09.2023 | Ciclosporin, MMF | 26.09.2024 | na | no IS |
| 1730 | M, 70 | AML | MRD | RIC | ATG 30 | 28.09.2022 | Ciclosporin, MMF | 01.10.2024 | na | no IS |
| 1714 | M, 70 | AML | MUD | RIC | ATG 60 | 26.01.2024 | Ciclosporin, MMF | 11.06.2024 | na | no IS |
| 1717 | M, 71 | AML | MUD | RIC | ATG 60 | 28.02.2024 | Ciclosporin, MMF | 17.06.2024 | na | no IS |
| 1718 | M, 70 | AML | Haplo | RIC | PTCy 100 | 08.03.2024 | Tacrolimus, MMF | 27.06.2024 | na | no IS |
| 1731 | M, 60 | AML | Haplo | RIC | PTCy 100 | 19.10.2023 | Tacrolimus, MMF | 01.10.2024 | na | no IS |
| 1713 | M, 69 | AML | MUD | RIC | PTCy 100 | 02.01.2024 | Tacrolimus, MMF | 04.06.2024 | na | no IS |
| 1733 | M, 64 | AML | Haplo | RIC | PTCy 100 | 05.07.2024 | Tacrolimus, MMF | 15.10.2024 | na | no IS |
| 1726 | F, 65 | MDS | MUD | RIC | ATG 60 | 20.10.2023 | Ciclosporin, MMF | 18.09.2024 | na | no IS |
| 1735 | M, 63 | MDS | MUD | RIC | PTCy 100 | 28.05.2024 | Tacrolimus, MMF | 23.10.2024 | na | no IS |
| 1737 | M, 62 | MDS | MRD | RIC | ATG 30 | 05.07.2023 | Ciclosporin, MMF | 05.11.2024 | na | no IS |
| 1732 | M, 66 | MDS | MUD | RIC | ATG 60 | 26.05.2023 | Tacrolimus, MMF | 01.10.2024 | na | Ruxolitinib 10mg |
| 1724 | M, 33 | BPDCN | Haplo | RIC | PTCy 100 | 07.09.2023 | Tacrolimus, MMF | 17.09.2024 | na | no IS |
| 1736 | M, 63 | ALL | MUD | RIC | ATG 60 | 21.12.2022 | Ciclosporin, MMF | 24.10.2024 | na | no IS |

**Supplementary Table 9e. Patients with healthy donors.**

| Sample number | Sex, Age [years] |
| --- | --- |
| 1700 | M, 71 |
| 1702 | F, 85 |
| 1703 | M, 62 |
| 1704 | M, 23 |
| 1705 | F, 31 |
| 1706 | F, 22 |
| 1707 | F, 63 |
| 1710 | F, 51 |
| 1711 | F, 52 |
| 1716 | M, 57 |
